# Supplementary material for: N-aryl pyrido cyanine derivatives are nuclear and organelle DNA markers for two-photon and super-resolution imaging
Source: Nat Commun. 2021 May 11;12:2650. doi: 10.1038/s41467-021-23019-w (PMC8113587; doi:10.1038/s41467-021-23019-w)
Supplement: Supplementary file 1 — Supplementary Information [file 41467_2021_23019_MOESM1_ESM.pdf]

# Supplementary Materials for

## N-Aryl Pyrido Cyanine derivatives are nuclear and organelle DNA markers for two-photon and super-resolution imaging

Kakishi Uno, Nagisa Sugimoto, Yoshikatsu Sato*
Correspondence to: sato.yoshikatsu@i.mbox.nagoya-u.ac.jp

**This PDF file includes:**

Supplementary Text
Supplementary Figs. 1 to 13
NMR charts 1-58

**Other Supplementary Materials for this manuscript include the following:**

Supplementary Movies 1 to 6

**Supplementary Note 1**: Synthesis of **PC1**, **PC2**, **PC3**, and **PC4**.

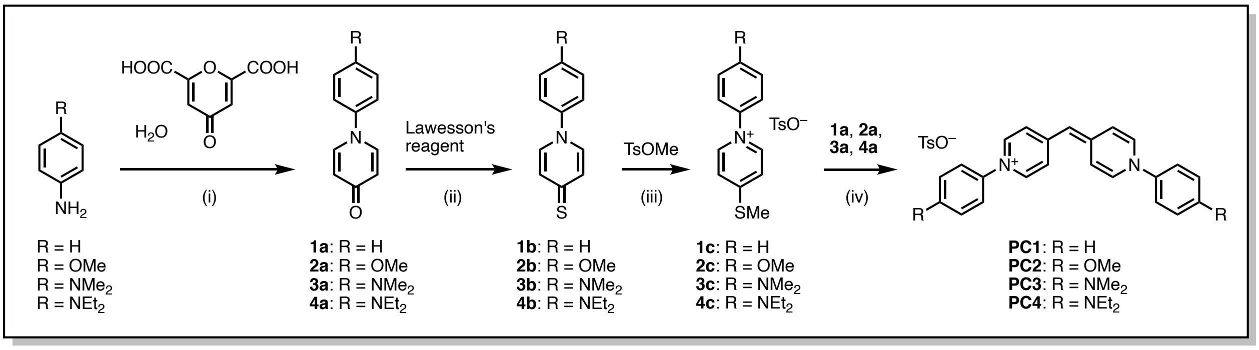


Scheme S1. (i) Chelidonic acid monohydrate, aniline derivatives (1.1 equiv.), DMSO, 170 °C, 2 h; (ii) Lawesson's reagent, toluene/1,4-dioxane (= 10/1), reflux, 3–4 h; (iii) TsOMe, 1,4-dioxane, reflux, 2 h; (iv) **1a, 2a, 3a**, and **4a** in dry THF, MeLi, –0 °C. $CH_2Cl_2$/triethylamine, 70 °C, reflux, 1 d.

**1-Phenylpyridin-4(1*H*)-one (1a)**

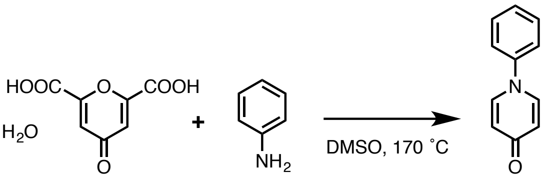


**1a**

Chelidonic acid monohydrate (3.00 g, 14.8 mmol) and aniline (1.45 g, 15.6 mmol, 1.05 equiv.) were dissolved in DMSO (45 mL). The mixture was stirred at 170 ℃ under open air for 2 h. After cooling back to room temperature, DMSO was removed *in vacuo*. The resulting dark residue was subjected to column chromatography on silica gel (eluent: $CH_2Cl_2$/MeOH = 95/5 to 80/20; *v/v*). Recrystallization from methanol and ether gave **1a** as a pale yellow solid (1.45 g, 57%).

$^1$H NMR (400 MHz, $(CD_3)_2SO$): δ 6.23 (d, *J* = 7.6 Hz, 2H), 7.40–7.48 (m, 1H), 7.54 (s, *2*H), 7.55 (s, 2H), 7.99 (d, *J* = 8.4 Hz, 2H). $^{13}$C NMR (100 MHz, $(CD_3)_2SO$): δ 117.96, 122.60, 127.87, 129.98, 139.91, 142.76, 177.42. HRMS (ESI positive mode) *m/z* calculated for $C_{11}H_9NNaO$ $[M+Na]^+$: 194.0576, found: 194.0574. Mp = 78.2–79.0 ℃.

**1-Phenylpyridine-4(1*H*)-thione (1b)**

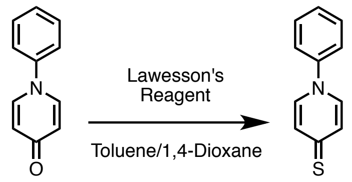


**1a** **1b**

A solution of **1a** (1.00 g, 5.84 mmol) and Lawesson's reagent (2.48 g, 6.13 mmol, 1.05 equiv.) in toluene (50 mL) and 1,4-dioxane (5.0 mL) was stirred at refluxing temperature for 4 h. After cooling back to room temperature, hexane (20 mL) was added to the mixture and the formed solid was filtered. The crude product was purified by column chromatography on silica gel (eluent: $CH_2Cl_2$/MeOH = 99/1 to 70/30; *v/v*). Recrystallization from methanol and ether gave **1b** as a yellow solid (569 mg, 52%).

$^1$H NMR (400 MHz, $(CD_3)_2SO$): δ 7.26 (d, *J* = 7.6 Hz, 2H), 7.51 (t, *J* = 7.0 Hz, 1H), 7.54–7.56 (m, 4H), 7.92 (d, *J* = 7.2 Hz, 2H). $^{13}$C NMR (100 MHz, $(CD_3)_2SO$): δ122.83, 128.83, 130.11, 130.32, 134.84, 142.39, 191.16. HRMS (ESI positive mode) *m/z* calculated for $C_{11}H_9NNaS$ $[M+Na]^+$: 210.0348, found: 210.0345. Mp = 149.5–150.2 ℃.

**4-(Methylthio)-1-phenylpyridin-1-ium 4-methylbenzenesulfonate (1c)**

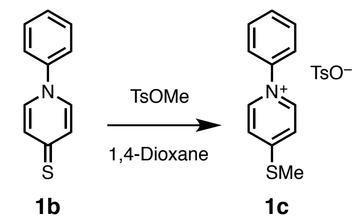

A solution of **1b** (420 mg, 2.24 mmol) and methyl *p*-toluenesulfonate (438 mg, 2.35 mmol, 1.05 equiv.) in 1,4-dioxane (20 mL) was stirred at refluxing temperature for 2 h. After cooling back to room temperature, ether (20 mL) was added to the mixture. The upper layer was separated, and the bottom layer containing **1c** was washed with ether twice. The resulting oil was dried *in vacuo* for 2 h. The obtained crude **1c** (820 mg, 98%) was used to the next reaction without further purification.

**1-Phenyl-4-((1-phenylpyridin-4(1*H*)-ylidene)methyl)pyridin-1-ium4-methylbenzenesulfonate (PC1)**

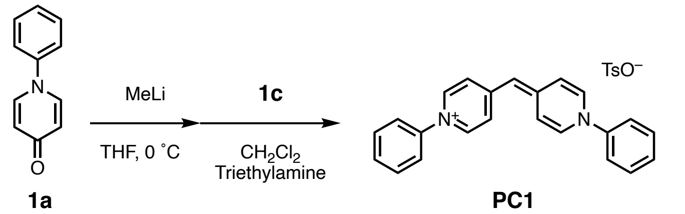

A solution of **1a** (300 mg, 1.75 mmol) in THF (20 mL) was cooled to 0 °C by an ice bath under nitrogen atmosphere. 3 M MeLi solution in diethoxymethane (1.2 mL, 3.6 mmol, 2.0 equiv.) was slowly added dropwise using a syringe. The ice bath was removed and the mixture was warmed to room temperature gradually. After additional stirring for 20 min, a solution of **1c** (720 mg, 1.93 mmol, 1.1 equiv.) and triethylamine (4.0 mL) in $CH_2Cl_2$ (20 mL) was added using a syringe at 0 °C. Then, the mixture was stirred at 70 °C for 1 day under nitrogen atmosphere. After cooling back to room temperature, the solvent was removed *in vacuo*. The resulting dark red solid was subjected to column chromatography on silica gel (eluent: $CH_2Cl_2$/MeOH = 99/5 to 80/20; *v/v*). The obtained red solid was suspended to minimum amount of acetone and sonicated for 5 min, and then ether was further added. The formed solid was filtered to give **PC1** as a red solid (355 mg, 41%).

$^1$H NMR (400 MHz, (CD3)2SO): δ 2.27 (s, 3H), 5.82 (s, 1H), 7.09 (d, J = 8.4 Hz, 2H), 7.18–7.40 (br, 4H), 7.46 (d, *J* = 8.4 Hz, 2H), 7.54 (t, *J* = 6.8 Hz, 2H), 7.58–7.72 (m, 8H), 8.19 (d, *J* = 7.6 Hz, 4H). $^{13}$C NMR (100 MHz, $(CD_3)_2SO$): δ 20.74, 100.91, 117.04 (br), 122.77, 125.46, 127.98, 128.82, 130.11, 137.46, 138.39, 142.16, 145.87, 149.61. HRMS (ESI positive mode) calculated for $C_{23}H_{19}N_2$ $[M]^+$: 323.1543, found: 323.1537. Mp = 214.1–215.0 °C.

**1-(4-Methoxyphenyl)pyridin-4(1*H*)-one (2a)**

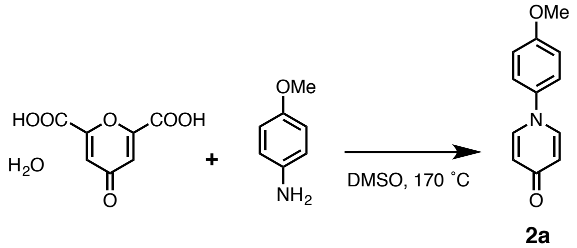

Chelidonic acid monohydrate (1.00 g, 4.95 mmol) and *p*-anisidine (640 mg, 5.2 mmol, 1.05 equiv.) were dissolved in DMSO (20 mL). The mixture was stirred at 170 °C under open air for 2 h. After cooling back to room temperature, DMSO was removed *in vacuo*. The resulting solid was suspended in ethyl acetate (30 mL) and vigorously stirred for 2 h. After addition of ether (30 mL), the solid was filtered and dried to give **2a** as a pale yellow solid (863 mg, 87%).

$^1$H NMR (600 MHz, (CD3)2SO): δ 3.80 (s, 3H), 6.19 (d, J = 7.8 Hz, 2H), 7.07 (d, J = 8.4 Hz, 2H), 7.46 (d, *J* = 9.0 Hz, 2H), 7.88 (d, *J* = 7.2 Hz, 2H). $^{13}$C NMR (150 MHz, $(CD_3)_2SO$): δ 55.54, 114.86, 117.69, 124.08, 136.09, 140.11, 158.62, 177.10. HRMS (ESI positive mode) *m/z* calculated for $C_{12}H_{11}NNaO_2$ $[M+Na]^+$: 224.0682, found: 224.0679. Mp = 182.0–183.4 °C.

**1-(4-Methoxyphenyl)pyridine-4(1*H*)-thione (2b)**

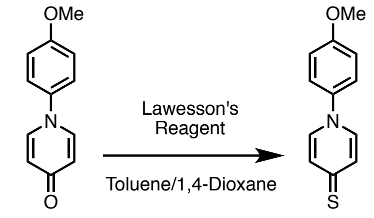


**2a** **2b**

A solution of **2a** (500 mg, 2.48 mmol) and Lawesson's reagent (1.06 g, 2.61 mmol, 1.05 equiv.) in toluene (20 mL) and 1,4-dioxane (2.0 mL) was stirred at refluxing temperature for 4 h. After cooling back to room temperature, ether (20 mL) was added to the mixture. The formed yellow solid was filtered. The obtained solid was subjected to column chromatography on silica gel (eluent: $CH_2Cl_2$/MeOH = 95/5 to 50/50; *v/v*) to give **2b** as a yellow solid (252 mg, 47%).

$^1$H NMR (600 MHz, (CD3)2SO): δ 3.81 (s, 3H), 7.11 (d, J = 8.4 Hz, 2H), 7.24 (d, J = 6.6 Hz, 2H), 7.55 (d, *J* = 9.0 Hz, 2H), 7.83 (d, *J* = 6.6 Hz, 2H). $^{13}$C NMR (150 MHz, $(CD_3)_2SO$): δ 55.64, 114.99, 124.23, 130.14, 135.05, 135.63, 159.29, 190.42. HRMS (ESI positive mode) *m/z* calculated for $C_{12}H_{11}NNaOS$ $[M+Na]^+$: 240.0454, found: 240.0450. Mp = 190.9–192.1 ℃.

**1-(4-Methoxyphenyl)-4-(methylthio)pyridin-1-ium 4-methylbenzenesulfonate (2c)**

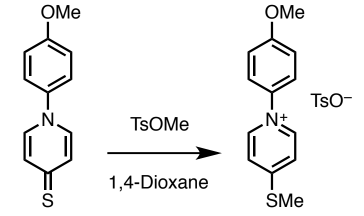


**2b** **2c**

A solution of **2b** (230 mg, 1.06 mmol) and methyl *p*-toluenesulfonate (216 mg, 1.16 mmol, 1.10 equiv.) in 1,4-dioxane (10 mL) was stirred at refluxing temperature for 2 h. After cooling back to room temperature, ether (30 mL) were added to form the precipitation which was then filtered, collected, and dried to give **2c** as a white solid (420 mg, 98%).

$^1$H NMR (600 MHz, $(CD_3)_2SO$): δ 2.73 (s, 3H), 3.22 (s, 3H), 4.32 (s, 3H), 7.55 (d, *J* = 7.8 Hz, 2H), 7.68 (d, *J* = 8.4 Hz, 2H), 7.92 (d, *J* = 7.8 Hz, 2H), 8.20 (d, *J* = 8.4 Hz, 2H), 8.47 (d, *J* = 6.6 Hz, 2H), 9.38 (d, *J* = 6.0 Hz, 2H). $^{13}$C NMR (150 MHz, $(CD_3)_2SO$): δ 14.17, 20.73, 55.85, 115.07, 122.32, 125.45, 125.67, 127.97, 135.16, 137.46, 141.92, 145.85, 160.68, 164.25. HRMS (ESI positive mode) *m/z* calculated for: $C_{13}H_{14}NOS$ $[M]^+$: 232.0791, found: 232.0787. Mp = 155.0 –156.0 ℃.

**1-(4-Methoxyphenyl)-4-((1-(4-methoxyphenyl)pyridin-4(1*H*)-ylidene)methyl)pyridin-1-ium 4-methylbenzenesulfonate (PC2)**

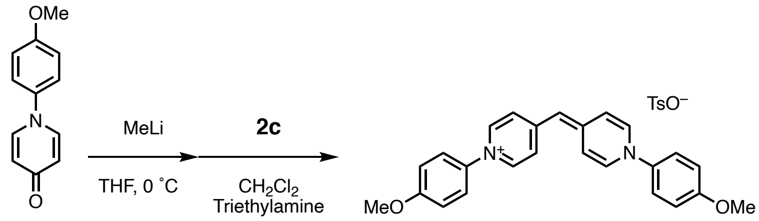


**2a** **PC2**

A solution of **2a** (200 mg, 0.99 mmol) in THF (10 mL) was cooled to 0 ℃ by ice bath under nitrogen atmosphere. 3 M MeLi solution in diethoxymethane (0.66 ml, 2.0 mmol, 2.0 equiv.) was slowly added dropwise for 5 min. The ice bath was removed and the reaction mixture was warmed to room temperature gradually. After additional stirring for 20 min, a solution of **2c** (400 mg, 0.99 mmol) and triethylamine (2 mL) in $CH_2Cl_2$ (10 mL) was added using a syringe at 0 °C. The reaction solution was refluxed at 70 ℃ for 24 h under nitrogen atmosphere. After cooling back to room temperature, the solvent was removed *in vacuo*. Then column chromatography on

silica gel (eluent: $CH_2Cl_2$/MeOH = 95/5 to 80/20; *v/v*) was performed for purification. The obtained red solid was suspended to minimum amount of acetone and sonicated for 5 min and then ether was further added. The solid was filtered to give **PC2** as a red powder (153 mg, 28%).

$^1H$ NMR (600 MHz, $(CD_3)_2SO$): δ 2.27 (s, 3H), 3.82 (s, 6H), 5.75 (s, 1H), 7.09 (d, $J$ = 7.2 Hz, 2H), 7.14 (d, $J$ = 7.2 Hz, 4H), 7.18–7.34 (br, 4H), 7.47 (d, $J$ = 6.6 Hz, 2H), 7.58 (d, $J$ = 7.8 Hz, 4H), 8.08 (d, $J$ = 6.6 Hz, 4H). $^{13}C$ NMR (150 MHz, $(CD_3)_2SO$): δ 20.75, 55.69, 100.36, 115.08, 124.21, 125.48, 128.00, 135.47, 137.50, 138.49, 145.85, 149.24, 159.35. HRMS (ESI positive mode) *m/z* calculated for $C_{25}H_{23}N_2O_2$ $[M]^+$: 383.1754, found: 383.1745. Mp = 196.0–196.8 ℃.

**1-(4-(Dimethylamino)phenyl)pyridin-4(1*H*)-one (3a)**

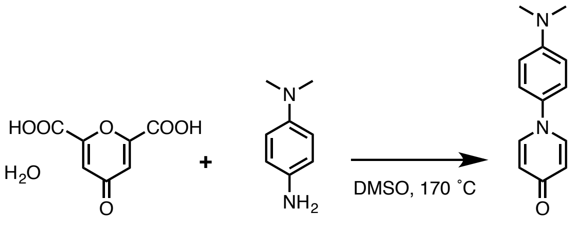


**3a**

Chelidonic acid monohydrate (1.00 g, 4.95 mmol) and *N,N*-dimethyl-1,4-phenylenediamine (741 mg, 5.44 mmol, 1.10 equiv.) were dissolved in DMSO (15 mL). The mixture was stirred at 170 ℃ under open air for 2 h. After cooling back to room temperature, DMSO was removed *in vacuo*. The dark residue was subjected to column chromatography on silica gel (eluent: $CH_2Cl_2$/MeOH = 95/5 to 80/20; *v/v*). Recrystallization from methanol and ether gave **3a** as pale yellow solid (792 mg, 75%).

$^1H$ NMR (400 MHz, $(CD_3)_2SO$): δ 2.93 (s, 6 H), 6.19 (d, $J$ = 8.0 Hz, 2H), 6.80 (d, $J$ = 9.2 Hz, 2H), 7.31 (d, $J$ = 9.2 Hz, 2H), 7.85 (d, $J$ = 7.6 Hz, 2H). $^{13}C$ NMR (150 MHz, $(CD_3)_2SO$): δ 40.10, 112.57, 117.53, 123.43, 132.25, 140.35, 149.82, 176.86. HRMS (ESI positive mode) *m/z* calculated for $C_{13}H_{14}N_2NaO$ $[M+Na]^+$: 237.0998, found: 237.0994. Mp = 181.5–182.2 °C.

**1-(4-(Dimethylamino)phenyl)pyridine-4(1*H*)-thione (3b)**

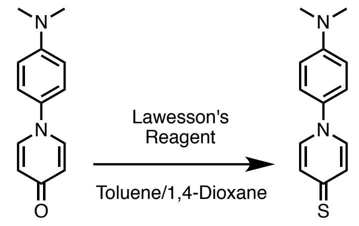


**3a** **3b**

A solution of **3a** (600 mg, 2.80 mmol) and Lawesson's reagent (1.19 g, 2.94 mmol, 1.05 equiv.) in toluene (25 mL) and 1,4-dioxane (2.5 mL) was stirred at refluxing temperature for 3 h. After cooling back to room temperature, ether (30 mL) was added to form the precipitation that was then filtered. The solid was subjected to column chromatography on silica gel (eluent: $CH_2Cl_2$/MeOH = 99/1 to 60/40; *v/v*). Recrystallization from methanol and ether gave **3b** as a yellow solid (347 mg, 54%).

$^1H$ NMR (600 MHz, $(CD_3)_2SO$): δ 2.95 (s, 6H), 6.82 (d, $J$ = 9.0 Hz, 2H), 7.23 (d, $J$ = 6.6 Hz, 2H), 7.39 (d, $J$ = 9.0 Hz, 2H), 7.80 (d, $J$ = 7.2 Hz, 2H). $^{13}C$ NMR (150 MHz, $(CD_3)_2SO$): δ 40.02, 112.47, 123.35, 130.19, 131.63, 135.03, 150.26, 189.49. HRMS (ESI positive mode) *m/z* calculated for $C_{13}H_{14}N_2NaS$ $[M+Na]^+$: 253.0770, found: 253.0768. Mp = 268.0–269.6 °C.

**1-(4-(Dimethylamino)phenyl)-4-(methylthio)pyridin-1-ium 4-methylbenzenesulfonate (3c)**

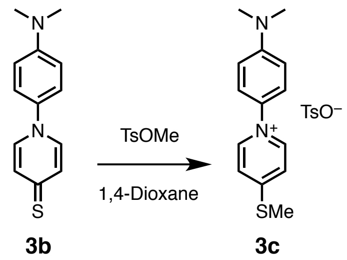

A solution of **3b** (300 mg, 1.30 mmol) and methyl *p*-toluenesulfonate (245 mg, 1.32 mmol, 1.01 equiv.) in 1,4-dioxane (20 mL) was stirred at refluxing temperature for 2 h. After cooling back to room temperature, ether (30 mL) was added to form the precipitation. The resulting solid were collected and dried to give **3c** as a yellow solid (540 mg, 99%).

$^1$H NMR (600 MHz, $(CD_3)_2SO$): δ 2.27 (s, 3H), 2.75 (s, 3H), 3.00 (s, 6H), 6.89 (d, $J$ = 9.0 Hz, 2H), 7.09 (d, $J$ = 7.8 Hz, 2H), 7.46 (d, $J$ = 7.2 Hz, 2H), 7.58 (d, $J$ = 9.0 Hz, 2H), 7.96 (d, $J$ = 6.6 Hz, 2H), 8.87 (d, $J$ = 6.0 Hz, 2H). $^{13}$C NMR (150 MHz, $(CD_3)_2SO$): δ 14.12, 20.74, 112.17, 122.40, 124.53, 125.46, 127.99, 130.82, 137.48, 137.48, 141.39, 145.85, 151.31, 162.92. HRMS (ESI positive mode) *m/z* calculated for $C_{14}H_{17}N_2S$ $[M]^+$: 245.1107, found: 245.1107. Mp = 112.3–113.7 ℃.

**1-(4-(Dimethylamino)phenyl)-4-((1-(4-(dimethylamino)phenyl)pyridin-4(1*H*)-ylidene)methyl)pyridin-1-ium 4-methylbenzenesulfonate (PC3)**

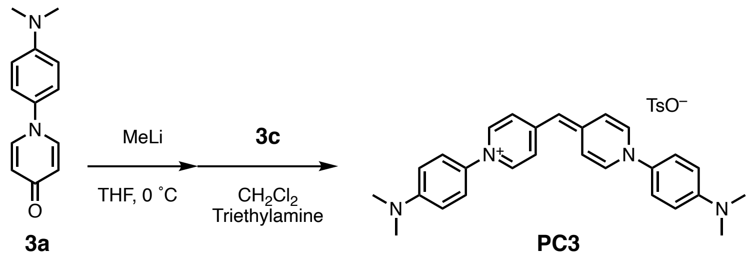

A solution of **3a** (250 mg, 1.17 mmol) in THF (20 mL) was cooled to 0 ℃ by ice bath under nitrogen atmosphere. 3 M MeLi solution in diethoxymethane (0.78 mL, 2.3 mmol, 2.0 equiv.) was slowly added dropwise using a syringe. The ice bath was removed and worm to room temperature gradually. After additional stirring for 20 min, a solution of crude **3c** (486 mg, 1.17 mmol) and triethylamine (4 mL) in $CH_2Cl_2$ (20 mL) was added using a syringe at 0 °C. The mixture was stirred at 70 ℃ for 24 h under nitrogen atmosphere. After cooling back to room temperature, the solvent was removed *in vacuo*. The resulting black solid was subjected to column chromatography on silica gel (eluent: $CH_2Cl_2$/MeOH = 99/1 to 85/15; *v/v*) for purification. The obtained purple solid was suspended to minimum amount of acetone and sonicated for 5 min and then ether was further added. The obtained solid was filtered to give **PC3** as a purple powder (227 mg, 34%).

$^1$H NMR (400 MHz, $(CD_3)_2SO$): δ 2.27 (s, 3H), 2.96 (s, 12H), 5.70 (s, 1H), 6.84 (d, $J$ = 9.2 Hz, 4H), 7.10 (d, $J$ = 7.6 Hz, 2H), 7.04–7.36 (br, 4H), 7.43 (d, $J$ = 9.2 Hz, 4H), 7.46 (d, $J$ = 8.4 Hz, 2H), 8.04 (d, $J$ = 6.8 Hz, 4H). $^{13}$C NMR (150 MHz, $(CD_3)_2SO$): δ 11.80, 31.04, 92.12, 104.40, 114.84, 117.48, 120.28, 123.80, 129.64, 132.10, 134.15, 141.60, 142.73. HRMS (ESI positive mode) *m/z* calculated for $C_{27}H_{29}N_4$ $[M]^+$: 409.2387, found: 409.2381. Mp = 194.8–195.7 ℃.

**1-(4-(Diethylamino)phenyl)pyridin-4(1*H*)-one (4a)**

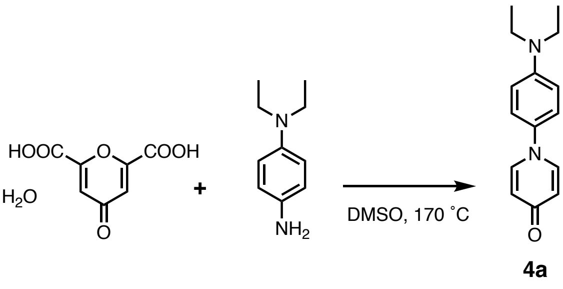

Chelidonic acid monohydrate (1.00 g, 4.95 mmol) and *N*,*N*-diethyl-1,4-phenylenediamine (894 mg, 5.44 mmol, 1.10 equiv.) were dissolved in DMSO (15 mL). The mixture was stirred at 170 °C under open air for 2 h. After cooling back to room temperature, DMSO was removed *in vacuo*. The dark residues were subjected to column chromatography on silica gel (eluent: $CH_2Cl_2$/MeOH = 95/5 to 5/1; *v/v*) for purification. Recrystallization from methanol and ether gave **4a** as a white solid (917 mg, 77%).

$^1$H NMR (600 MHz, $(CD_3)_2SO$): δ 1.08 (t, *J* = 7.2 Hz, 6H), 3.36 (q, *J* = 6.6 Hz, 4H), 6.16 (d, *J* = 7.8 Hz, 2H), 6.72 (d, *J* = 9.0 Hz, 2H), 7.26 (d, *J* = 8.4 Hz, 2H), 7.81 (d, *J* = 7.8 Hz, 2H). $^{13}$C NMR (150 MHz, $(CD_3)_2SO$): δ 12.29, 43.77, 111.64, 117.58, 123.81, 131.32, 140.24, 146.84, 176.96. HRMS (ESI positive mode) *m/z* calculated. for $C_{15}H_{18}N_2NaO$ $[M+Na]^+$: 265.1311, found: 265.1310. Mp = 114.7–116.3 °C.

**1-(4-(Diethylamino)phenyl)pyridine-4(1*H*)-thione (4b)**

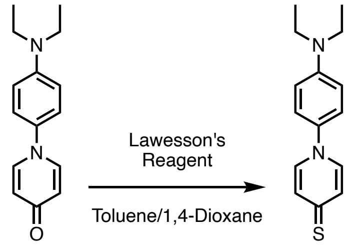


**4a** **4b**

A solution of **4a** (600 mg, 2.48 mmol) and Lawesson's reagent (1.05 g, 2.60 mmol, 1.05 equiv.) in toluene (25 mL) and 1,4-dioxane (2.5 mL) was stirred at refluxing temperature for 3 h. After cooling back to room temperature, ether (20 mL) was added and the formed solid was filtered. The collected solid was subjected to column chromatography on silica gel (eluent: $CH_2Cl_2$/MeOH = 99/1 to 80/20; *v/v*) to give **4b** as a yellow solid (427 mg, 67%).

$^1$H NMR (400 MHz, $(CD_3)_2SO$): δ 1.09 (t, *J* = 7.0 Hz, 6H), 3.37 (q, *J* = 7.2 Hz, 4H), 6.75 (d, *J* = 9.2 Hz, 2H), 7.22 (d, *J* = 7.2 Hz, 2H), 7.35 (d, *J* = 9.2 Hz, 2H), 7.79 (d, *J* = 6.8 Hz, 2H). $^{13}$C NMR (100 MHz, $(CD_3)_2SO$): δ 12.72, 43.81, 111.58, 123.72, 130.17, 130.69, 135.10, 147.37, 189.19. HRMS (ESI positive mode) *m/z* calculated for $C_{15}H_{18}N_2NaS$ $[M+Na]^+$: 281.1083, found: 281.1080. Mp = 166.2–167.9 °C.

**1-(4-(Diethylamino)phenyl)-4-(methylthio)pyridin-1-ium 4-methylbenzenesulfonate (4c)**

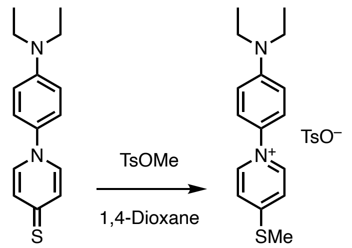


**4b** **4c**

A solution of **4b** (400 mg, 1.55 mmol) and methyl *p*-toluenesulfonate (292 mg, 1.57 mmol, 1.01 equiv.) in 1,4-dioxane (20 mL) was stirred at refluxing temperature for 2 h. After cooling back to room temperature, ether (30 mL) was added to form the precipitation. The resulting solid was filtered, collected and dried to give **4c** as a yellow solid (681 mg, 99%).

$^1$H NMR (600 MHz, $(CD_3)_2SO$): δ 1.11 (t, *J* = 7.2 Hz, 6H), 2.27 (s, 3H), 2.75 (s, 3H), 3.42 (q, *J* = 7.0 Hz, 4H), 6.84 (d, *J* = 9.6 Hz, 2H), 7.09 (d, *J* = 7.8 Hz, 2H), 7.46 (d, *J* = 7.8 Hz, 2H), 7.54 (d, *J* = 9.0 Hz, 2H), 7.96 (d, *J* = 7.8 Hz, 2H), 8.86 (d, *J* = 7.2 Hz, 2H). $^{13}$C NMR (150 MHz, $(CD_3)_2SO$): δ 12.21, 14.10, 20.73, 43.90, 111.42, 122.39, 124.82, 125.46, 127.98, 129.95, 137.48, 141.29, 145.84, 148.53, 162.68. HRMS (ESI positive mode) *m/z* calculated for $C_{16}H_{21}N_2S$ $[M]^+$: 273.1420, found: 273.1419. Mp = 140.5–141.6 °C.

**1-(4-(Diethylamino)phenyl)-4-((1-(4-(diethylamino)phenyl)pyridin-4(1*H*)-ylidene)methyl)pyridin-1-ium 4-methylbenzenesulfonate (PC4)**

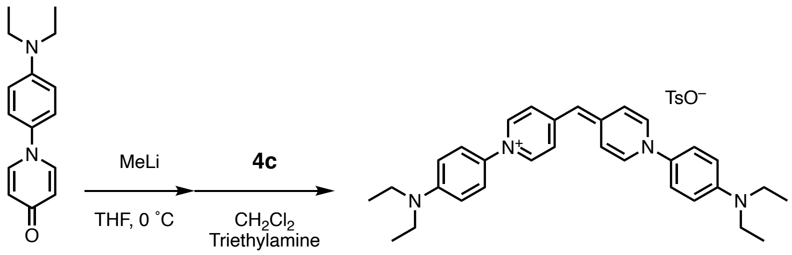


**4a** **PC4**

A solution of **4a** (200 mg, 0.83 mmol) in THF (15 mL) was cooled to 0 ℃ by ice bath under nitrogen atmosphere. 3 M MeLi solution in diethoxymethane (0.55 mL, 1.7 mmol, 2.0 equiv.) was slowly added dropwise using a syringe. The ice bath was removed and the reaction solution was warmed to room temperature gradually. After additional stirring for 20 min, a solution of **4c** (385 mg, 0.87 mmol, 1.1 equiv.) and triethylamine (2 mL) in $CH_2Cl_2$ (15 mL) was added using a syringe at 0 °C. The mixture was stirred at 70 ℃ for 24 h under nitrogen atmosphere. After cooling back to room temperature, the solvent was removed *in vacuo*. The resulting dark red solid was subjected to column chromatography on silica gel (eluent: $CH_2Cl_2$/MeOH = 99/1 to 85/15; *v/v*) to give **PC4** as a purple solid (53 mg, 10%).

$^1$H NMR (400 MHz, $(CD_3)_2SO$): δ 1.10 (t, $J$ = 7.2 Hz, 12H), 2.27 (s, 3H), 3.34–3.42 (m, overlapped with $H_2O$ peak, 8H), 5.68 (s, 1H), 6.77 (d, $J$ = 9.2 Hz, 4H), 7.10 (d, $J$ = 8.0 Hz, 2H), 7.06–7.32 (br, 4H), 7.37 (d, $J$ = 9.2 Hz, 4H), 7.47 (d, $J$ = 8.0 Hz, 2H), 8.01 (d, $J$ = 7.2 Hz, 4H). $^{13}$C NMR (100 MHz, $(CD_3)_2SO$): δ 12.32, 20.80, 43.86, 99.80, 111.67, 123.60, 125.52, 128.06, 130.50, 137.56, 138.13 (br), 145.82, 147.33, 148.55. HRMS (ESI positive mode) *m/z* calculated for $C_{31}H_{37}N_4$ $[M]^+$: 465.3013, found: 465.3007. Mp = 64.0–65.5℃.

**Supplementary Note 2**: Synthesis of **PC5**, **PC6**, **PC7**, and **PC8**.

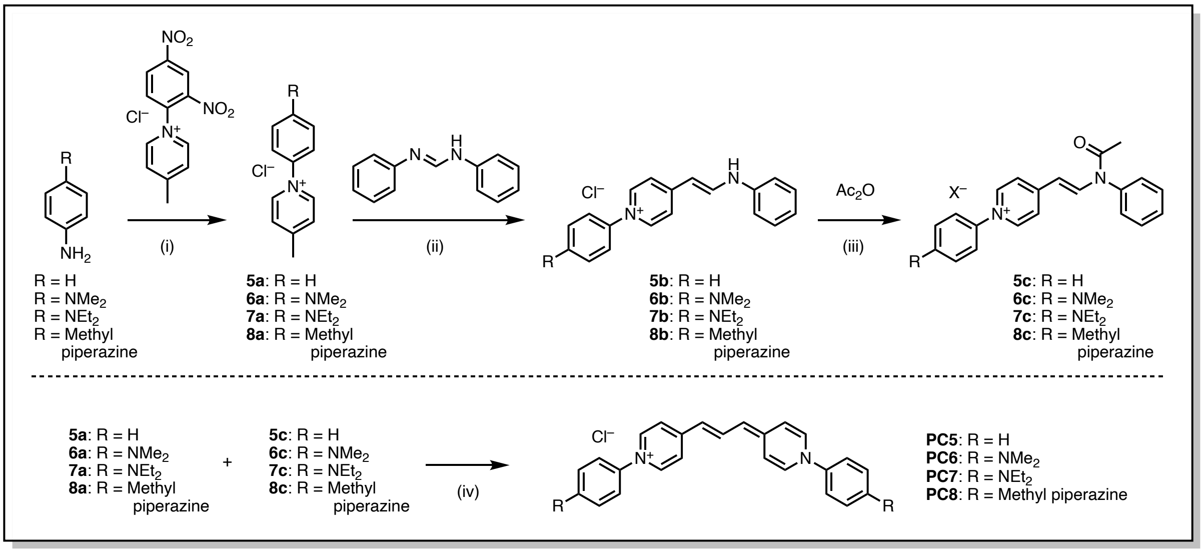


Scheme S2. (i) 1-(2,4-dinitrophenyl)-4-methylpyridin-1-ium chloride,[*] ethanol, reflux, 2.5–12 h; (ii) *N*,*N*'-diphenylformamidine, acetone/ethanol (= 1/1; *v/v*), reflux, 6–12 h; (iii) acetone/acetic anhydride (= 1/1; *v/v*), 60 °C, 4 h, $X^-$ = $Cl^-$ or $AcO^-$; (iv) $NEt_3$, $CH_2Cl_2$, reflux, 1 day.

**1-Phenyl-4-(2-(phenylamino)vinyl)pyridin-1-ium chloride (5b)**

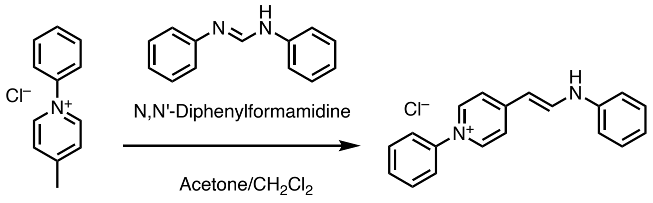


**5a** **5b**

A solution of 4-methyl-1-phenylpyridin-1-ium chloride (**5a**, 150 mg, 0.73 mmol) and *N*,*N'*-diphenylformamidine (286 mg, 1.46 mmol, 2.0 equiv.) in acetone (20 mL) and $CH_2Cl_2$ (20 mL) was stirred at reflux for 12 h. After cooling back to room temperature, the solvent was removed *in vacuo*. The resulting solid was subjected to column chromatography on silica gel (eluent: $CH_2Cl_2$/MeOH = 95/5 to 80/20; *v/v*) to give **5b** as a yellow powder (136 mg, 60%). The crude product was used to next reaction without further purification.

**1-phenyl-4-(2-(N-phenylacetamido)vinyl)pyridin-1-ium acetate (5c)**

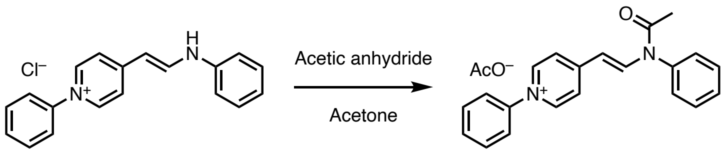


**5b** **5c**

To a solution of **5b** (120 mg, 0.39 mmol) in acetone (20 mL) was added acetic anhydride (20 mL), and the resultant mixture was stirred at 60 °C for 4 h. After cooling back to room temperature, acetone and acetic anhydride were removed *in vacuo*. The resulting solid was purified by column chromatography on silica gel (eluent: $CH_2Cl_2$/MeOH = 95/5 to 80/20; *v/v*) to give **5c** as a yellow powder (116 mg, 85%).

$^1$H NMR (400 MHz, $(CD_3)_2SO$): δ 1.88 (s, 3H), 2.05 (s, 3H), 5.53 (d, $J$ = 14.4 Hz, 1H), 7.44 (d, $J$ = 7.6 Hz, 2H), 7.54–7.74 (m, 6H), 7.76–7.84 (m, 4H), 8.16 (d, $J$ = 7.2 Hz, 2H), 8.87 (d, $J$ = 14.8 Hz, 1H), 8.96 (d, $J$ = 6.8 Hz, 2H). $^{13}$C NMR (100 MHz, $(CD_3)_2SO$): δ 21.36, 23.23, 106.32, 122.33, 124.23, 128.56, 129.54, 130.10, 130.42, 130.62, 137.87, 140.51, 142.17, 143.10, 154.71, 169.78, 172.35. HRMS (ESI positive mode) *m/z* calculated for $C_{21}H_{19}N_2O$ $[M]^+$: 315.1492, found: 315.1486. Mp = 151.9–153.2 °C.

**1-Phenyl-4-(3-(1-phenylpyridin-4(1*H*)-ylidene)prop-1-en-1-yl)pyridin-1-ium chloride (PC5)**

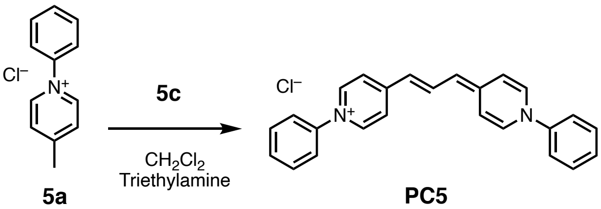


**5a** **PC5**

To a solution of **5a** (55 mg, 267 mmol) and **5c** (103 mg, 0.29 mmol, 1.1 equiv..) in $CH_2Cl_2$ (10 mL) was added triethylamine (2.0 mL) using a syringe under nitrogen atmosphere. The mixture was stirred at refluxing temperature for 1 day. After cooling back to room temperature, the solvent was removed *in vacuo*. The crude product was purified by column chromatography on silica gel (eluent: $CH_2Cl_2$/MeOH = 95/5 to 80/20; *v/v*). The blue fractions were collected and concentrated to give solid which was then washed by acetone once to give **PC5** as a blue solid (81.4 mg, 79%).

$^1$H NMR (600 MHz, $(CD_3)_2SO$): δ 6.00 (d, $J$ = 13.8 Hz, 2H), 7.32–7.36 (m, 4H), 7.52 (t, $J$ = 6.6 Hz, 2H), 7.56–7.66 (m, 8H), 8.03 (d, $J$ = 7.2 Hz, 4H), 8.27 (t, $J$ = 13.8 Hz, 1H). $^{13}$C NMR (100 MHz, $(CD_3)_2SO$): δ 111.10, 122.63, 128.59, 130.04, 137.85, 141.94, 142.25, 150.27. HRMS (ESI positive mode) *m/z* calculated for $C_{25}H_{21}N_2$ $[M]^+$: 349.1699, found: 349.1699. Mp = 194.2–195.0 °C.

**1-(4-(Dimethylamino)phenyl)-4-methylpyridin-1-ium chloride (6a)**

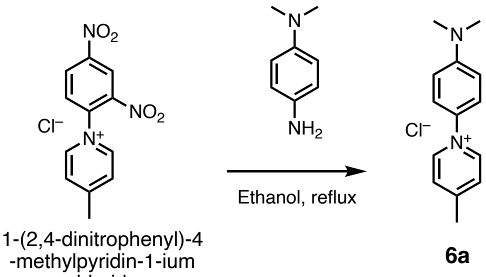


1-(2,4-dinitrophenyl)-4
-methylpyridin-1-ium
chloride

**6a**

To a solution of 1-(2,4-dinitrophenyl)-4-methylpyridin-1-ium chloride (*) (2.0 g, 6.76 mmol) in ethanol (50 mL) was added a solution of *N*,*N*-dimethyl-1,4-phenylenediamine (1.11 g, 8.12 mmol, 1.2 equiv.) in ethanol (10 mL) using a syringe at room temperature. Then the mixture was stirred at refluxing temperature for 4 h. After

cooled back to room temperature, solvent was removed *in vacuo*. Then the resulting dark crude product was purified by column chromatography on silica gel (eluent: $CH_2Cl_2$/MeOH = 9/1 to 7/3; *v/v*) to give **6a** as a yellow solid (1.28 g, 76%).

$^1$H NMR (400 MHz, $(CD_3)_2SO$): δ 2.67 (s, 3H), 3.01 (s, 6H), 6.90 (d, *J* = 9.6 Hz, 2H), 7.63 (d, *J* = 9.6 Hz, 2H), 8.05 (d, *J* = 6.4 Hz, 2H), 9.09 (d, *J* = 6.8 Hz, 2H). $^{13}$C NMR (100 MHz, $(CD_3)_2SO$): δ 21.33, 112.18, 124.72, 128.33, 131.13, 142.98, 151.47, 158.47. HRMS (ESI positive mode) *m/z* calculated for $C_{14}H_{17}N_2$ $[M]^+$: 213.1386, found: 213.1386. Mp = 114.8–115.9 °C.

**1-(4-(Dimethylamino)phenyl)-4-(2-(phenylamino)vinyl)pyridin-1-ium chloride (6b)**

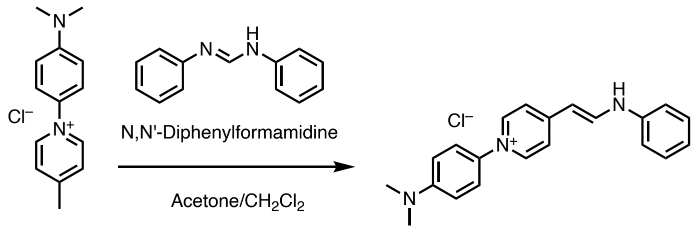


**6a** **6b**

A solution of **6a** (200 mg, 0.80 mmol) and *N,N'*-diphenylformamidine (316 mg, 161 mmol, 2.0 equiv.) in acetone (20 mL) and $CH_2Cl_2$ (20 mL) was stirred at refluxing temperature for 6 h. After cooling back to room temperature, the solvent was removed *in vacuo*. The obtained crude solid was subjected to column chromatography on silica gel (eluent: $CH_2Cl_2$/MeOH = 95/5 to 80/20; *v/v*) to give **6b** as an orange powder (162 mg, 57%).

$^1$H NMR (400 MHz, $(CD_3)_2SO$): δ 2.98 (s, 6H), 6.00 (d, *J* = 13.2 Hz, 1H), 6.87 (d, *J* = 9.6 Hz, 2H), 7.04 (t, *J* = 7.6 Hz, 1H), 7.30–7.44 (m, 4H), 7.53 (d, *J* = 9.2 Hz, 2H), 7.70–8.00 (br, 2H), 8.51 (d, *J* = 7.2 Hz, 2H), 8.64 (d, *J* = 13.2 Hz, 1H), 10.7–11.0 (br, 1H). $^{13}$C NMR (100 MHz, $(CD_3)_2SO$): δ 39.92, 99.09, 112.31, 116.09, 118.79, 122.96, 124.05, 129.50, 131.23, 140.48, 140.58, 143.52, 150.82, 154.50. HRMS (ESI positive mode) *m/z* calculated for $C_{21}H_{22}N_3$ $[M]^+$: 316.1808, found: 316.1808. Mp = 122.8–124.2 °C.

**1-(4-(Dimethylamino)phenyl)-4-(2-(*N*-phenylacetamido)vinyl)pyridin-1-ium chloride (6c)**

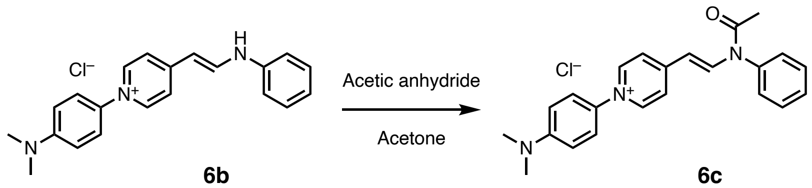


**6b** **6c**

To a solution of **6b** (120 mg, 341 mmol) in acetone (10 mL) was added acetic anhydride (10 mL) at room temperature under nitrogen atmosphere. Then the mixture was stirred at 60 °C for 4 h. After cooling back to room temperature, the solution was removed *in vacuo*. The resulting solid was subjected to column chromatography on silica gel (eluent: $CH_2Cl_2$/MeOH = 95/5 to 80/20; *v/v*) to give **6c** as an orange powder (102 mg, 76%).

$^1$H NMR (400 MHz, $(CD_3)_2SO$): δ 2.03 (s, 3H), 3.00 (s, 6H), 5.49 (d, *J* = 14.8 Hz, 1H), 6.87 (d, *J* = 9.2 Hz, 2H), 7.43 (d, *J* = 7.6 Hz, 2H), 7.52–76.8 (m, 5H), 8.07 (d, *J* = 7.2 Hz, 2H), 8.80 (d, *J* = 14.4 Hz, 1H), 8.87 (d, *J* = 7.2 Hz, 2H). $^{13}$C NMR (100 MHz, $(CD_3)_2SO$): δ 23.25, 106.47, 112.18, 122.42, 124.41, 128.60, 129.52, 130.43, 130.93, 137.96, 139.60, 142.36, 151.26, 153.02, 169.71 (two peaks were missing). HRMS (ESI positive mode) *m/z* calculated for $C_{23}H_{24}N_3O$ $[M]^+$: 358.1914, found: 358.1909. Mp = 109.0–111.1 °C.

**1-(4-(Dimethylamino)phenyl)-4-(3-(1-(4-(dimethylamino)phenyl)pyridin-4(1*H*)-ylidene)prop-1-en-1-yl)pyridin-1-ium chloride (PC6)**

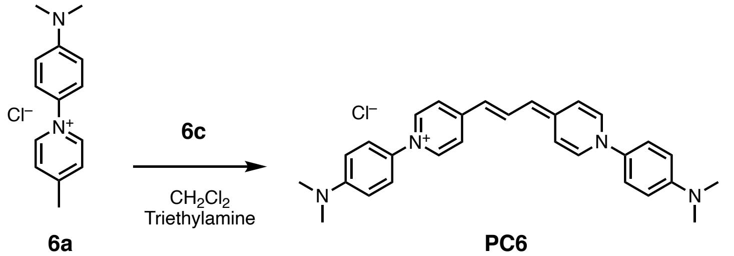


**6a** **PC6**

To a solution of **6a** (50.0 mg, 0.20 mmol) and **6c** (87.4 mg, 0.22 mmol, 1.1 equiv.) in $CH_2Cl_2$ (10 mL) was added triethylamine (2 mL) using a syringe under nitrogen atmosphere. The mixture was stirred at refluxing temperature for 1 day. After cooling back to room temperature, the solvent was removed *in vacuo*. The resulting solid was purified by column chromatography on silica gel (eluent: $CH_2Cl_2$/MeOH = 95/5 to 80/20; *v/v*). The blue fractions were collected and concentrated. The obtained solid was dissolved in acetone to which ether was added to form the precipitation, which was then filtered to give **PC6** as a blue solid (28.8 mg, 30%).

$^1$H NMR (600 MHz, $(CD_3)_2SO$): δ 2.96 (s, 12H), 5.88 (d, $J$ = 13.2 Hz, 2H), 6.83 (d, $J$ = 8.4 Hz, 4H), 7.42 (d, $J$ = 9.0 Hz, 4H), 7.95 (d, $J$ = 7.2 Hz, 4H), 8.23 (t, $J$ = 13.2 Hz, 1H). $^1$H NMR (600 MHz, $(CD_3)_2SO$, 60 °C): δ 2.97 (s, 12H), 5.90 (d, $J$ = 13.2 Hz, 2H), 6.84 (d, $J$ = 8.4 Hz, 4H), 7.24 (br, 4H), 7.40 (d, $J$ = 8.4 Hz, 4H), 7.9 (d, $J$ = 7.2 Hz, 4H), 8.17 (t, $J$ = 13.2 Hz, 1H). $^{13}$C NMR (150 MHz, $(CD_3)_2SO$, 80 °C): δ 109.73, 112.30, 116.38, 122.84, 131.27, 137.37, 140.42, 149.19, 150.05. HRMS (ESI positive mode) *m/z* calculated for $C_{29}H_{31}N_4$ $[M]^+$: 435.2543, found: 435.2552. Mp = 143.6–174.7 ℃.

**1-(4-(Diethylamino)phenyl)-4-methylpyridin-1-ium chloride (7a)**

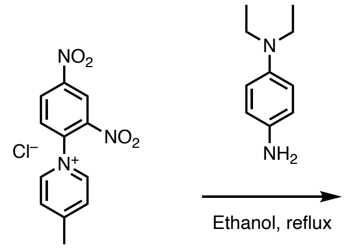


1-(2,4-dinitrophenyl)-4-methylpyridin-1-ium chloride

**7a**

To a solution of 1-(2,4-Dinitrophenyl)-4-methylpyridin-1-ium chloride (5.0 g, 17 mmol) in ethanol (70 mL) was added a solution of *N,N*-diethyl-1,4-phenylenediamine (3.33 g, 20.3 mmol, 1.2 equiv..) in ethanol (20 mL) using a syringe at room temperature. Then the mixture was stirred at refluxing temperature for 12 h. After cooled to room temperature, solvent was removed *in vacuo*. The obtained dark oil was purified by column chromatography on silica gel (eluent: $CH_2Cl_2$/MeOH = 90/10 to 70/30; *v/v*) to give **7a** as a yellow solid (3.86 g, 82%).

$^1$H NMR (400 MHz, $(CD_3)_2SO$): δ 1.11 (t, $J$ = 7.4 Hz, 6H), 2.66 (s, 3H), 3.43 (q, $J$ = 7.1 Hz, 4H), 6.85 (d, $J$ = 9.2 Hz, *2*H), 7.59 (d, $J$ = 9.2 Hz, *2*H), 8.04 (d, $J$ = 7.2 Hz, 2H), 9.08 (d, $J$ = 6.8 Hz, 2H). $^{13}$C NMR (100 MHz, $(CD_3)_2SO$): δ 12.25, 21.31, 43.93, 111.42, 125.03, 128.31, 130.27, 142.87, 148.72, 158.27. HRMS (ESI positive mode) *m/z* calculated for $C_{16}H_{21}N_2$ $[M]^+$: 241.1699, found: 241.1697. Mp = 138.5–139.9 ℃

**1-(4-(Diethylamino)phenyl)-4-(2-(phenylamino)vinyl)pyridin-1-ium chloride (7b)**

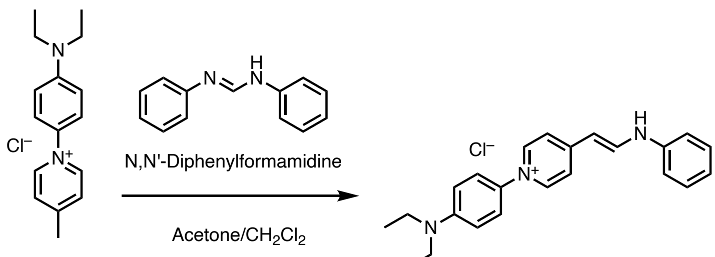


**7a** **7b**

A solution of **7a** (300 mg, 1.08 mmol) and *N,N'*-diphenylformamidine (425 mg, 2.17 mmol, 2.0 equiv.) in acetone (30 mL) and $CH_2Cl_2$ (30 mL) was stirred at refluxing temperature for 6 h. After cooling back to room temperature, the solvent was removed. The resulting red solid was subjected to column chromatography on silica gel (eluent $CH_2Cl_2$/MeOH = 95/5 to 80/20; *v/v*) to give **7b** as a red powder (287 mg, 70%).

$^1$H NMR (400 MHz, $(CD_3)_2SO$): δ 1.11 (t, $J$ = 7.0 Hz, 6H), 3.41 (q, $J$ = 7.2 Hz, 4H), 5.96 (d, $J$ = 13.2 Hz, 1H), 6.80 (d, $J$ = 9.2 Hz, 2H), 7.03 (t, $J$ = 7.8 Hz, 1H), 7.30–7.42 (m, 4H), 7.48 (d, $J$ = 9.2 Hz, 2H), 7.60–7.90 (br, 2H), 8.44 (d, $J$ = 7.2 Hz, 2H), 8.62 (d, $J$ = 12.8 Hz, 1H), 10.5–11.3 (br, 1H). $^{13}$C NMR (100 MHz, $(CD_3)_2SO$): δ 12.27, 43.89, 99.22, 111.51, 116.26, 118.45 (br), 122.91, 124.30, 129.45, 130.37, 140.27, 141.11, 143.95, 147.89, 153.94. HRMS (ESI positive mode) *m/z* calculated for $C_{23}H_{26}N_3$ $[M]^+$: 344.2121, found: 344.2118.

**1-(4-(Diethylamino)phenyl)-4-(2-(*N*-phenylacetamido)vinyl)pyridin-1-ium chloride (7c)**

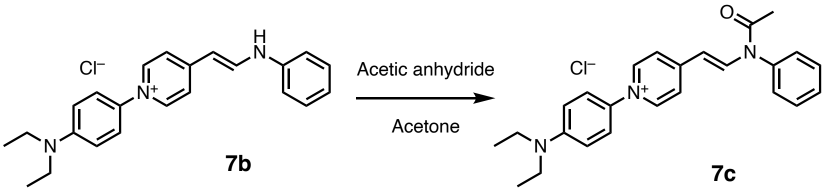


To a solution of **7b** (250 mg, 0.66 mmol) in acetone (10 mL) was added acetic anhydride (20 mL) using a syringe at room temperature under nitrogen atmosphere. The mixed solution was stirred at 60 °C for 4 h under nitrogen atmosphere. Then, the solvent was removed *in vacuo*. The resulting solid was purified by column chromatography on silica gel (eluent: $CH_2Cl_2$/MeOH = 95/5 to 80/20; *v/v*) to give **7c** as a red powder (131 mg, 47%).

$^1$H NMR (400 MHz, $(CD_3)_2SO$): δ 1.10 (t, $J$ = 7.2 Hz, 6H), 2.03 (s, 3H), 3.41 (q, $J$ = 7.2 Hz, 4H), 5.48 (d, $J$ = 14.4 Hz, 1H), 6.82 (d, $J$ = 9.6 Hz, 2H), 7.43 (d, $J$ = 7.2 Hz, 2H), 7.50–7.68 (m, 5H), 8.06 (d, $J$ = 6.8 Hz, 2H), 8.79 (d, $J$ = 14.8 Hz, 1H), 8.84 (d, $J$ = 7.2 Hz, 2H). $^{13}$C NMR (100 MHz, $(CD_3)_2SO$): δ 12.26, 23.24, 43.92, 106.48, 111.44, 122.42, 124.72, 128.60, 129.51, 130.06, 130.42, 137.96, 142.26, 148.51, 152.82, 167.70. One carbon peak was not found. HRMS (ESI positive mode) *m/z* calculated for $C_{25}H_{28}N_3O$ $[M]^+$: 386.2227, found: 386.2225. Mp = 243.3–244.6 °C.

**1-(4-(Diethylamino)phenyl)-4-(3-(1-(4-(diethylamino)phenyl)pyridin-4(1*H*)-ylidene)prop-1-en-1-yl)pyridin-1-ium chloride (PC7)**

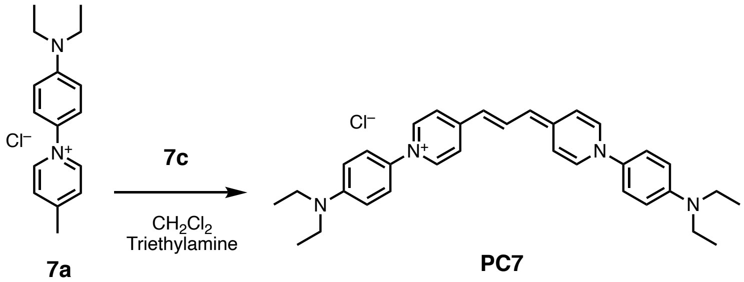


To a solution of **7a** (70 mg, 0.25 mmol) and **7c** (112 mg, 0.27 mmol, 1.05 equiv.) in $CH_2Cl_2$ (10 mL) was added triethylamine (1 mL) using a syringe under nitrogen atmosphere. The mixture was stirred at refluxing temperature for 1 day. After cooling back to room temperature, the solvent was removed *in vacuo*. The resulting dark solid was subjected to column chromatography on silica gel (eluent: $CH_2Cl_2$/MeOH = 95/5 to 80/20; *v/v*). The blue fractions were collected and concentrated. The obtained solid was dissolved in acetone, and ether was added to the solution to form the precipitation, which was then filtered to give **PC7** as a blue solid (32.7 mg, 25%).

$^1$H NMR (600 MHz, $(CD_3)_2SO$): δ 1.10 (t, $J$ = 6.6 Hz, 12H), 3.34–3.42 (m, 8H), 5.86 (d, $J$ = 13.2 Hz, 2H), 6.77 (d, $J$ = 8.4 Hz, 4H), 7.37 (d, $J$ = 9.0 Hz, 4H), 7.95 (distorted s, 4H), 8.22 (t, $J$ = 13.8 Hz, 1H). $^{13}$C NMR (150 MHz, $(CD_3)_2SO$): δ 12.30, 43.86, 109.87, 111.66, 123.55, 130.63, 137.74, 140.72, 147.24, 149.38. One carbon peak was not found. HRMS (ESI positive mode) *m/z* calculated for $C_{33}H_{39}N_4$ $[M]^+$: 491.3169, found: 491.3170. Mp = 155.1–156.5 °C.

**4-Methyl-1-(4-(4-methylpiperazin-1-yl)phenyl)pyridin-1-ium chloride (8a)**

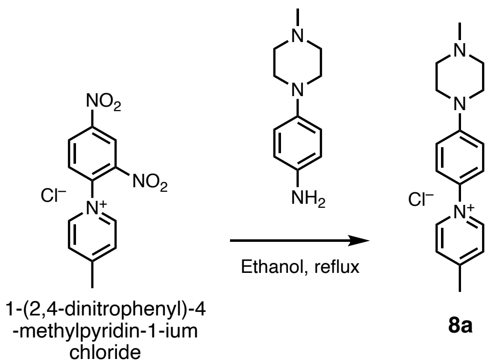

To a solution of 1-(2,4-dinitrophenyl)-4-methylpyridin-1-ium chloride (3.0 g, 10 mmol) in ethanol (50 mL) was added a solution of 4-(4-methylpiperazin-1-yl)aniline (2.04 g, 1.1 mmol, 1.1 equiv.) in ethanol (10 mL) using a syringe at room temperature. The reaction mixture was stirred 2.5 h. After cooling back to room temperature, the solvent was removed *in vacuo*. Then the resulting dark oil was subjected to column chromatography on silica gel (amino silica gel, eluent: $CH_2Cl_2$/MeOH = 100/1 to 95/5; *v/v*) to give **8a** as an orange solid (855 mg, 28%).

$^1$H NMR (400 MHz, $(CD_3)_2SO$): δ 2.22 (s, 3H), 2.38–2.48 (m, 4H), 2.68 (s, 3H), 3.23–3.32 (m, 4H), 7.18 (d, *J* = 8.8 Hz, 2H), 7.66 (d, *J* = 9.2 Hz, 2H), 8.07 (d, *J* = 6.4 Hz, 2H), 9.12 (d, *J* = 5.6 Hz, 2H). $^{13}$C NMR (100 MHz, $(CD_3)_2SO$): δ 21.36, 45.71, 47.15, 54.26, 115.03, 124.84, 128.31, 132.93, 143.12, 152.19, 158.91. HRMS (ESI positive mode) *m/z* calculated for $C_{17}H_{22}N_3$ $[M]^+$: 268.1808, found: 268.1807. Mp = 74.1–75.6 ℃.

**1-(4-(4-Methylpiperazin-1-yl)phenyl)-4-(2-(phenylamino)vinyl)pyridin-1-ium chloride(8b)**

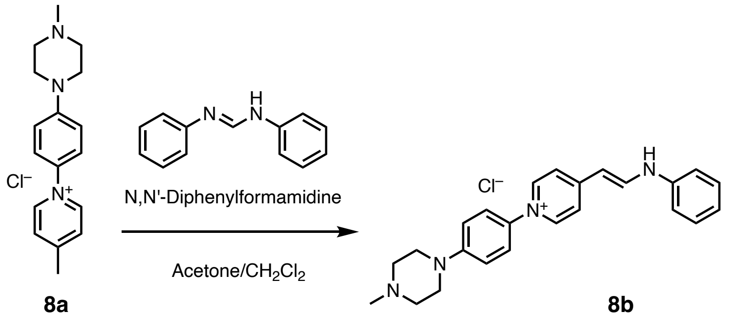


A solution of **8a** (300 mg, 0.99 mmol) and *N,N'*-diphenylformamidine (387 mg, 1.97 mmol, 2.0 equiv.) in acetone (25 mL) and $CH_2Cl_2$ (25 mL) was stirred at refluxing temperature for 12 h. After cooling back to room temperature, the solvent was removed *in vacuo*. The resulting solid was subjected to column chromatography on silica gel (amino silica gel, eluent: $CH_2Cl_2$/MeOH = 100/0 to 90/10; *v/v*) to give **8b** (208 mg, 52%) as an orange powder.

$^1$H NMR (400 MHz, $(CD_3)_2SO$): δ 2.21 (s, 3H), 2.38–2.47 (m, 4H), 3.08–3.20 (m, 4H), 5.35 (d, *J* = 10.0 Hz, 1H), 6.21 (br s, 1H), 6.90–7.10 (m, 6H), 7.20–7.40 (m, 7H), 8.40 (d, *J* = 10.0 Hz, 1H). $^{13}$C NMR (100 MHz, $(CD_3)_2SO$): δ 45.75, 47.92, 54.46, 103.01, 109.26, 114.96, 115.91, 120.59, 121.78, 122.89, 128.81, 133.89 (br), 134.22, 144.32, 149.61, 153.83, 156.04. One extra carbon peak was observed. HRMS (ESI positive mode) *m/z* calculated for $C_{24}H_{27}N_4$ $[M]^+$: 371.2230, found: 371.2225.

**1-(4-(4-methylpiperazin-1-yl)phenyl)-4-(2-(N-phenylacetamido)vinyl)pyridin-1-ium chloride (8c)**

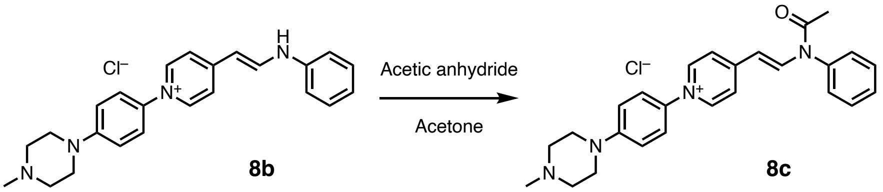


To a solution of **8b** (150 mg, 0.37 mmol**)** in acetone (20 mL) was added acetic anhydride (20 mL) using a syringe at room temperature under nitrogen atmosphere. Then the mixture was stirred at 60 °C for 4 h. After cooling back to room temperature, the solution was removed *in vacuo*. The resulting solid was purified by column chromatography on silica gel (amino silica gel, eluent: $CH_2Cl_2$/MeOH = 100/0 to 90/10; *v/v*) to give **8c** as a red powder (128 mg, 77%). The obtained product was used to the next reaction without further purification.

**1-(4-(4-methylpiperazin-1-yl)phenyl)-4-(3-(1-(4-(4-methylpiperazin-1-yl)phenyl)pyridin-4(1*H*)-ylidene)prop-1-en-1-yl)pyridin-1-ium chloride (PC8)**

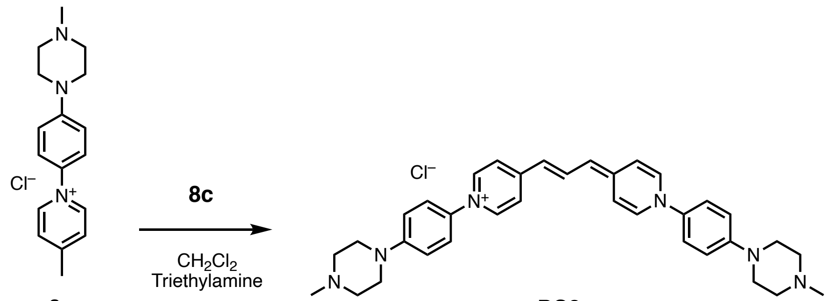


**8a** **PC8**

To a solution of **8a** (70 mg, 0.23 mmol) and **8c** (113 mg, 0.25 mmol, 1.1 equiv.) in $CH_2Cl_2$ (10 mL) was added triethylamine (1 mL) using a syringe under nitrogen atmosphere. The reaction mixture was stirred at refluxing temperature for 1 day. After cooling back to room temperature, the solvent was removed *in vacuo*. The resulting solid was subjected to column chromatography on amino silica gel (amino silica gel, eluent: $CH_2Cl_2$/MeOH = 100/0 to 90/10; *v/v*). The blue fractions were collected and concentrated. Then the obtained solid was recrystallized from methanol to give **PC8** as a glossy dark green powder (92 mg, 69%).

$^1$H NMR (400 MHz, $(CD_3)_2SO$): δ 2.22 (s, 6H), 2.36–2.47 (m, 8H), 3.14–3.27 (m, 8H), 5.89 (d, $J$ = 13.6 Hz, 2H), 7.08 (d, $J$ = 9.2 Hz, 4H), 7.45 (d, $J$ = 8.8 Hz, 4H), 7.99 (d, $J$ = 7.6 Hz, 4H), 8.26 (t, $J$ = 13.6 Hz, 1H). $^{13}$C NMR (100 MHz, $(CD_3)_2SO$): δ 45.23, 47.32, 54.02, 110.00, 115.21, 116.41, 122.86, 133.07, 137.34, 140.67, 149.34, 150.53. HRMS (ESI positive mode) *m/z* calculated for $C_{35}H_{41}N_6$ $[M]^+$: 545.3387, found: 545.3389. Mp = 65.5–67.0 ℃.

**Supplementary Note 3**: Synthesis of **PC9**.

**1-(4-(Diethylamino)phenyl)-4-((1-(4-(4-methylpiperazin-1-yl)phenyl)pyridin-4(1*H*)-ylidene)methyl)pyridin-1-ium 4-methylbenzenesulfonate (PC9)**

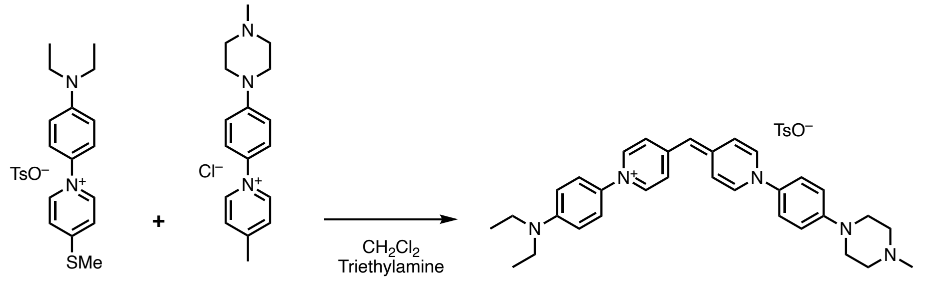


**4c** **8a** **PC9**

**4c** (100 mg, 0.224 mmol) and **8a** (72 mg, 0.24 mmol, 1.05 equiv) were dissolved in $CH_2Cl_2$ (10 mL) and then of triethylamine (1 mL) were added using a syringe under nitrogen atmosphere. The reaction solution was refluxed for 1 day under nitrogen atmosphere. After removal of reaction solution using a rotary evaporator, the crude product was purified by column chromatography on silica gel (eluent: $CH_2Cl_2$/MeOH = 100:0 to 80:20) to give **PC9** as a moisty sensitive green solid (96 mg, 64%).

$^1$H NMR (600 MHz, $(CD_3)_2SO$): δ 1.10 (t, $J$ = 7.2 Hz, 6H), 2.22 (s, 3H), 2.27 (s, 3H), 2.45 (t, $J$ = 4.8 Hz, 4H), 3.22 (t, $J$ = 4.8 Hz, 4H), 3.39 (q, $J$ = 7.2 Hz, 4H), 5.70 (s, 1H), 6.78 (d, $J$ = 9.0 Hz, 2H), 7.00–7.34 (br. 4H), 7.06–7.14 (m, 4H), 7.39 (d, $J$ = 9.0 Hz, 2H), 7.45 (t, $J$ = 9.0 Hz, 4H), 8.02 (d, $J$ = 7.2 Hz, 2H), 8.05 (d, $J$ = 7.2 Hz, 2H). $^{13}$C NMR (150 MHz, $(CD_3)_2SO$): δ 12.27, 20.73, 43.82, 45.71, 47.54, 54.36, 99.93, 111.66, 115.55, 123.20, 123.66, 125.46, 127.97, 130.47, 133.33, 137.44, 137.98, 138.35, 145.88, 147.41, 148.50, 148.89, 150.82. (ESI+) *m/z* calcd for $C_{35}H_{41}N_6^+$ $[M]^+$: 492.3122, found: 492.3123. Mp =65.5-67.0℃

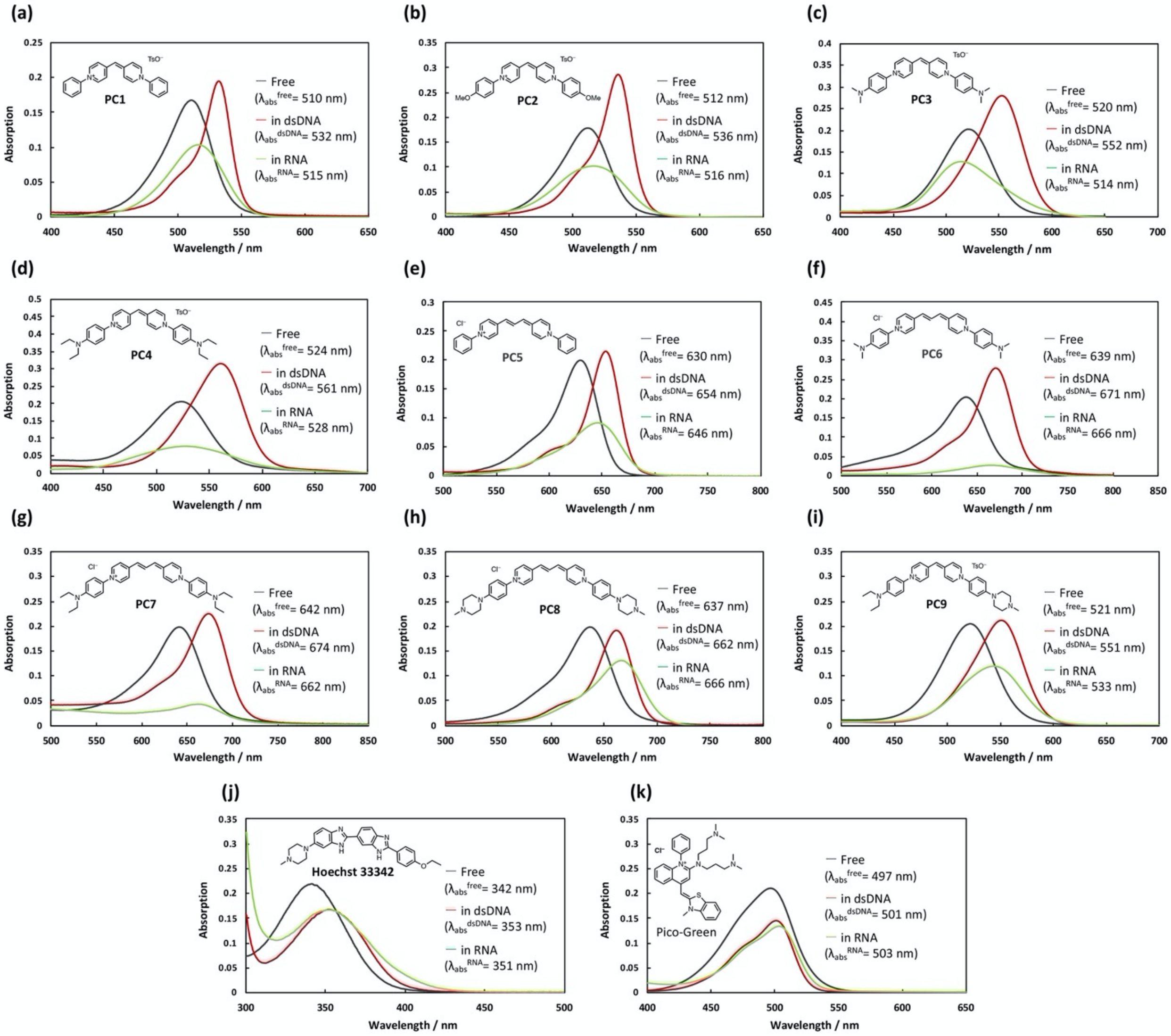


**Supplementary Fig. 1. Absorption spectra of N-aryl PC derivatives and two popular commercialized DNA probes in EDTA solution upon mixing excess of dsDNA and RNA.** Black lines, red lines, and green lines represent free state, DNA-complexed state, and RNA-complexed state absorption, respectively.

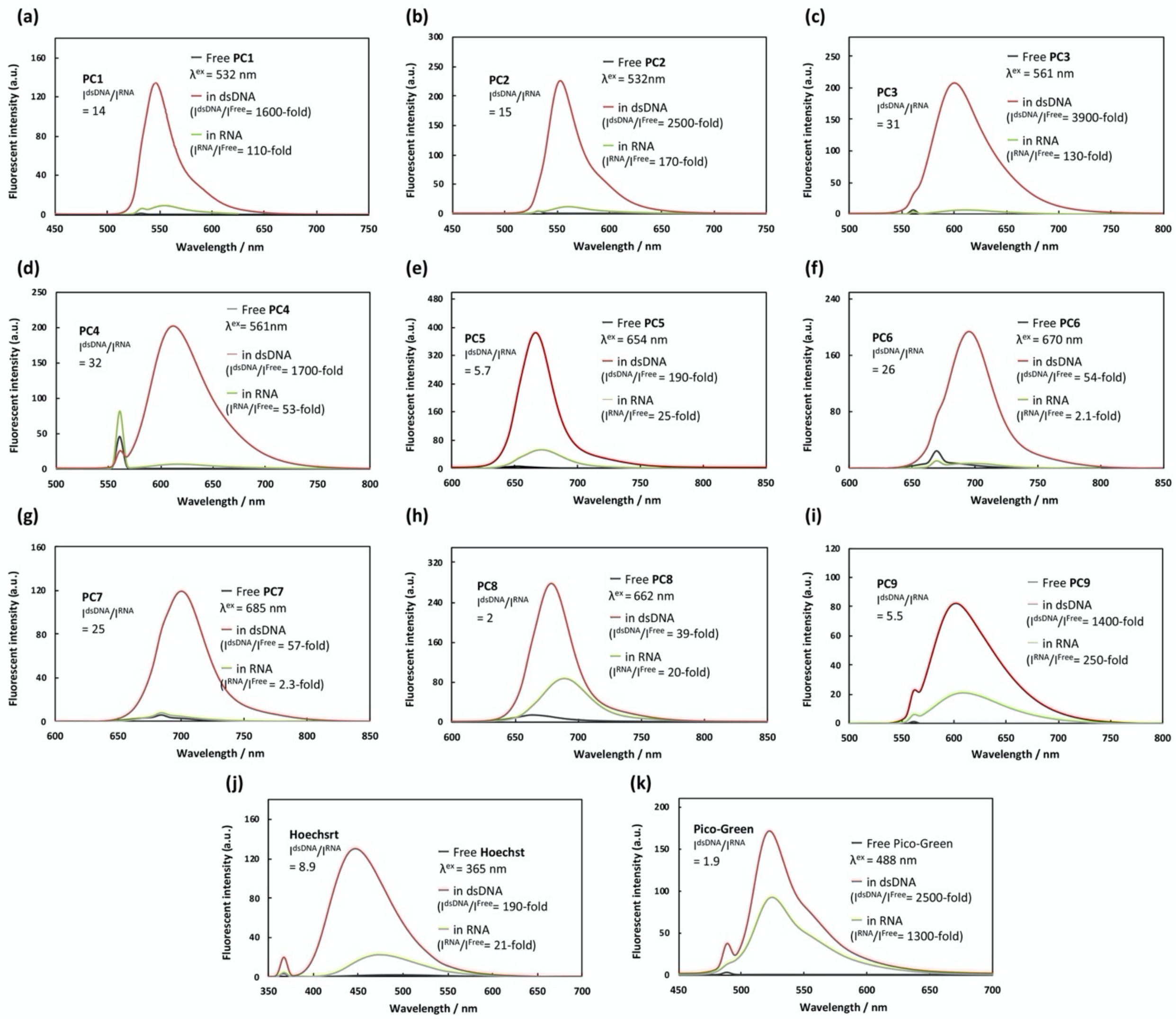

**Supplementary Fig. 2. Fluorescence spectra of N-aryl PC derivatives and two popular commercialized DNA probes in EDTA solution upon mixing excess of dsDNA and RNA.** Black lines indicate the fluorescence spectra of noted dyes in EDTA solution. Change of fluorescence spectra and increase of fluorescence intensity upon mixing excess of dsDNA (red-lines) and RNA (green-lines). Each graph shows the fluorescence intensity in arbitrary units (a. u.).

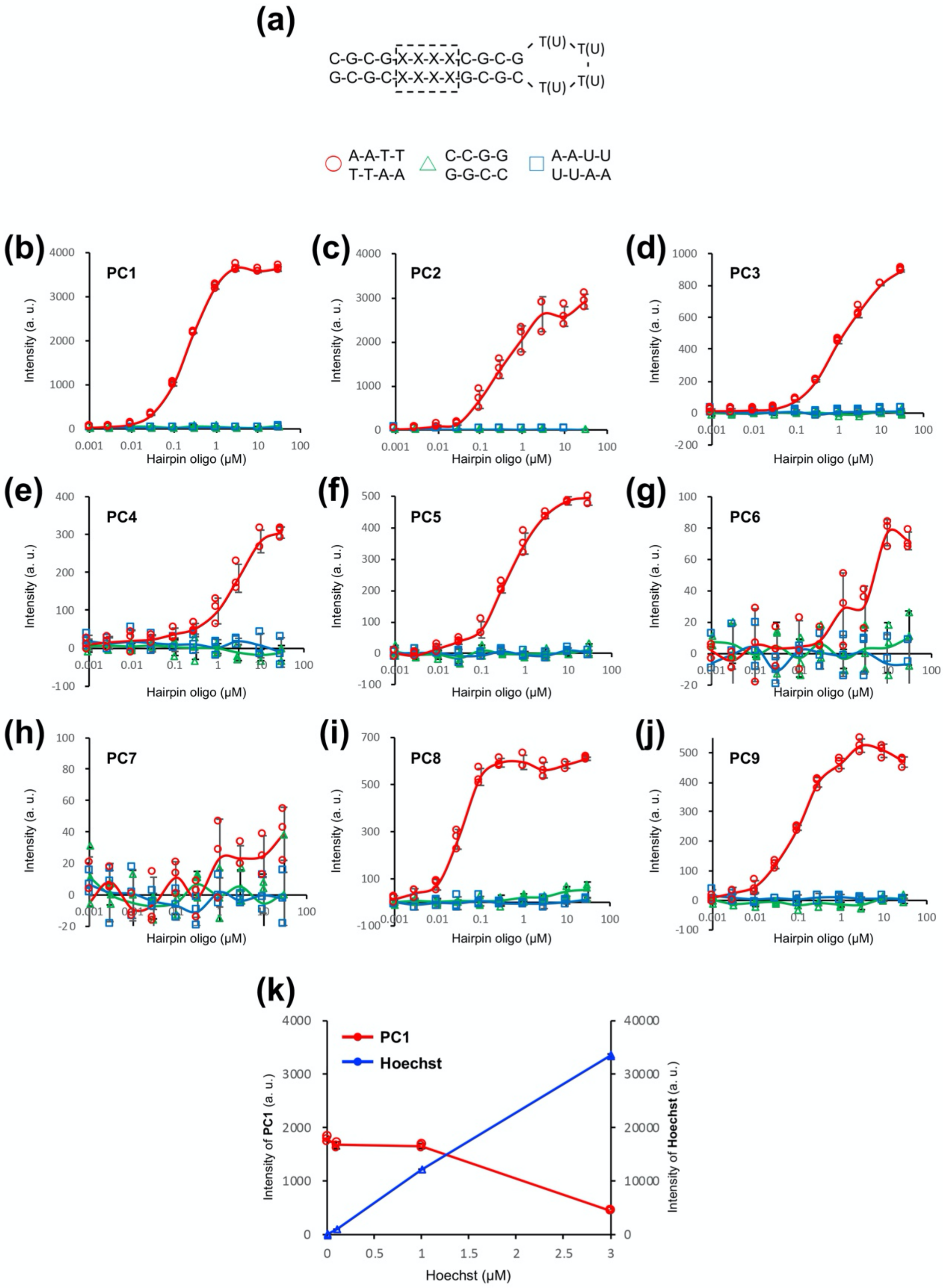


**Supplementary Fig. 3. Titration of N-aryl PC dyes with various concentration of hairpin DNA, RNA.** (a) The sequences of hairpin oligo DNA (red and green) and RNA (blue) **(b-j)** The titration curve of 100 nM PC dyes. Error bars represent mean ± s.d. from three independent replicates. **(k)** Competitive titration in DNA sequence between 100 nM **PC1** and different concentration of Hoechst 33342 using 1 μM $^{AATT}$DNA hairpin oligo. Each graph shows the fluorescence intensity in arbitrary units (a. u.).

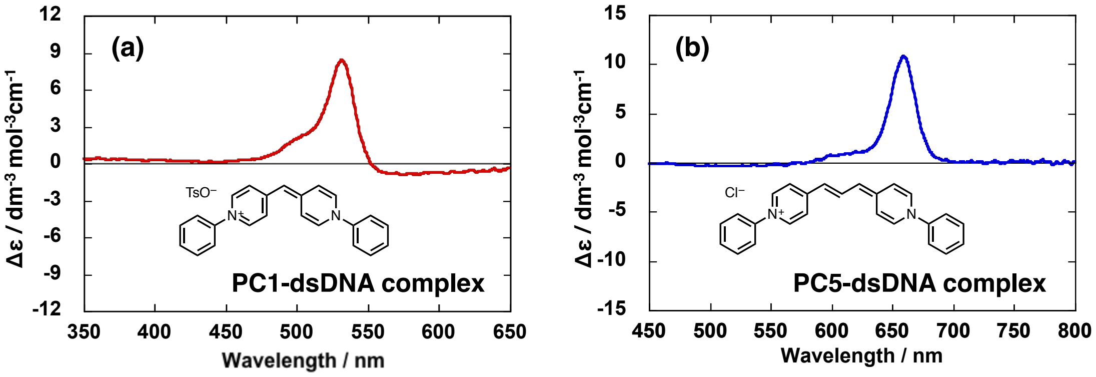


**Supplementary Fig. 4. Circular dichroism (CD) spectra.** (a) **PC1** ($1.4 \times 10^{-6}$ M) and (b) **PC5** ($2.1 \times 10^{-6}$ M) in the presence of excess dsDNA.

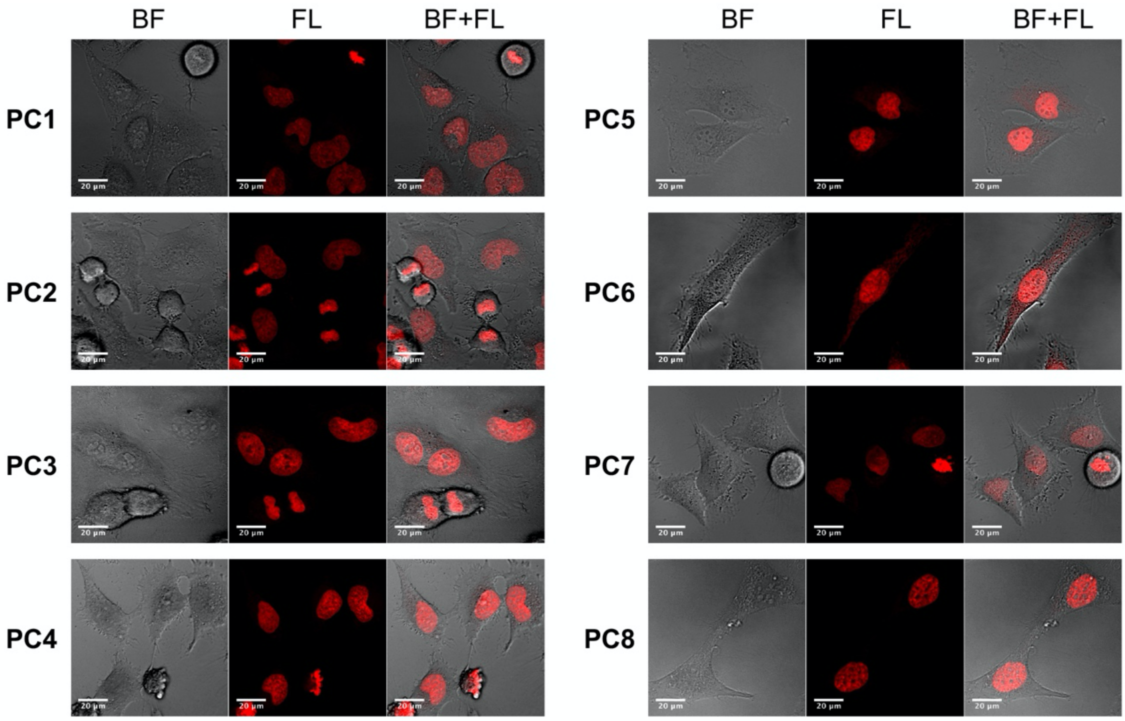


**Supplementary Fig. 5. N-aryl PC dyes stain nucleus in living HeLa cells.** HeLa cells were stained with each dye at the concentration of 1 µM. The fluorescence image stained with **PC1** were obtained emission spectrum between 517-693 nm excited with 514 nm. The images stained with **PC2-PC4** were obtained emission spectrum between 570-693 nm excited with 560 nm. The images stained with **PC5-PC8** were obtained the emission spectrum between 640-693 nm excited with 633 nm. Fluorescent images are maximum z-projections of total planes (1 µm intervals). BF; bright-field, FL; fluorescence, BF+FL; overlaid image of BF and FL. The representative images are shown from 2-5 similar images in each PC dye staining.

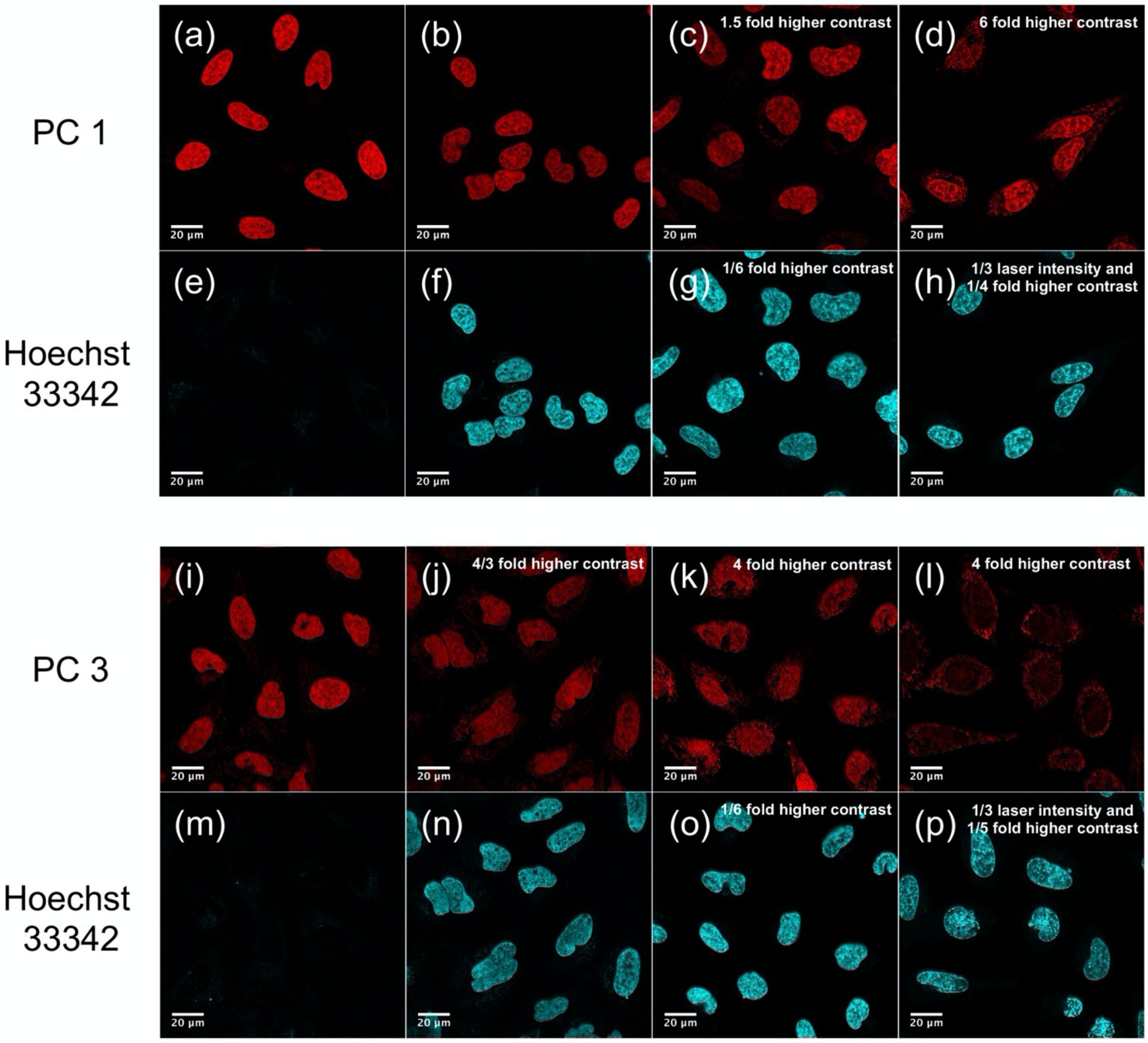


**Supplementary Fig. 6. Co-staining of N-aryl PC dyes with Hoechst 33342 in living HeLa cells. (a-h)** HeLa cells were co-stained with 100 nM **PC1** with 0 nM (a, e), 100 nM (b, f), 1 μM (c, g), and 3 μM (d, h) Hoechst 33342. **(i-p)** HeLa cells were co-stained with 100 nM **PC3** with Hoechst 33342; 0 nM (i, m), 100 nM (j, n), 1 μM (k, o), 3 μM (l, p). Note that N-aryl PC dyes are excluded from nucleus by Hoechst 33342 in dose dependent manner. The representative images are shown from 4 similar images in each staining condition.

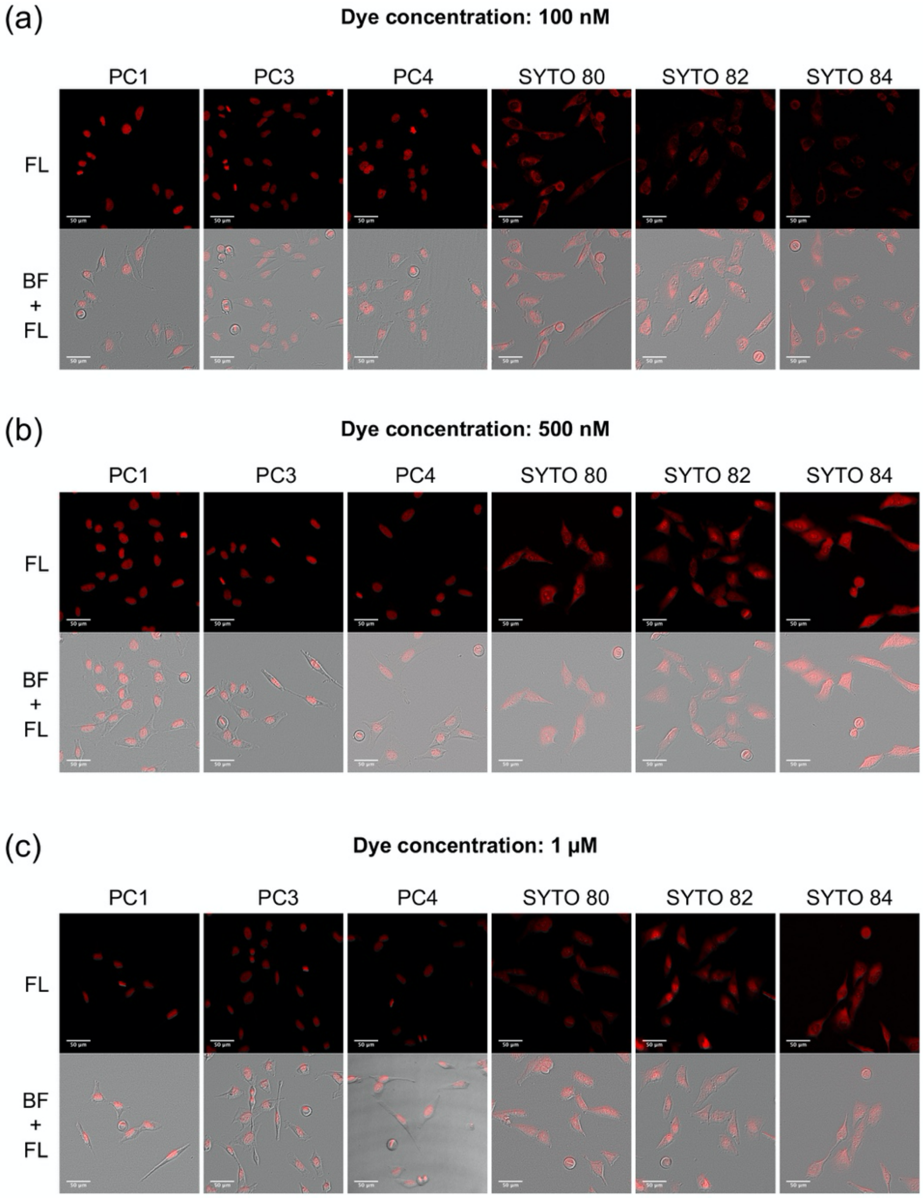


**Supplementary Fig. 7. Comparison of nuclear DNA stain between N-aryl PC dyes and SYTO orange dyes in living HeLa cells.** HeLa cells were stained with each dye at 100 nM (a), 500 nM (b) and 1 μM (c). The excitation laser lines were used into alignment with absorption peak wavelength of each dye (532 nm for **PC1**, 552 nm for **PC3**, 561 nm for **PC4**, 532 nm for **SYTO 80**, 543 nm for **SYTO 82**, 561 nm for **SYTO 84**) and those emission spectra were collected in 540-670 nm, 560-670 nm, 570-670 nm, 540-670 nm, 550-670 nm, 570-670 nm, respectively. Note that N-aryl PC dyes specifically stain cell nucleus and chromosome in all concentration tested, whereas SYTO series stain cytoplasm and nucleolus instead of specific labelling of nucleus. BF; bright-field, FL; fluorescence, BF+FL; overlaid image of BF and FL. Selected images in (a) and (b) were also used in Fig. 2a and Fig.2b, respectively. The representative images are shown from 3 similar images in each staining condition.

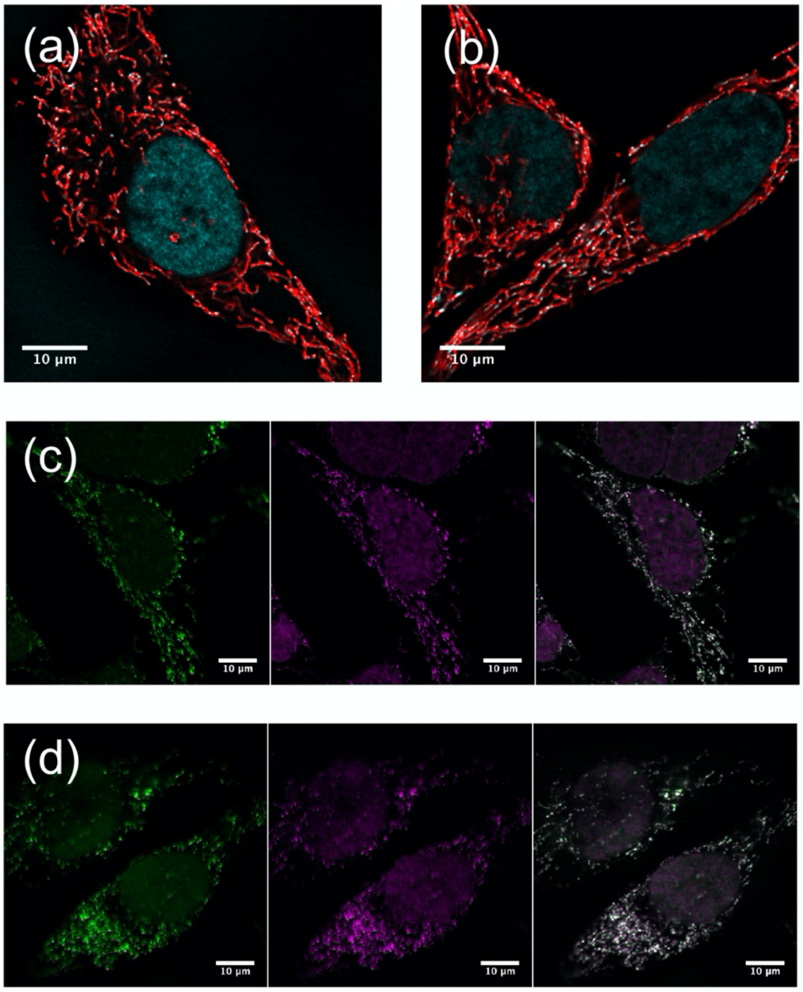


**Supplementary Fig. 8. Co-staining of N-aryl PC dyes with MitoTracker Deep Red (a-b) and SYBR-Green (c-d).** (a) Overlaid image of **PC1** (cyan) and MitoTracker Deep red (red). (b) Overlaid image of **PC3** (cyan) and MitoTracker Deep Red (red). Note that the cytoplasmic spots of PC dyes are localized in mitochondrial tubes. The representative images are shown from 8 and 7 similar images, respectively. (c-d) Linear-deconvolution images using reference spectra of selected pixels. Overlaid image was also shown in the right panel. (c) SYBR-Green and **PC1** are shown in green and magenta. (d) SYBR-Green and **PC3** are shown in green and magenta. The representative images are shown from 5 similar images in each staining condition.

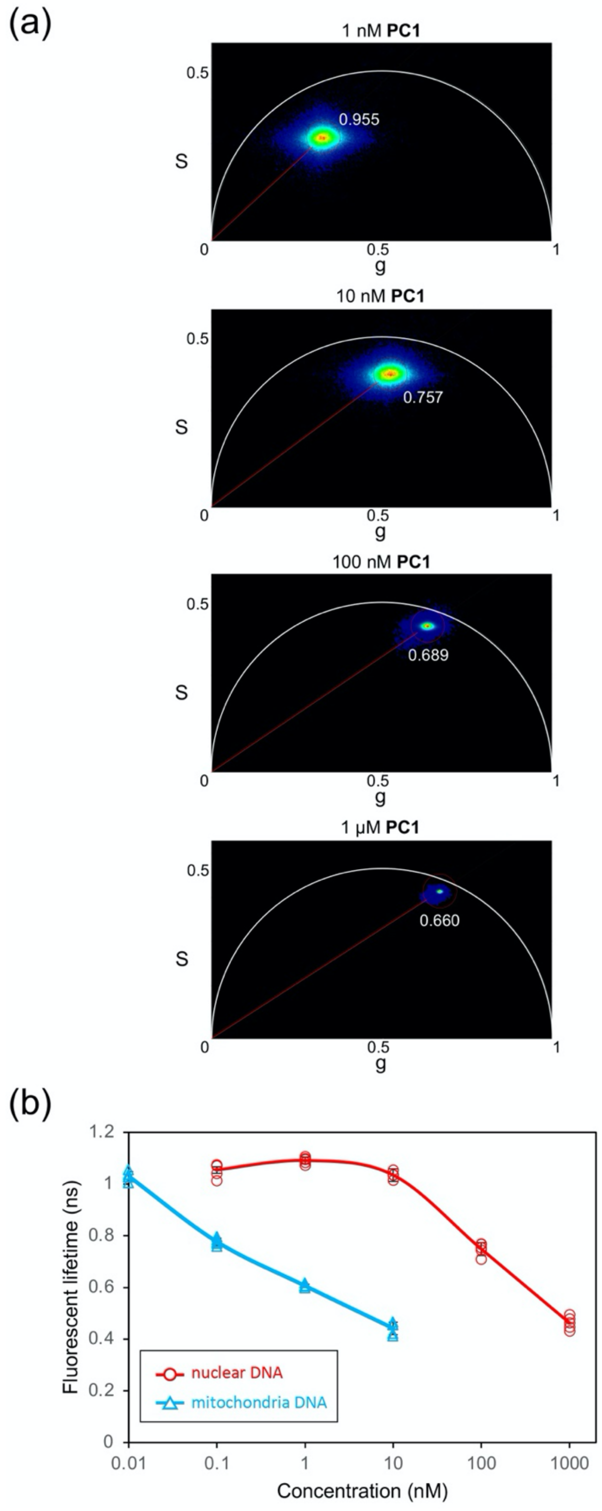


**Supplementary Fig. 9. Changes in the fluorescent lifetime of PC1 in concentration dependent manner.** (a) Changes in the fluorescent lifetime of **PC1** with different concentrations of **PC1** in 100 nM pBruescript II SK+ vector. (b) Changes in the fluorescent lifetime of **PC1** in nuclear DNA and mitochondria DNA of HeLa cells stained with different concentrations of **PC1**. Error bars represent mean ± s.d. from 5 independent replicates.

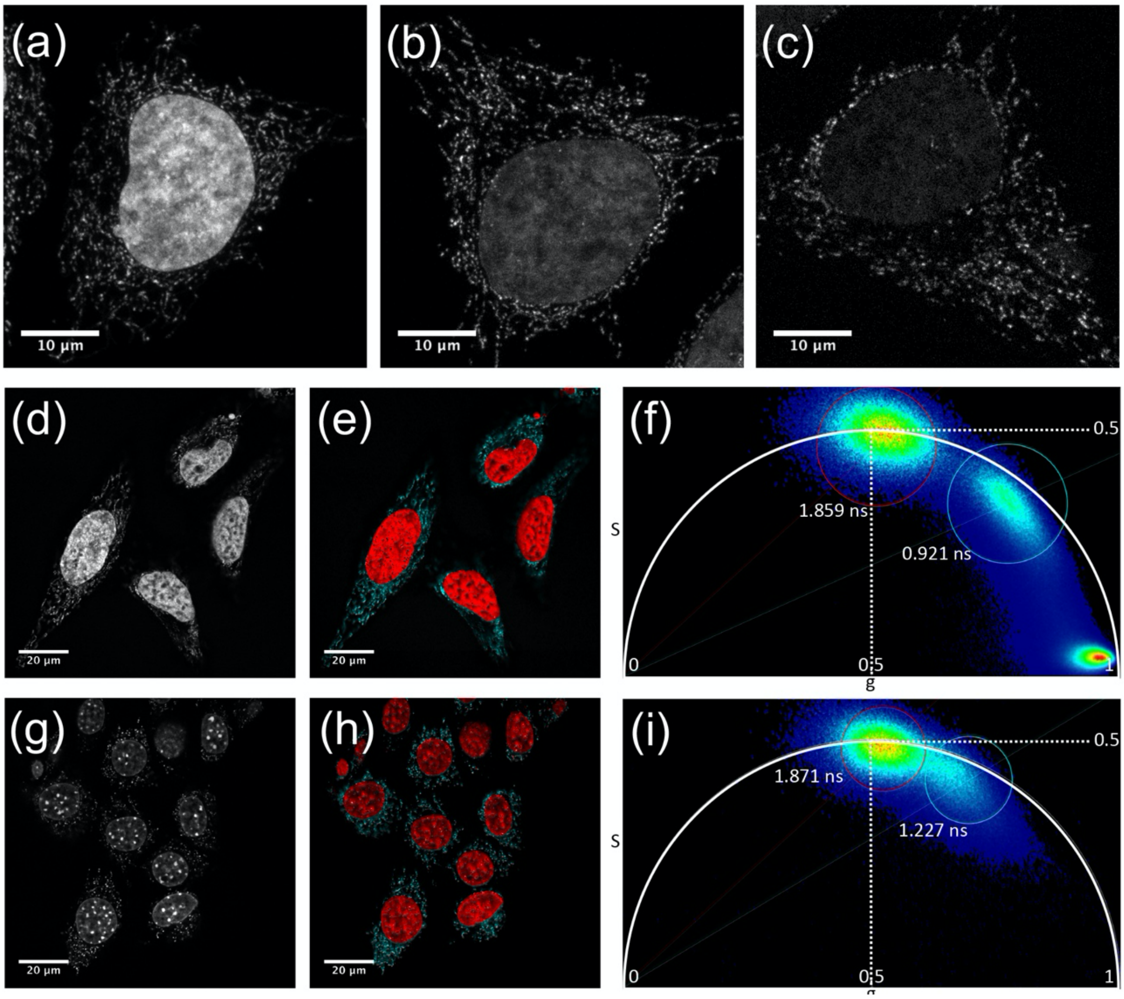


**Supplementary Fig. 10. Discrimination between nuclear DNA and mt-DNA with fluorescence lifetime of PC3.** Concentration dependence of staining pattern with **PC3**. (a) 10 nM, (b) 1 nM, (c) 100 pM. The images are maximum z-projections of total planes (0.3 µm intervals). The representative images are shown from 3-4 similar images in each concentration. **(d-i)** Fluorescent intensity images (d, g) and FLIM based separation images of nuclear DNA and mitochondrial DNA (e, h) by phasor plot analysis (f, i). The pseudo colors of (e, h) is correspond to the colors of circles in (f, i). HeLa cells (d-f) and NIH3T3 (g-i) were stained with 10 nM and 30 nM **PC3**, respectively and the fluorescent spectrum were collected between 570-650 nm excited at 561 nm. The representative images are shown from 6 and 8 similar images, respectively.

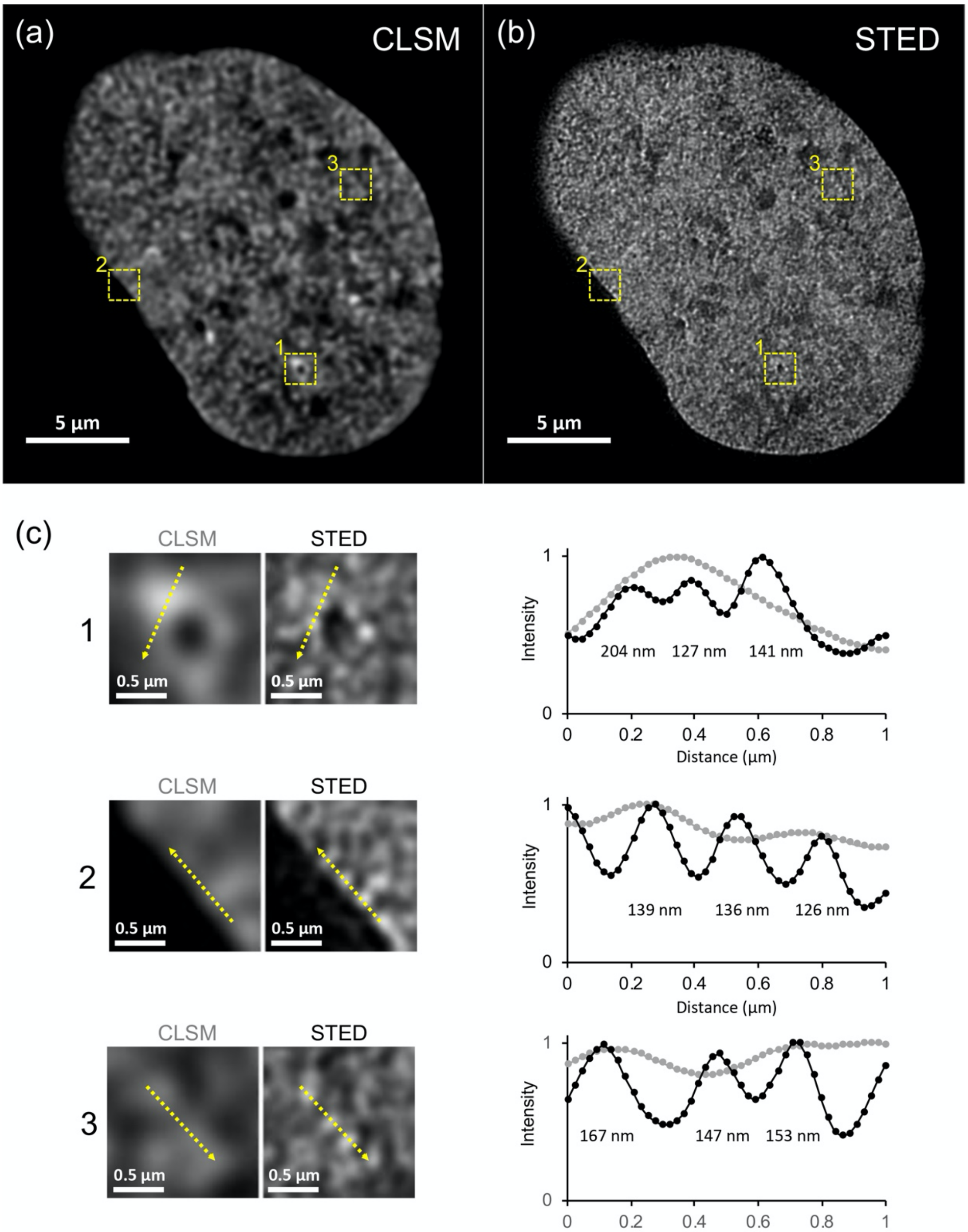


**Supplementary Fig. 11. Comparison of confocal and SPLIT-STED imaging in living HeLa cells stained with PC3. (a)** Confocal and **(b)** super-resolution images of nuclear DNA stained with 1 µM PC3. **(c)** Enlarged images of the yellow dotted square region of **(a, b)** and examples of normalized fluorescence intensity profiles obtained from the region indicated by arrows. Line profiles in STED and confocal image are shown in black and gray, respectively. FWHM values estimated by fitting with a Gaussian function are also indicated in the black line profile. The representative images are shown from 6 similar images.

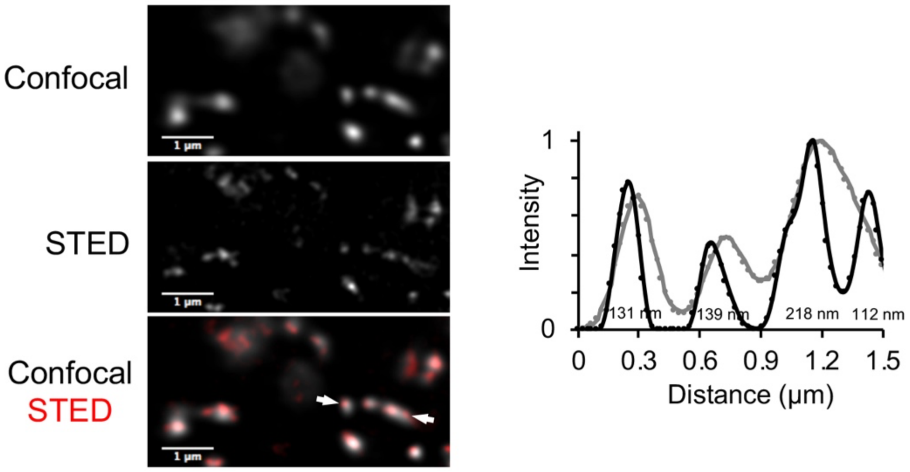


**Supplementary Fig. 12. Comparison of confocal and SPLIT-STED imaging in living NIH3T3 cells stained with PC3.** NIH/3T3 cells were stained with **PC3** at 30 nM concentration. An example of normalized fluorescence intensity profile obtained from the region between arrows. Line profiles in STED and confocal image are shown in black and gray, respectively. FWHM values estimated by fitting with a Gaussian function are also indicated in the black line profile. The representative image is shown from 5 similar images.

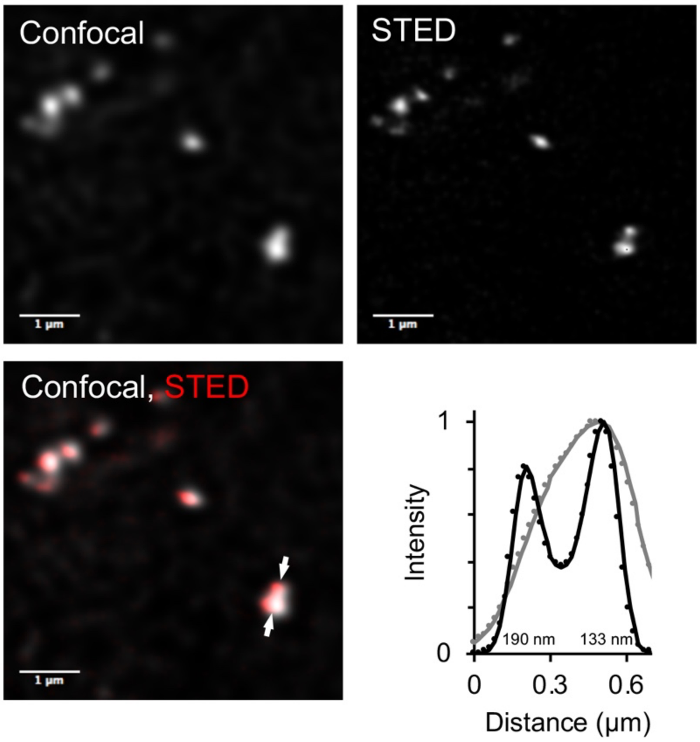


**Supplementary Fig. 13. Comparison of confocal and SPLIT-STED imaging in living Arabidopsis root cells stained with PC3.** Arabidopsis root cells were stained with **PC3** at 300 nM concentration. An example of normalized fluorescence intensity profile obtained from the region between arrows. F Line profiles in STED and confocal image are shown in black and gray, respectively. FWHM values estimated by fitting with a Gaussian function are also indicated in the black line profile. The representative images are shown from 16 similar images.

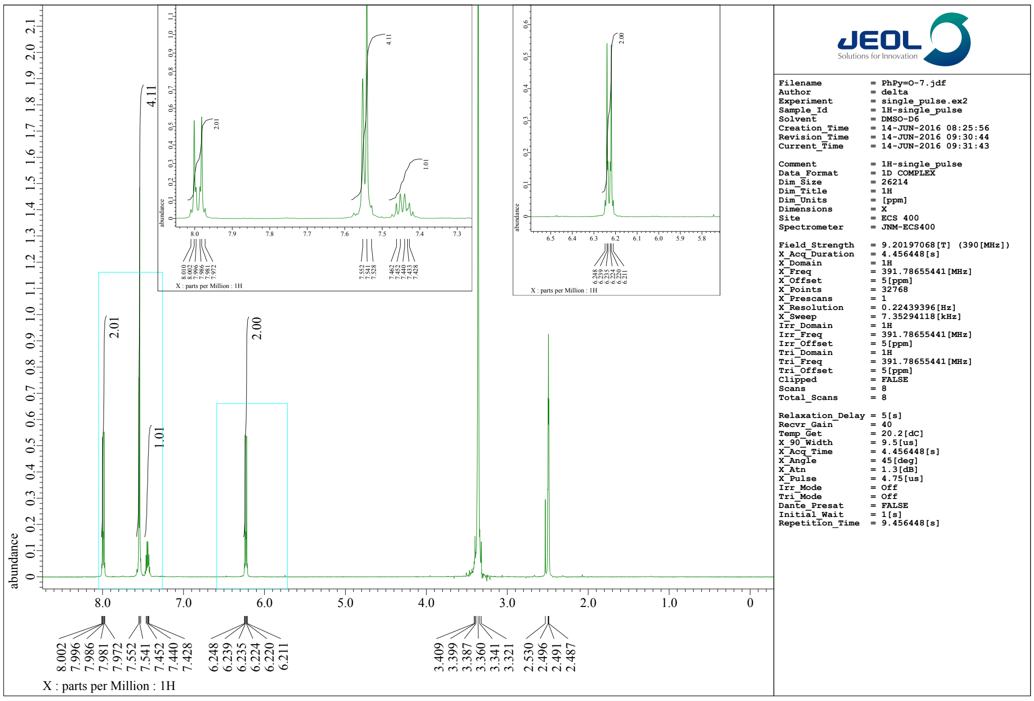

**NMR chart 1**. $^{1}$H-NMR (400 MHz) spectrum of **1a** in dimethyl sulfoxide-$d_6$ ($d$-DMSO)

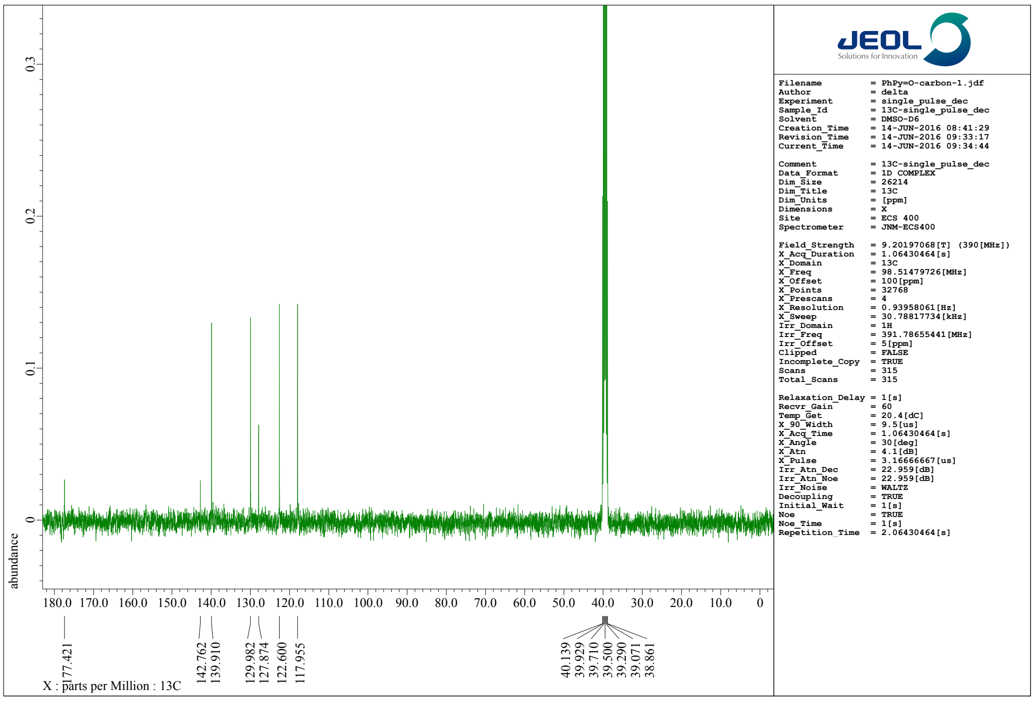

**NMR chart 2**. $^{13}$C-NMR (100 MHz) spectrum of **1a** in dimethyl sulfoxide-$d_6$ ($d$-DMSO).

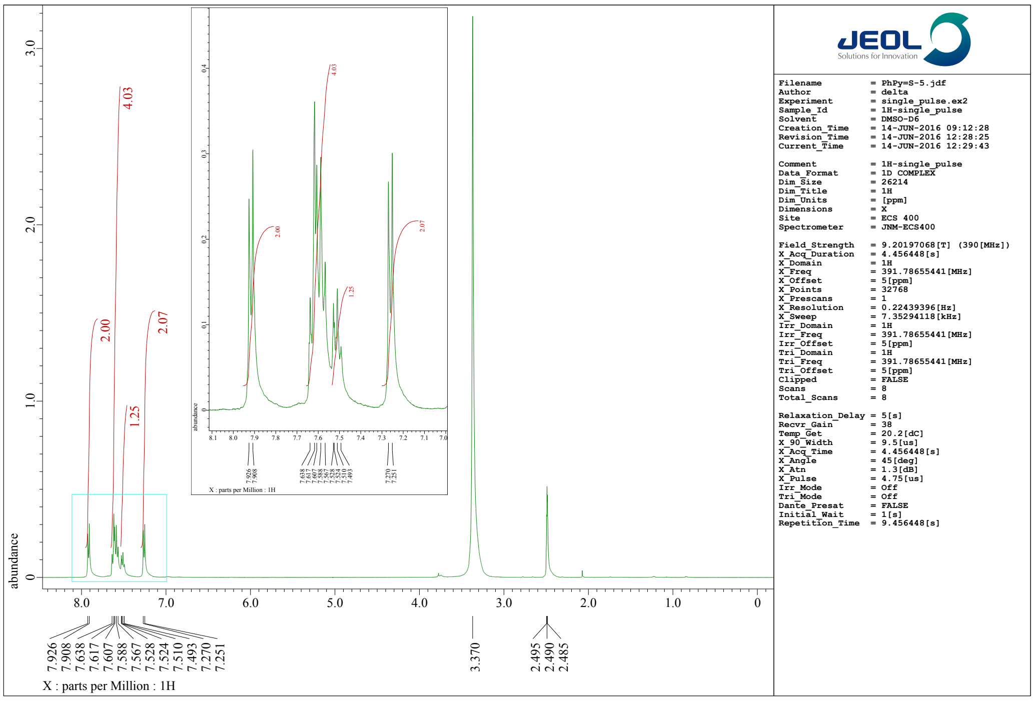


**NMR chart 3**. $^{1}$H-NMR (400 MHz) spectrum of **1b** in dimethyl sulfoxide-*d₆* (*d*-DMSO).

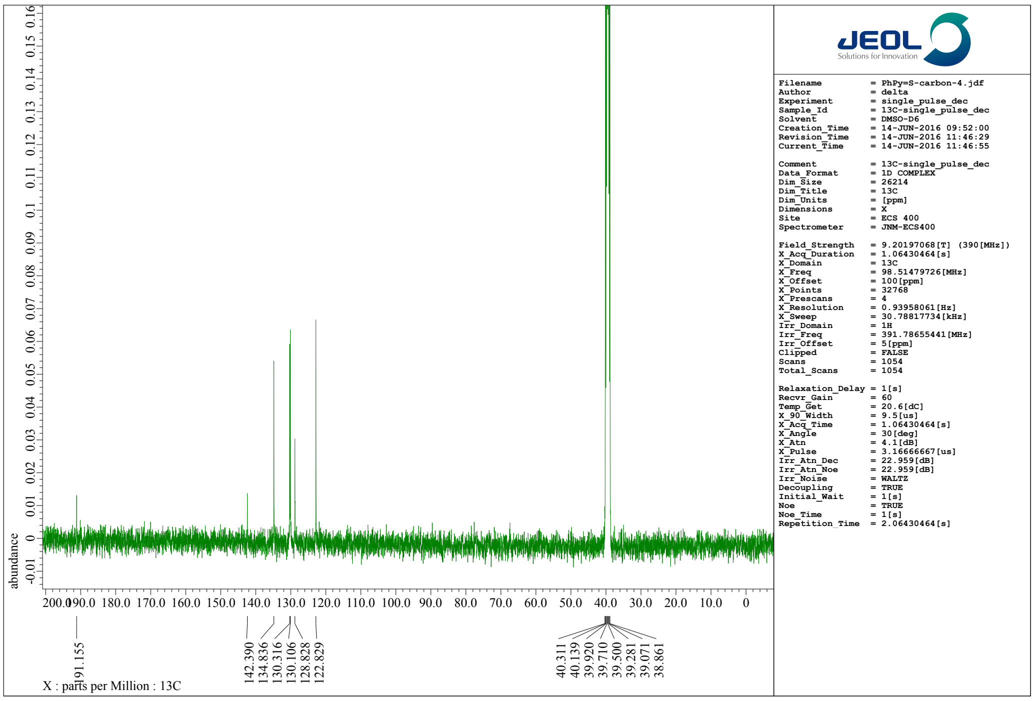


**NMR chart 4**. $^{13}$C-NMR (100 MHz) spectrum of **1b** in dimethyl sulfoxide-*d₆* (*d*-DMSO).

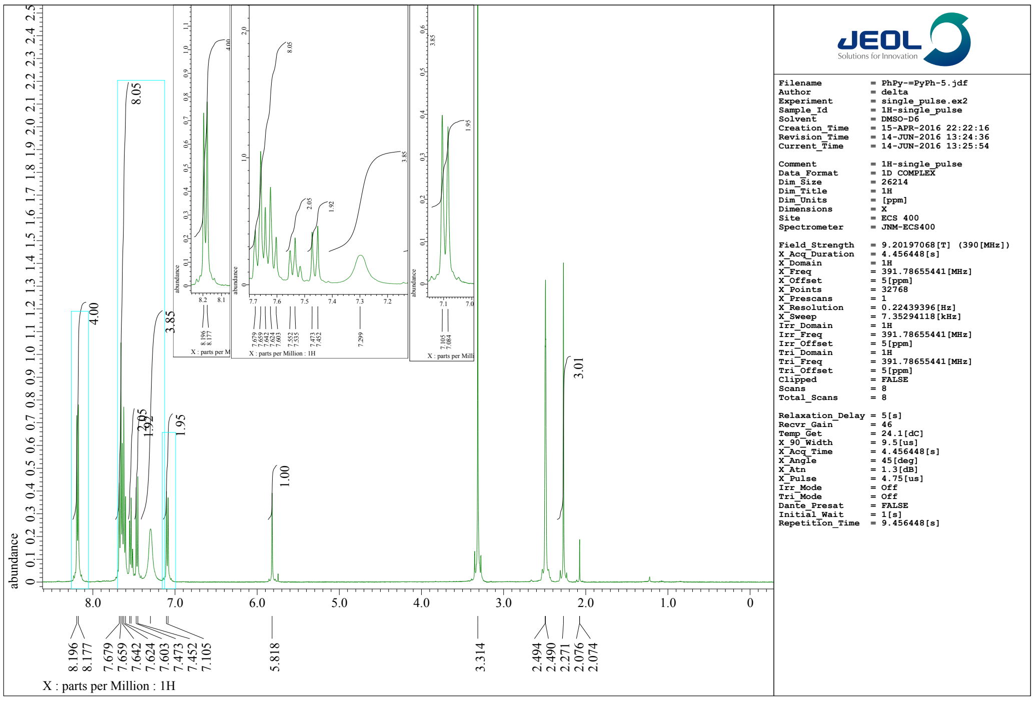


**NMR chart 5**. $^{1}$H-NMR (400 MHz) spectrum of **PC1** in dimethyl sulfoxide-$d_6$ (*d*-DMSO).

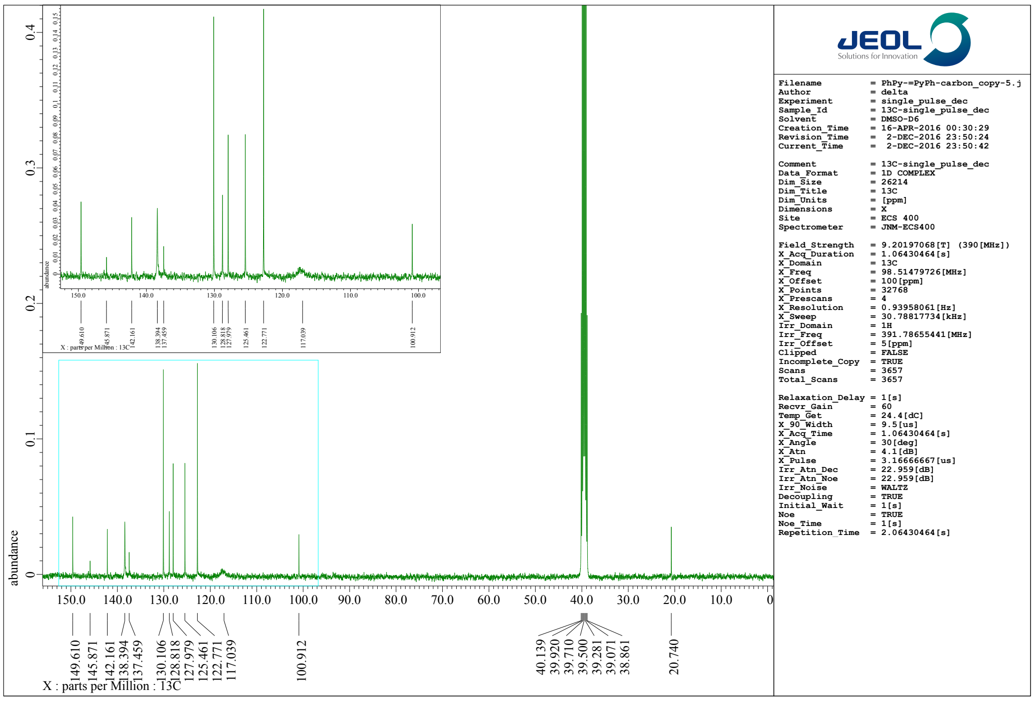


**NMR chart 6**. $^{13}$C-NMR (100 MHz) spectrum of **PC1** in dimethyl sulfoxide-d6 (d-DMSO)

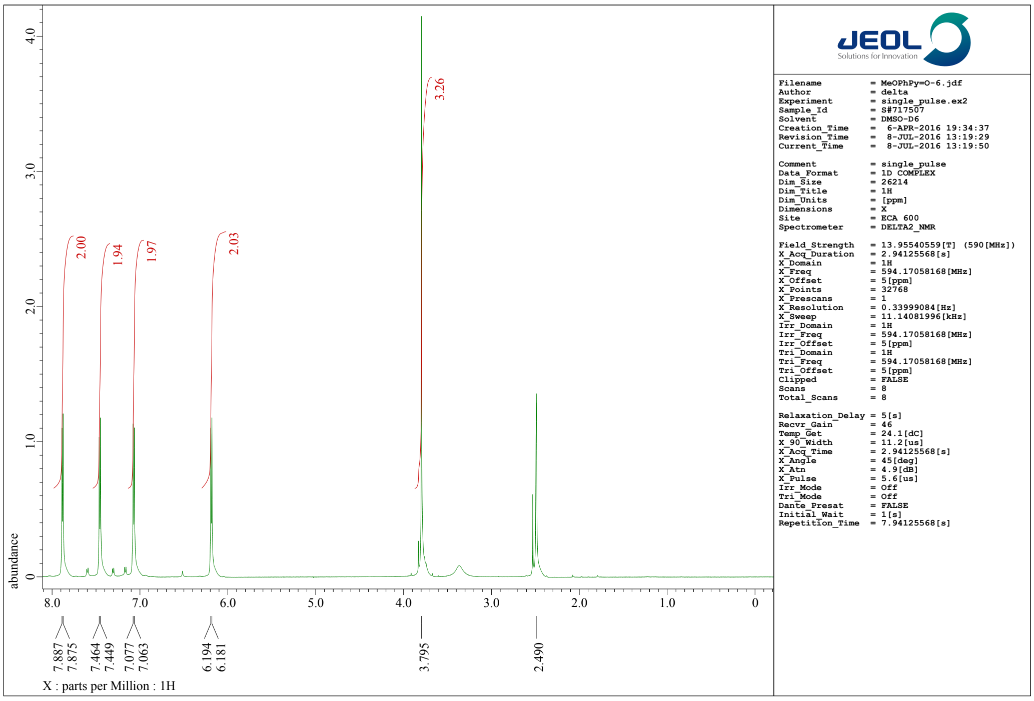


**NMR chart 7**. $^{1}$H-NMR (600 MHz) spectrum of **2a** in dimethyl sulfoxide-*d₆* (*d*-DMSO).

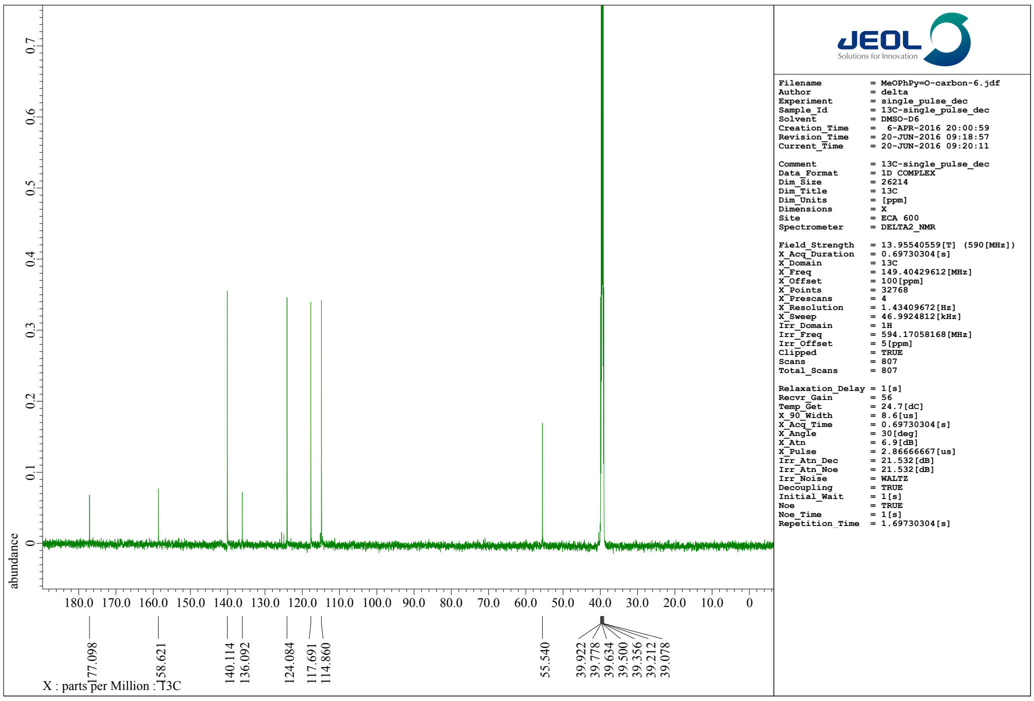


**NMR chart 8**. $^{13}$C-NMR (150 MHz) spectrum of **2a** in dimethyl sulfoxide-d6 (d-DMSO)

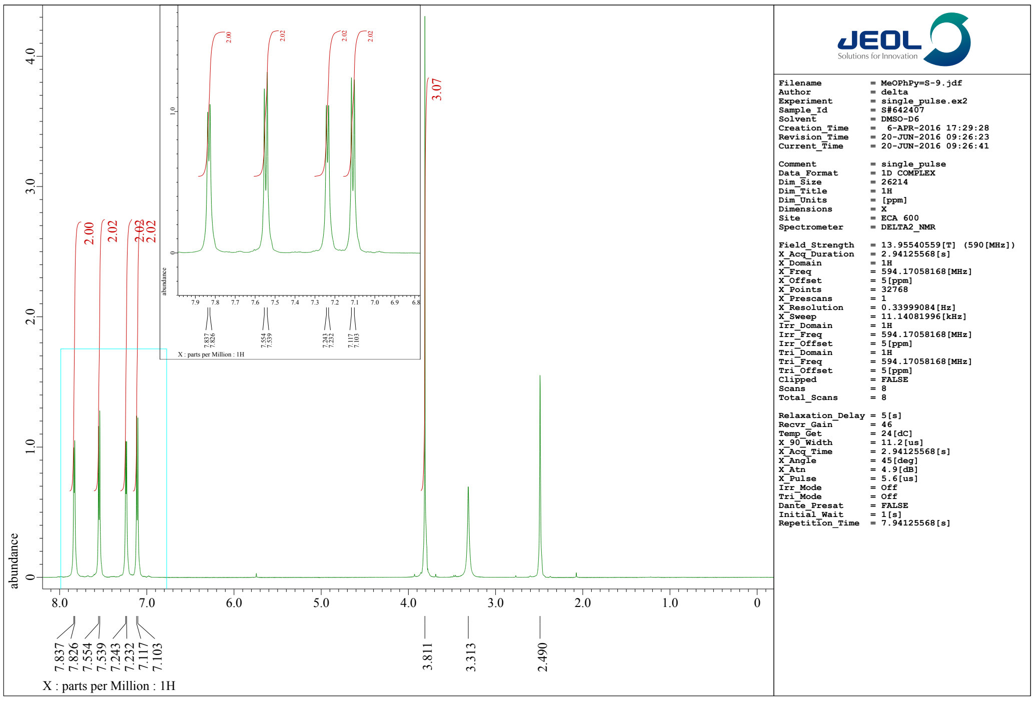


**NMR chart 9**. $^{1}$H-NMR (600 MHz) spectrum of **2b** in dimethyl sulfoxide-$d_6$ ($d$-DMSO).

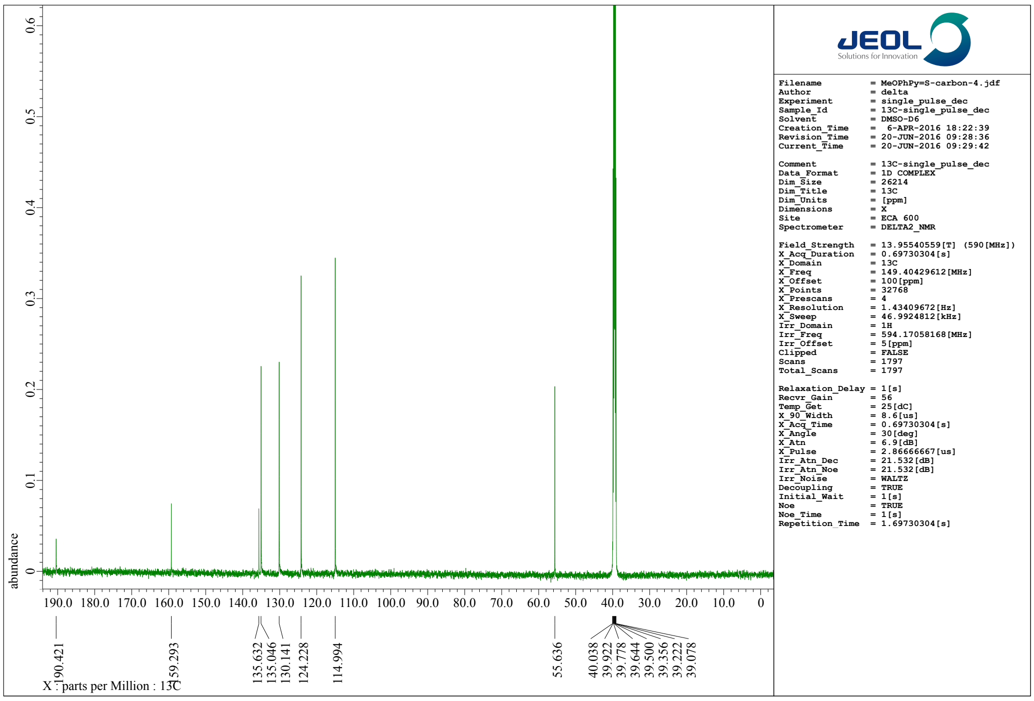


**NMR chart 10.** $^{13}$C-NMR (150 MHz) spectrum of **2b** in dimethyl sulfoxide-$d_6$ ($d$-DMSO).

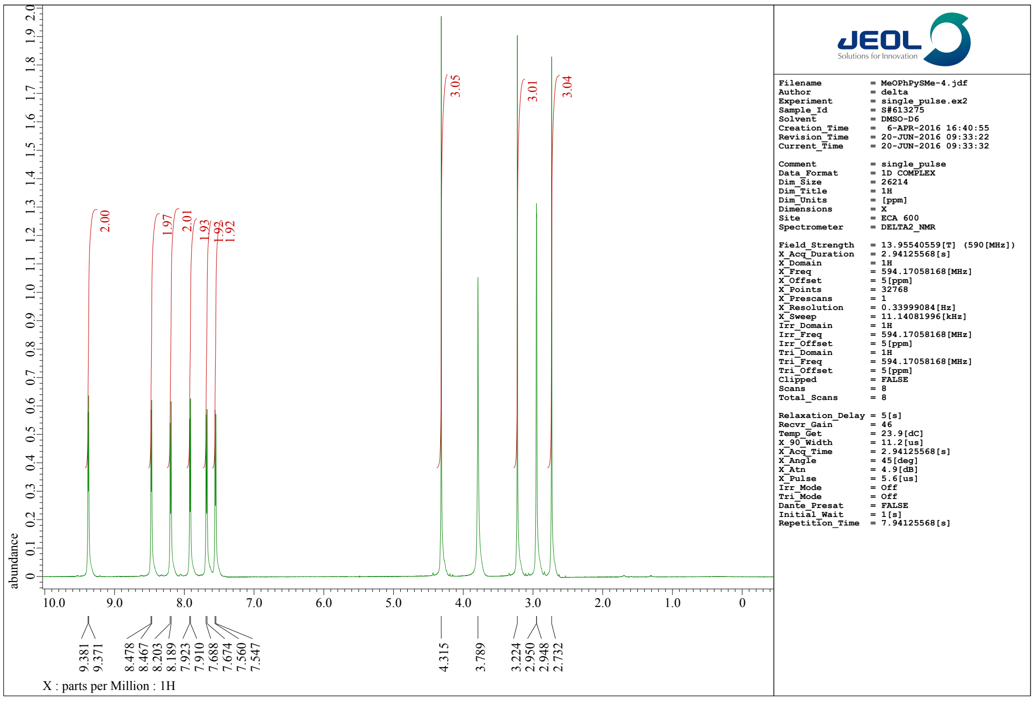


**NMR chart 11**. $^{1}$H-NMR (600 MHz) spectrum of **2c** in dimethyl sulfoxide-$d_6$ (*d*-DMSO).

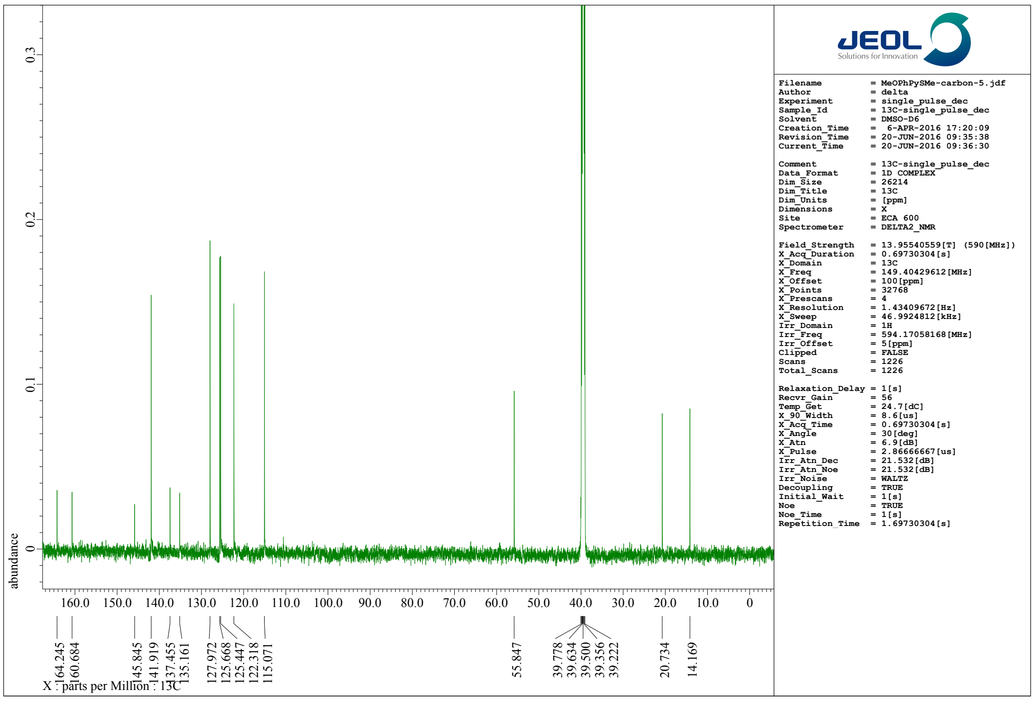


**NMR chart 12**. $^{13}$C-NMR (150 MHz) spectrum of **2c** in dimethyl sulfoxide-$d_6$ (*d*-DMSO).

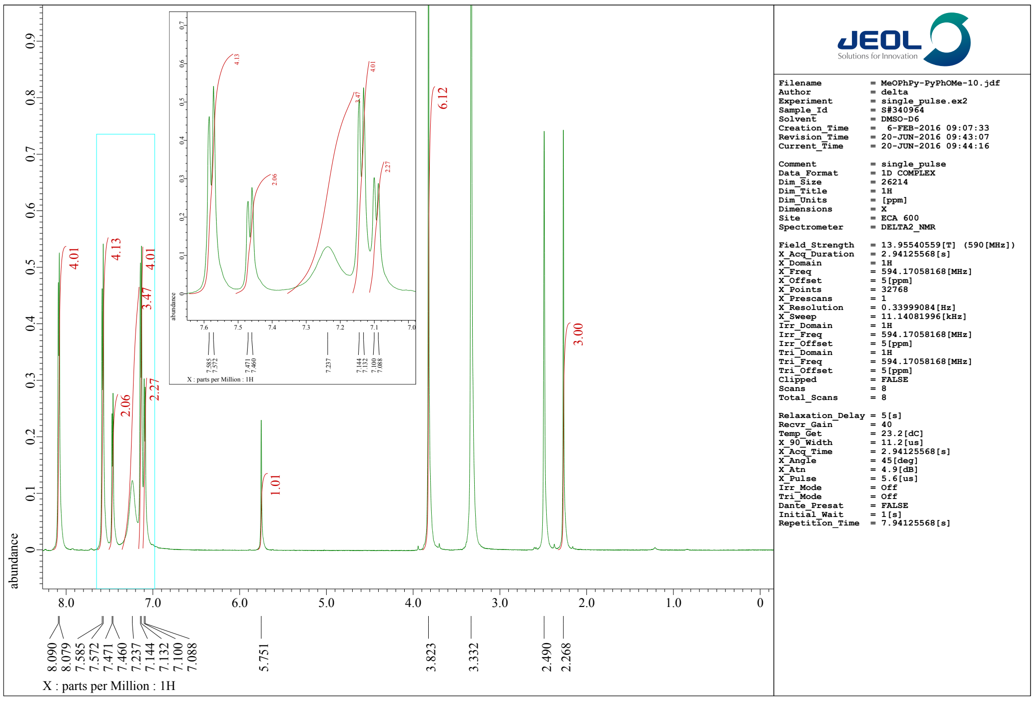


**NMR chart 13.** $^{1}H$-NMR (600 MHz) spectrum of **PC2** in dimethyl sulfoxide-$d_6$ ($d$-DMSO).

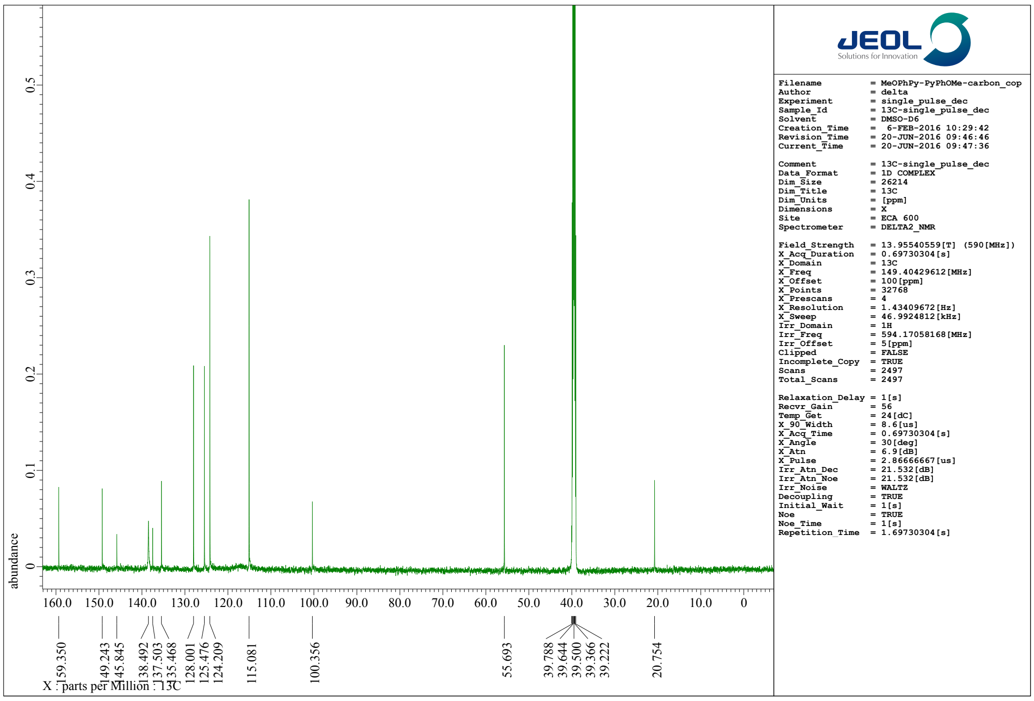


**NMR chart 14**. $^{13}C$-NMR (150 MHz) spectrum of **PC2** in dimethyl sulfoxide-$d_6$ ($d$-DMSO).

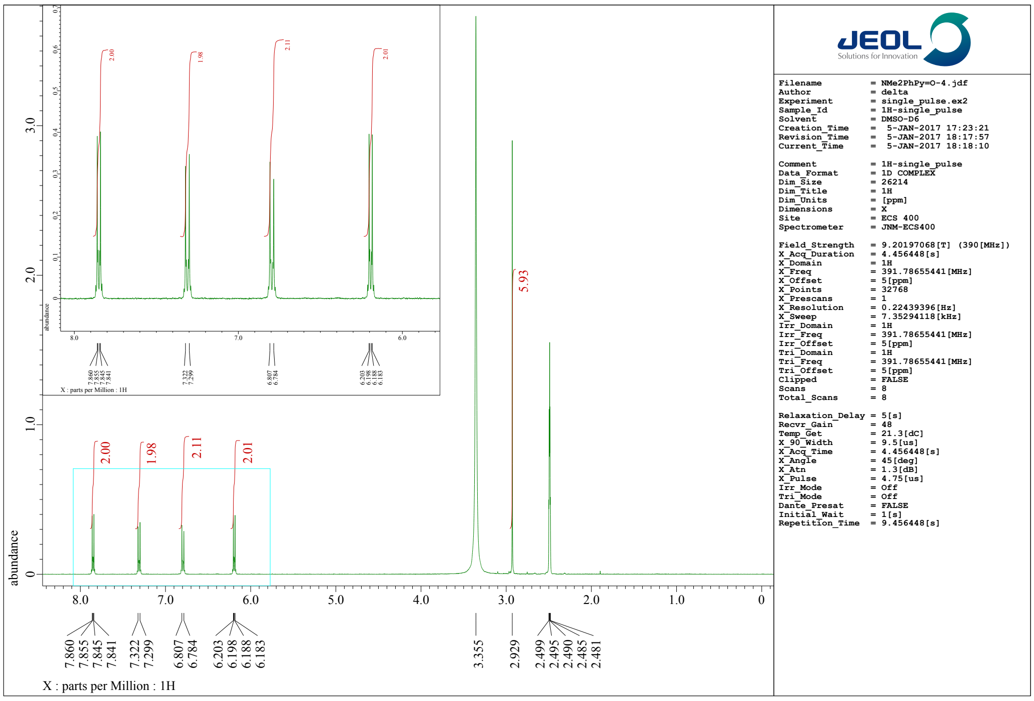


**NMR chart 15**. $^{1}$H-NMR (400 MHz) spectrum of **3a** in dimethyl sulfoxide-$d_6$ (*d*-DMSO).

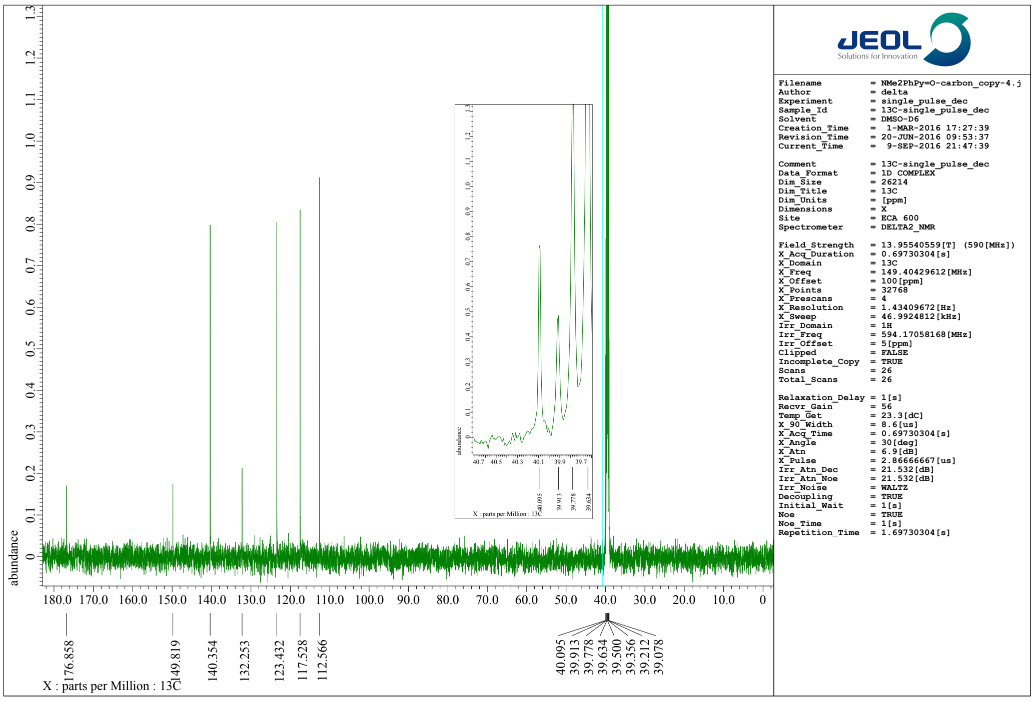


**NMR chart 16**. $^{13}$C-NMR (150 MHz) spectrum of **3a** in dimethyl sulfoxide-$d_6$ (*d*-DMSO).

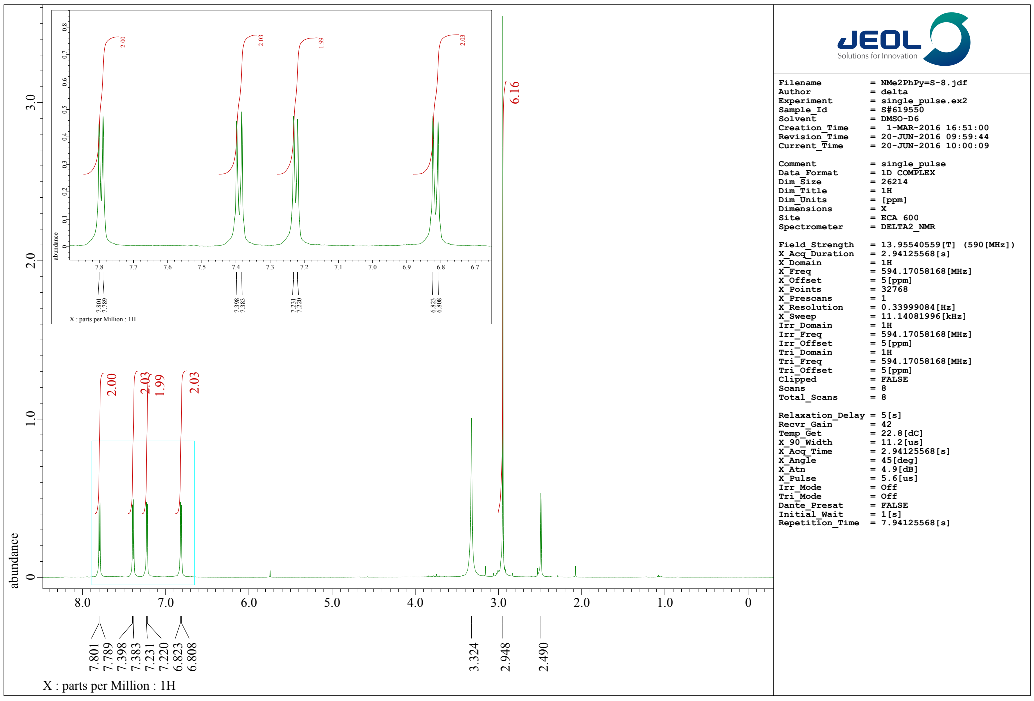


**NMR chart 17**. $^{1}$H-NMR (600 MHz) spectrum of **3b** in dimethyl sulfoxide-$d_6$ ($d$-DMSO).

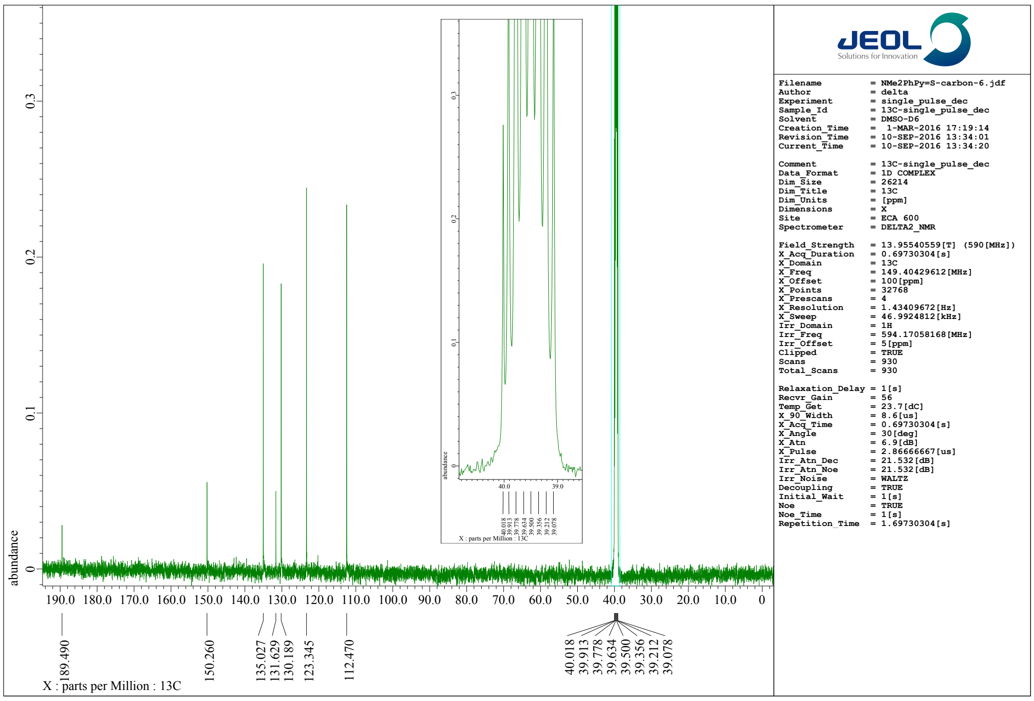


**NMR chart 18**. $^{13}$C-NMR (150 MHz) spectrum of **3b** in dimethyl sulfoxide-$d_6$ ($d$-DMSO).

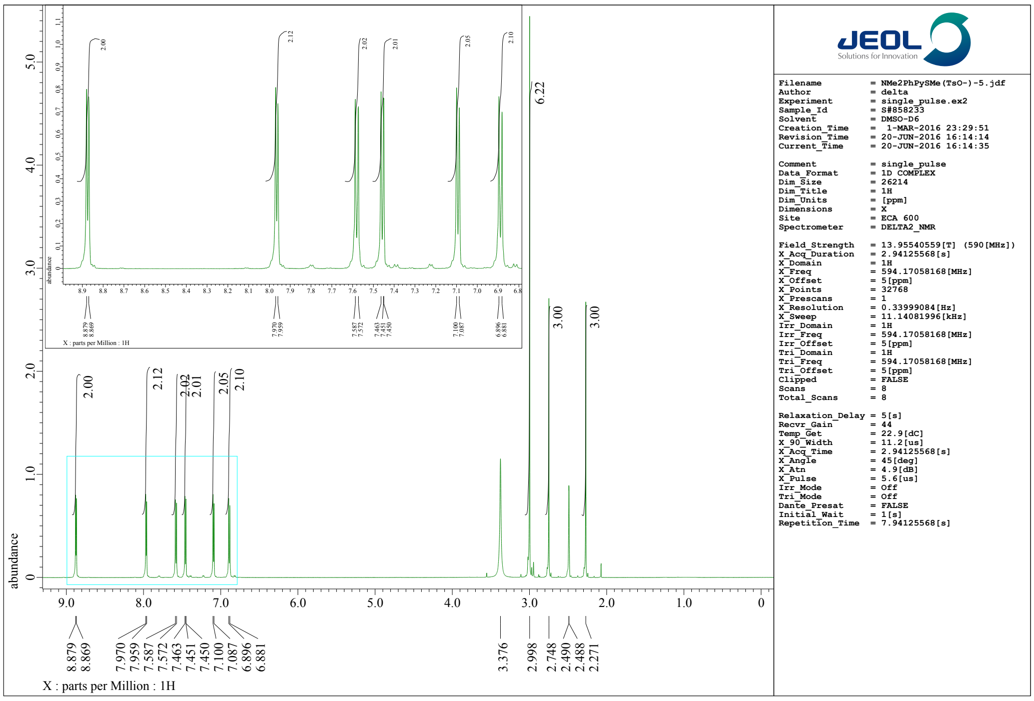


**NMR chart 19**. $^{1}$H-NMR (600 MHz) spectrum of **3c** in dimethyl sulfoxide-$d_6$ ($d$-DMSO).

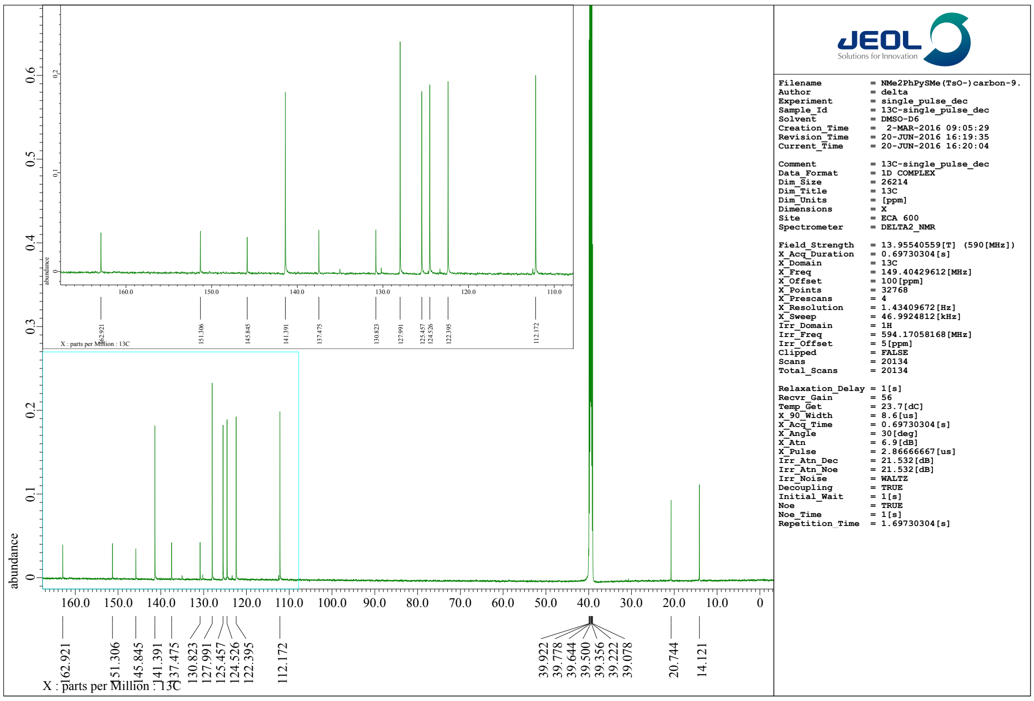


**NMR chart 20**. $^{13}$C-NMR (150 MHz) spectrum of **3c** in dimethyl sulfoxide-$d_6$ ($d$-DMSO).

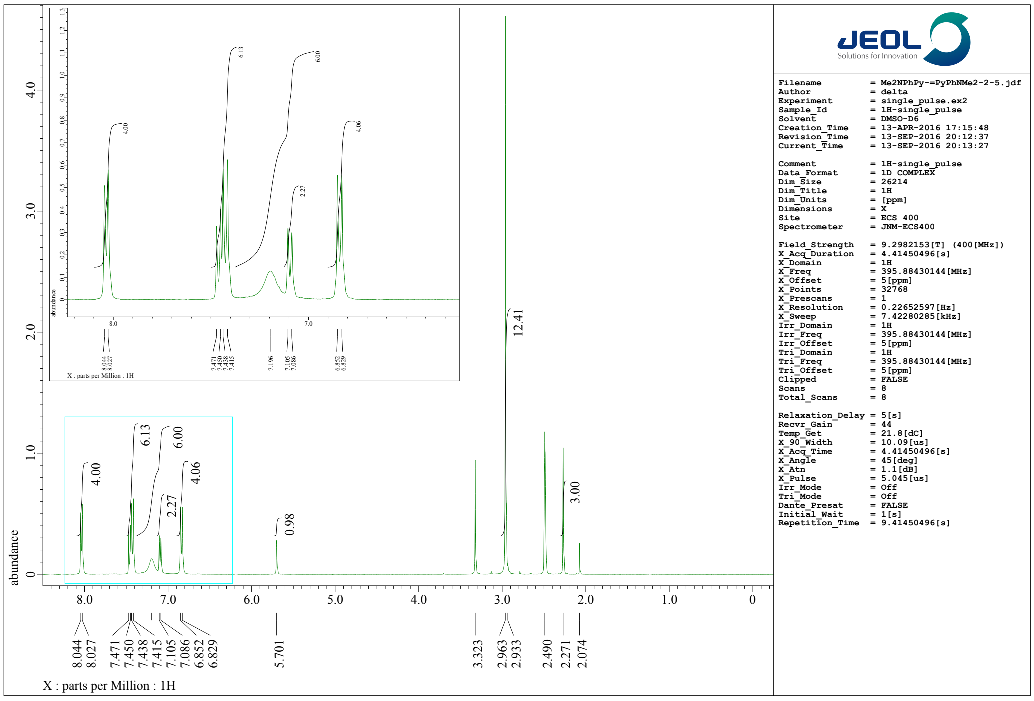


**NMR chart 21**. $^{1}$H-NMR (400 MHz) spectrum of **PC3** in dimethyl sulfoxide-$d_6$ (*d*-DMSO).

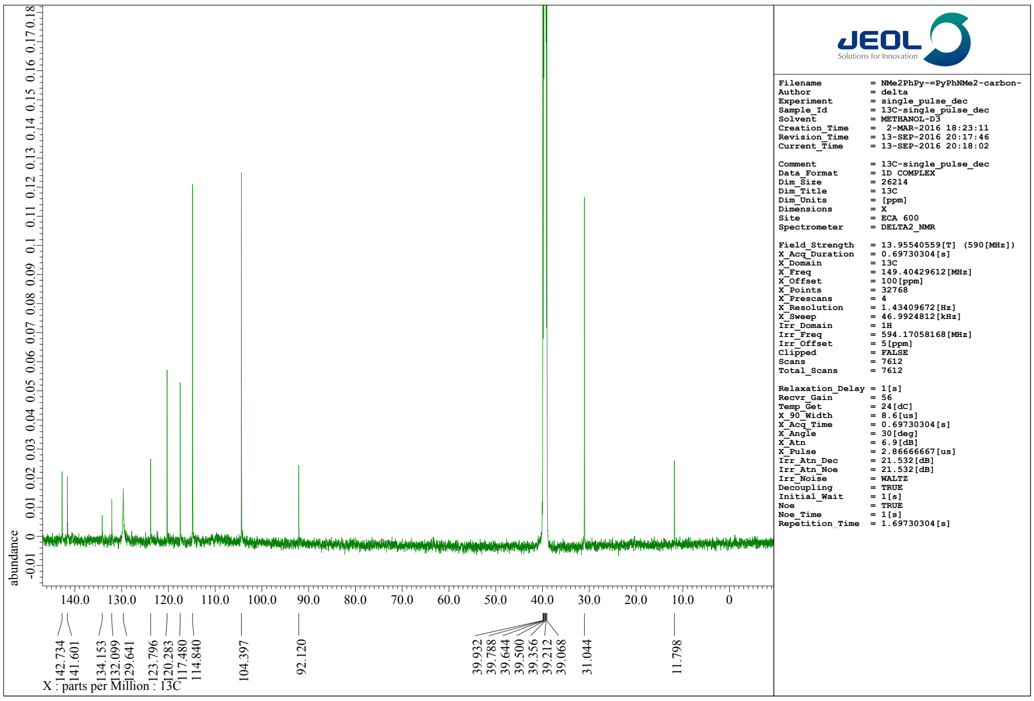


**NMR chart 22**. $^{13}$C-NMR (150 MHz) spectrum of **PC3** in dimethyl sulfoxide-$d_6$ (*d*-DMSO).

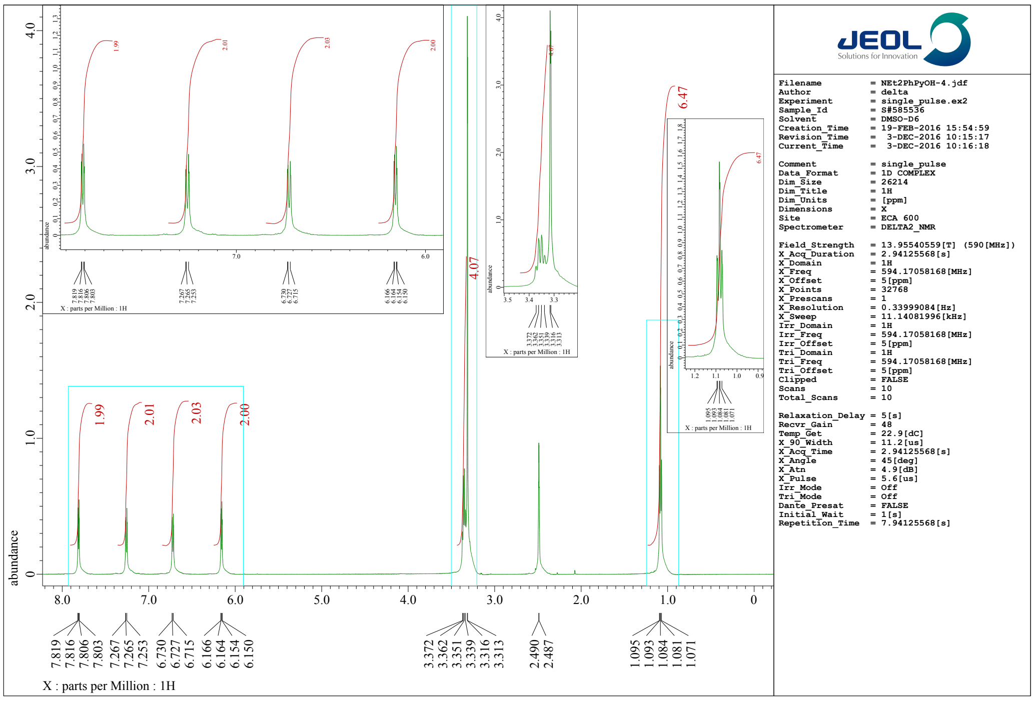


**NMR chart 23**. $^{1}$H-NMR (600 MHz) spectrum of **4a** in dimethyl sulfoxide-$d_6$ (*d*-DMSO).

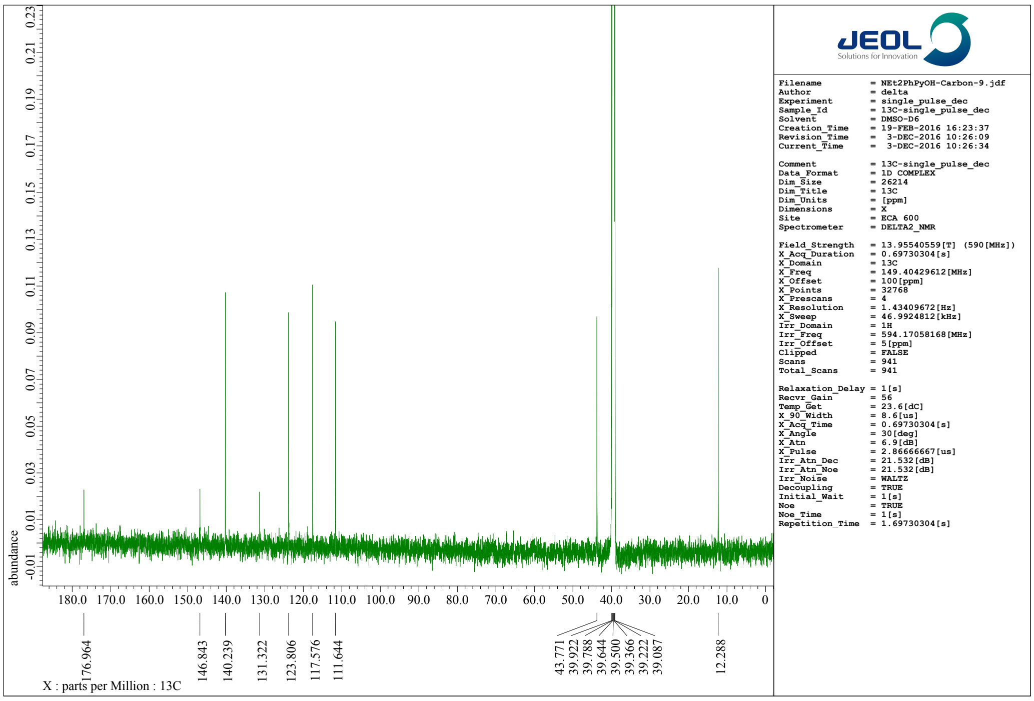


**NMR chart 24**. $^{13}$C-NMR (150 MHz) spectrum of **4a** in dimethyl sulfoxide-$d_6$ (*d*-DMSO).

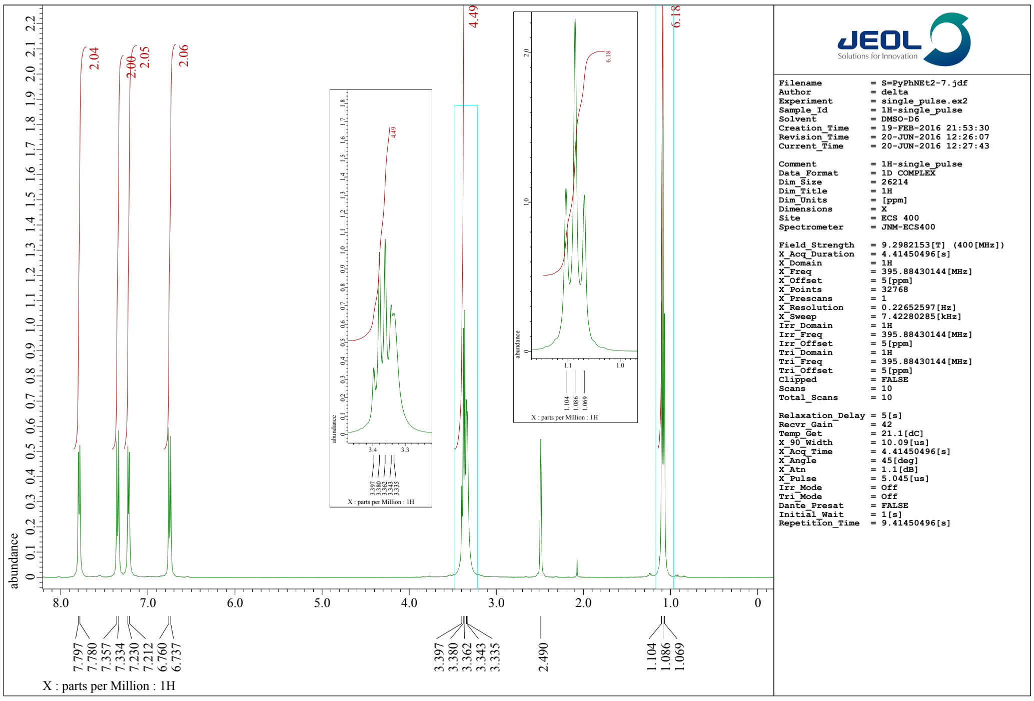


**NMR chart 25**. $^{1}$H-NMR (400 MHz) spectrum of **4b** in dimethyl sulfoxide-*d*$_6$ (*d*-DMSO).

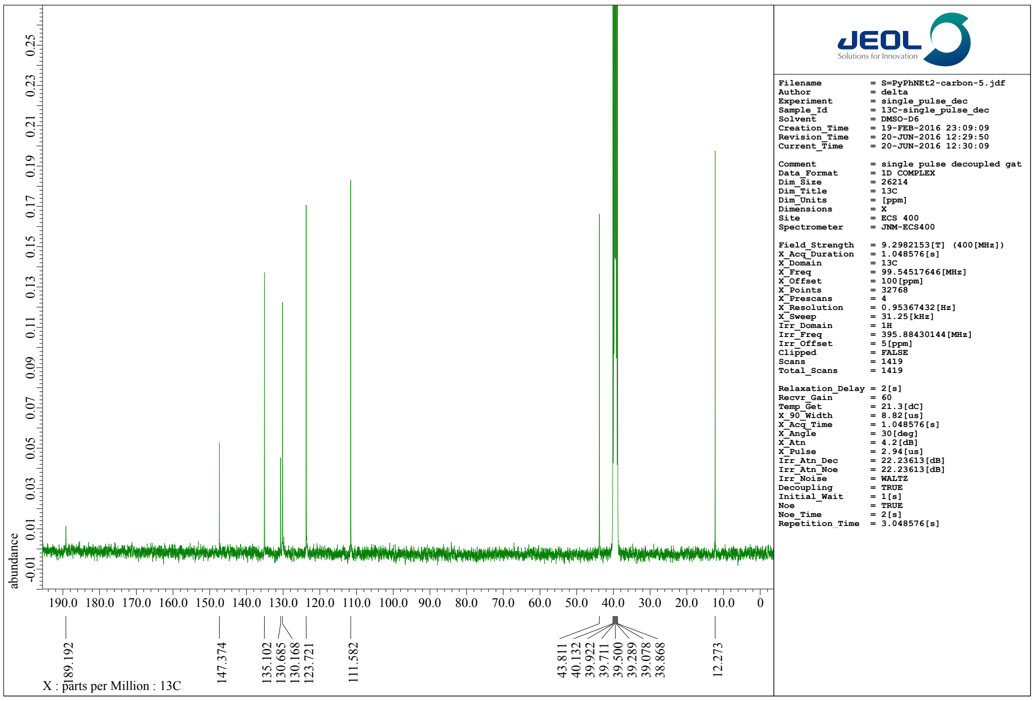


**NMR chart 26**. $^{13}$C-NMR (100 MHz) spectrum of **4b** in dimethyl sulfoxide-*d*$_6$ (*d*-DMSO).

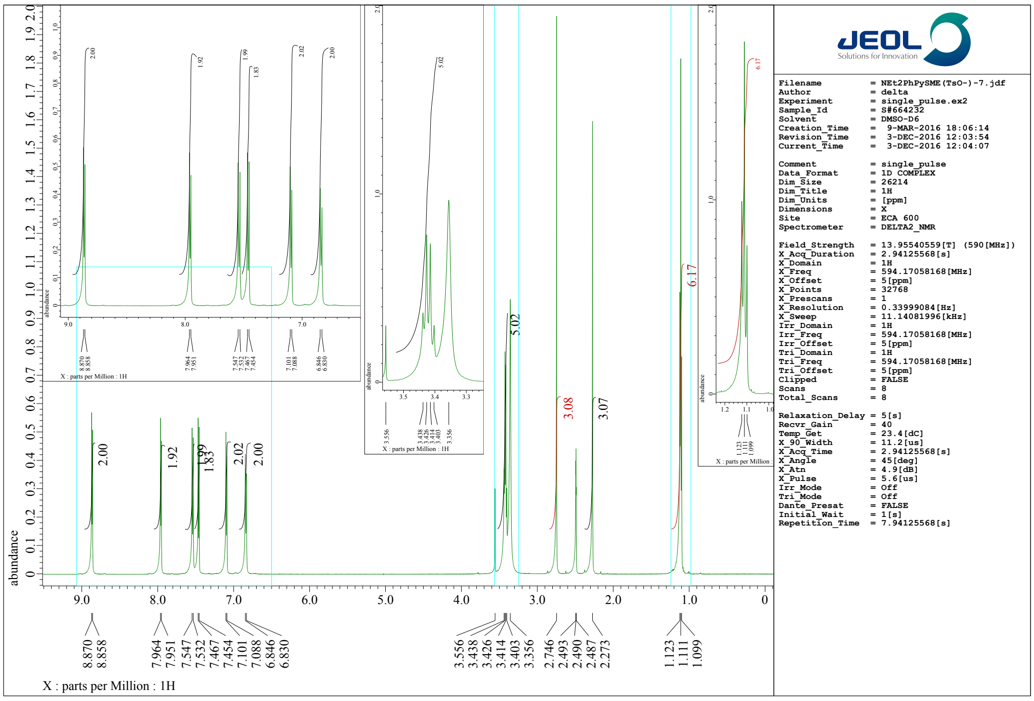


**NMR chart 27**. $^{1}$H-NMR (600 MHz) spectrum of **4c** in dimethyl sulfoxide-*d*$_6$ (*d*-DMSO).

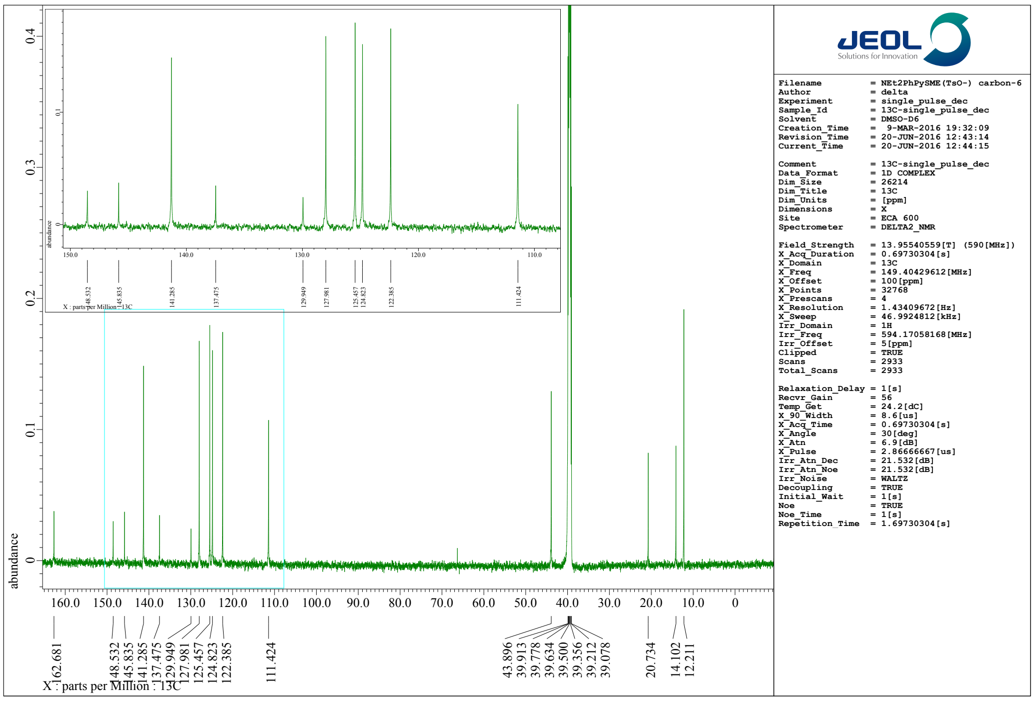


**NMR chart 28**. $^{13}$C-NMR (150 MHz) spectrum of **4c** in dimethyl sulfoxide-*d*$_6$ (*d*-DMSO).

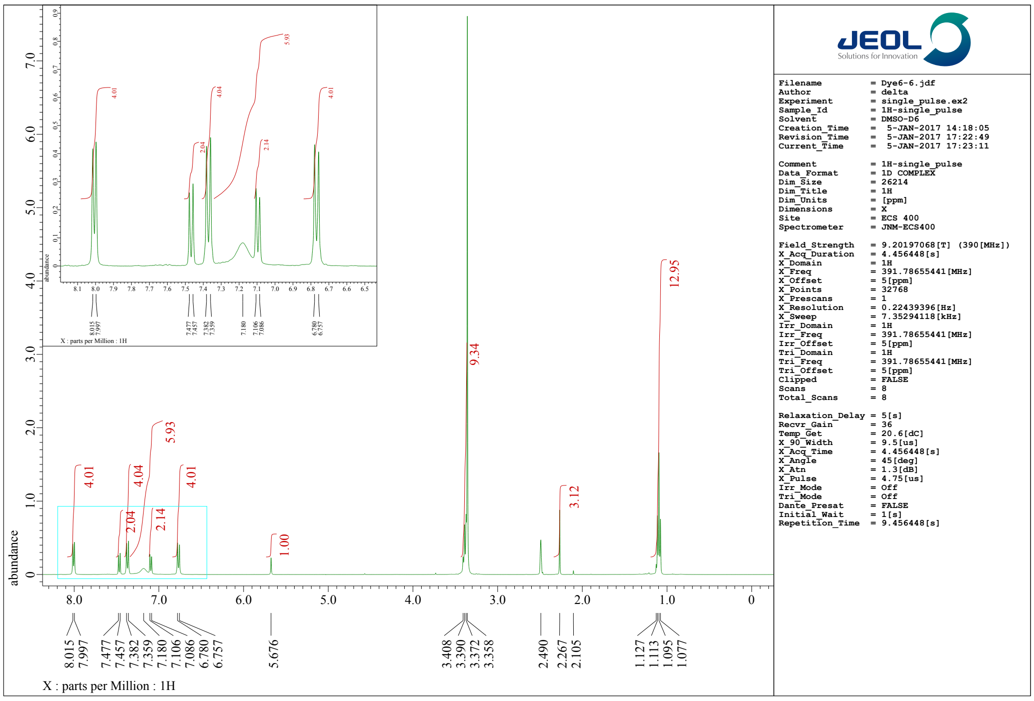


**NMR chart 29**. $^{1}$H-NMR (400 MHz) spectrum of **PC4** in dimethyl sulfoxide-$d_6$ ($d$-DMSO).

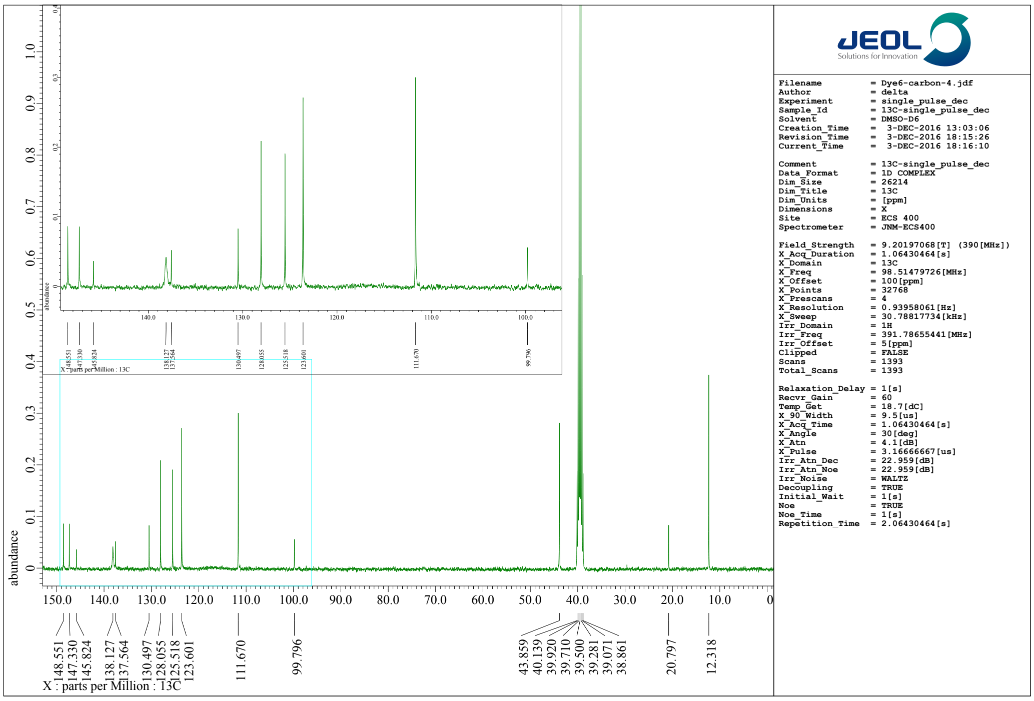


**NMR chart 30**. $^{13}$C-NMR (100 MHz) spectrum of **PC4** in dimethyl sulfoxide-$d_6$ ($d$-DMSO).

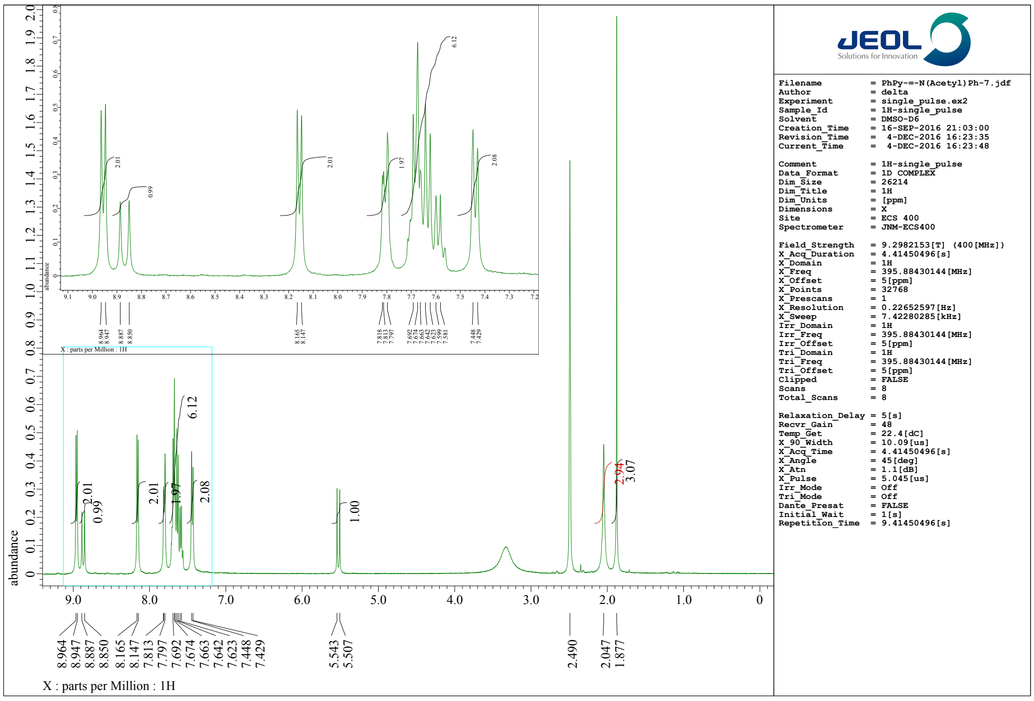


**NMR chart 31**. $^{1}$H-NMR (400 MHz) spectrum of **5c** in dimethyl sulfoxide-$d_6$ ($d$-DMSO).

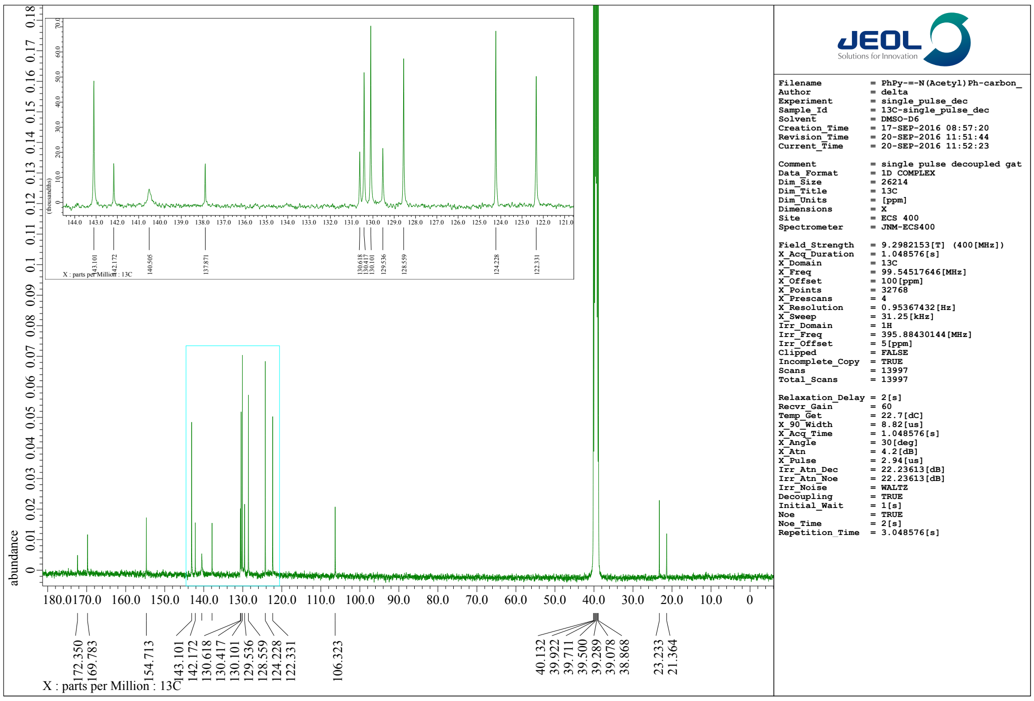


**NMR chart 32**. $^{13}$C-NMR (100 MHz) spectrum of **5c** in dimethyl sulfoxide-$d_6$ ($d$-DMSO).

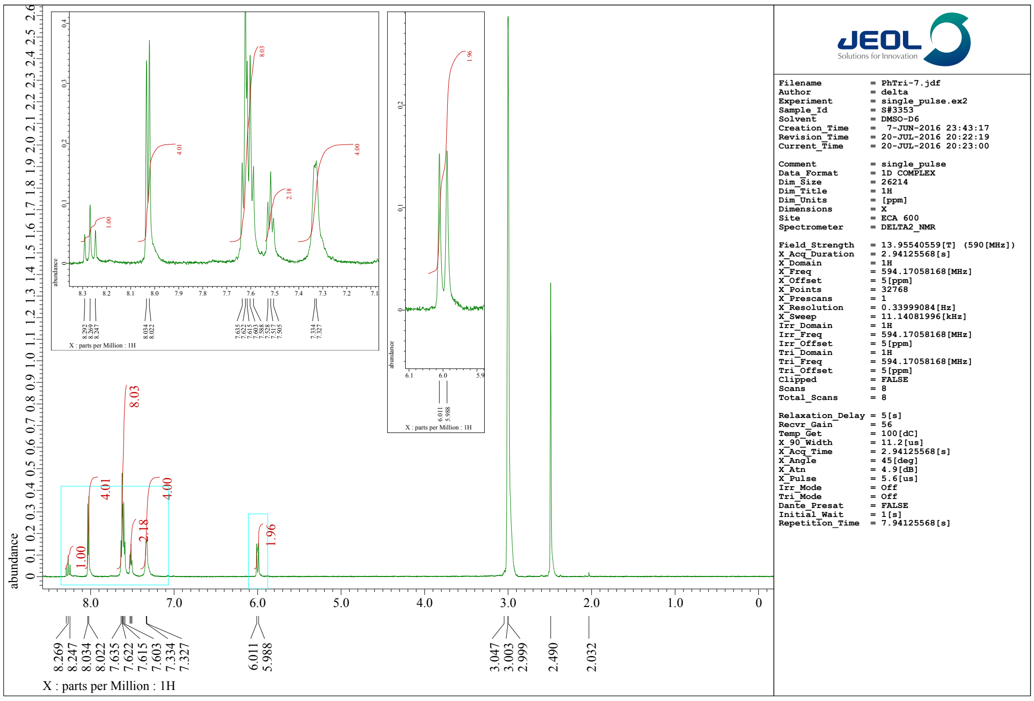


**NMR chart 33**. $^{1}$H-NMR (600 MHz) spectrum of **PC5** in dimethyl sulfoxide-$d_6$ (*d*-DMSO).

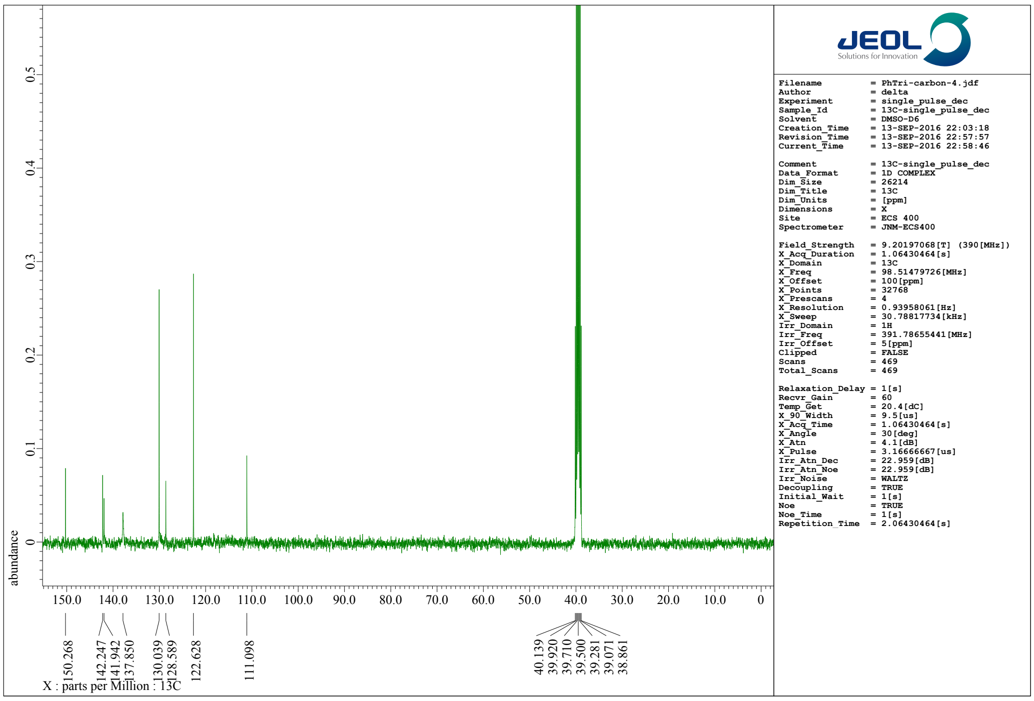


**NMR chart 34**. $^{13}$C-NMR (100 MHz) spectrum of **PC5** in dimethyl sulfoxide-$d_6$ (*d*-DMSO).

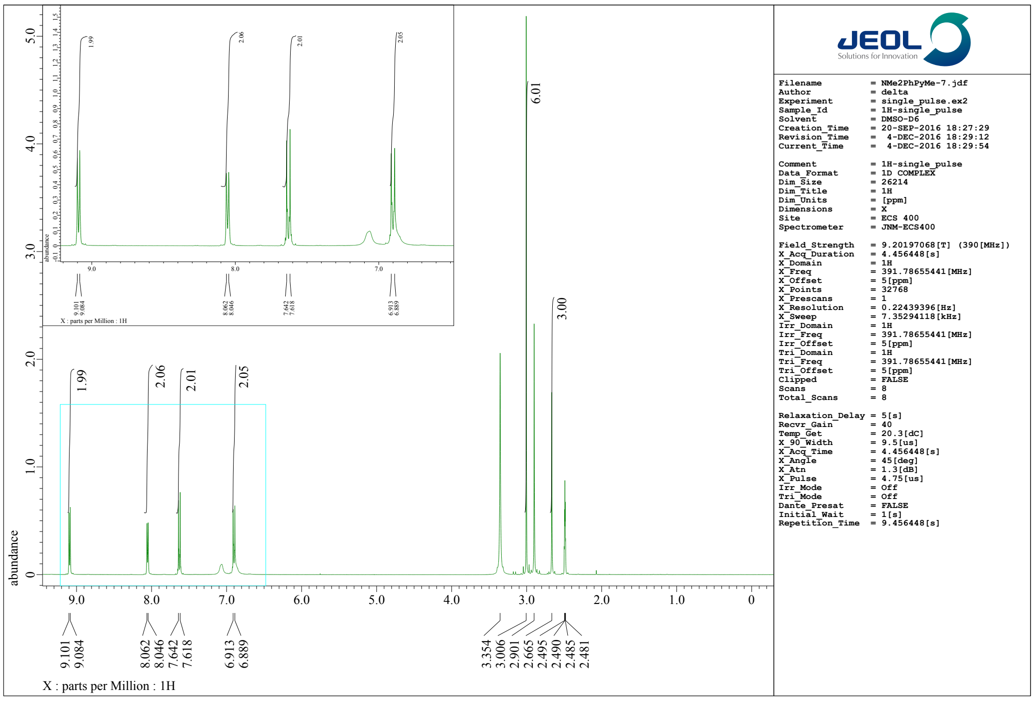


**NMR chart 35**. $^{1}H$-NMR (400 MHz) spectrum of **6a** in dimethyl sulfoxide-*d6* (*d*-DMSO).

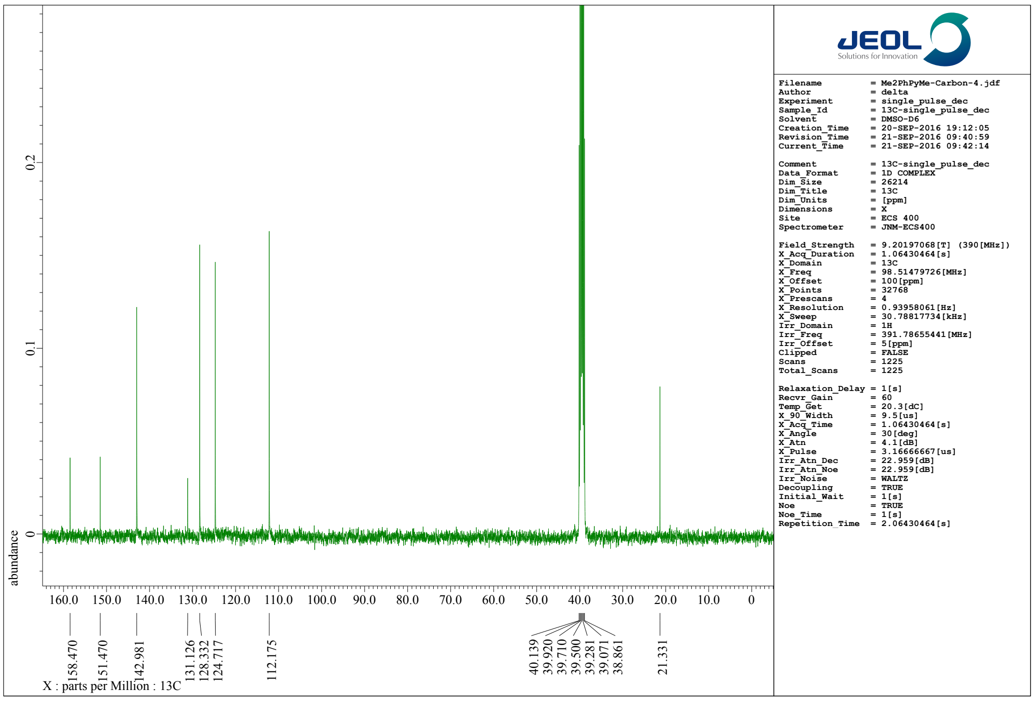


**NMR chart 36**. $^{13}C$-NMR (100 MHz) spectrum of **6a** in dimethyl sulfoxide-d6 (d-DMSO)

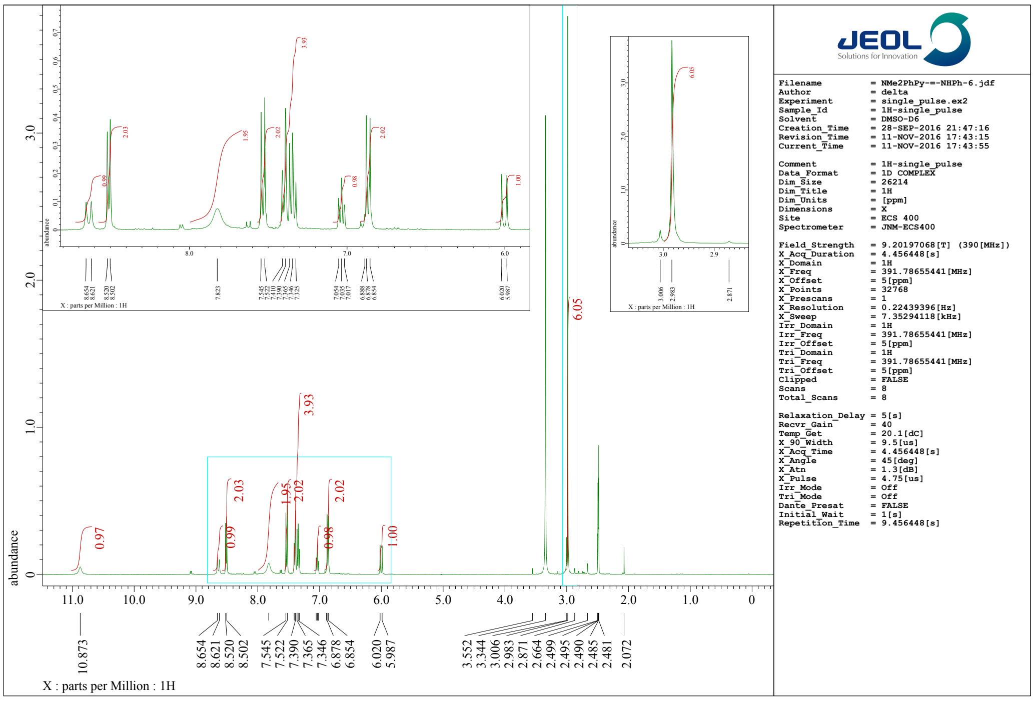


**NMR chart 37**. $^{1}$H-NMR (400 MHz) spectrum of **6b** in dimethyl sulfoxide-*d*$_6$ (*d*-DMSO).

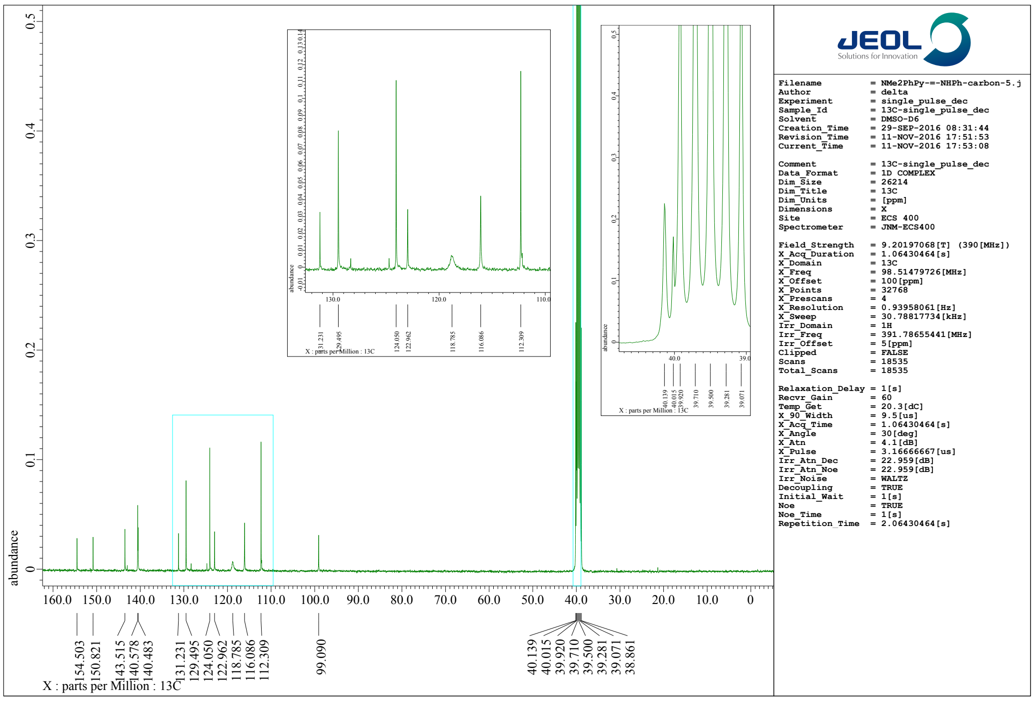


**NMR chart 38**. $^{13}$C-NMR (100 MHz) spectrum of **6b** in dimethyl sulfoxide-*d*$_6$ (*d*-DMSO).

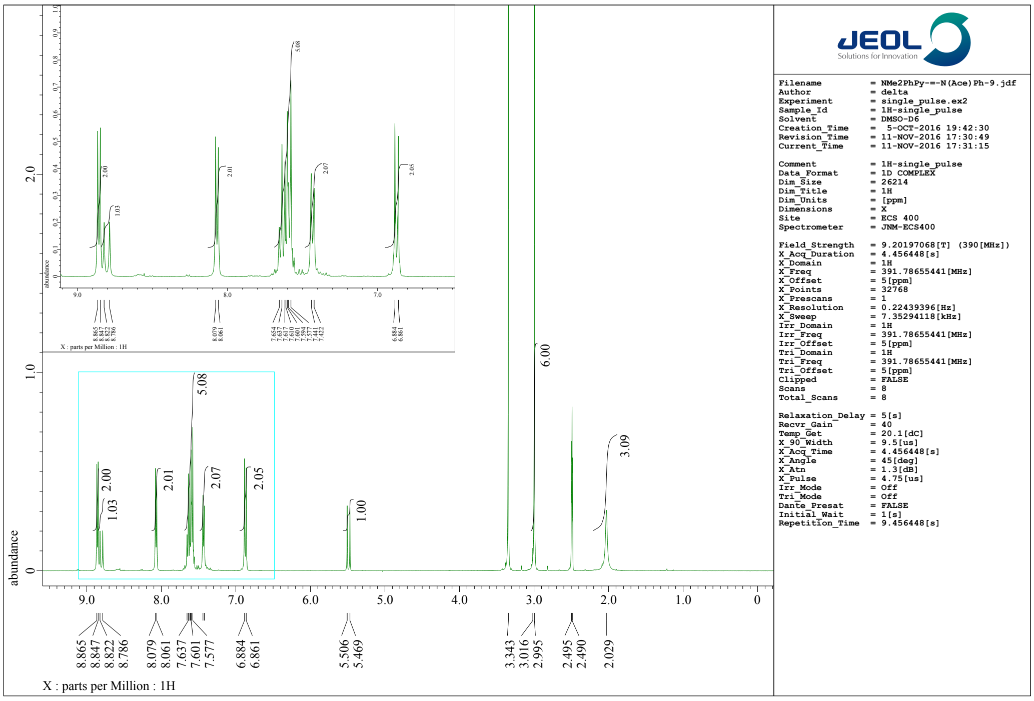

**NMR chart 39**. $^{1}$H-NMR (400 MHz) spectrum of **6c** in dimethyl sulfoxide-$d_6$ (*d*-DMSO).

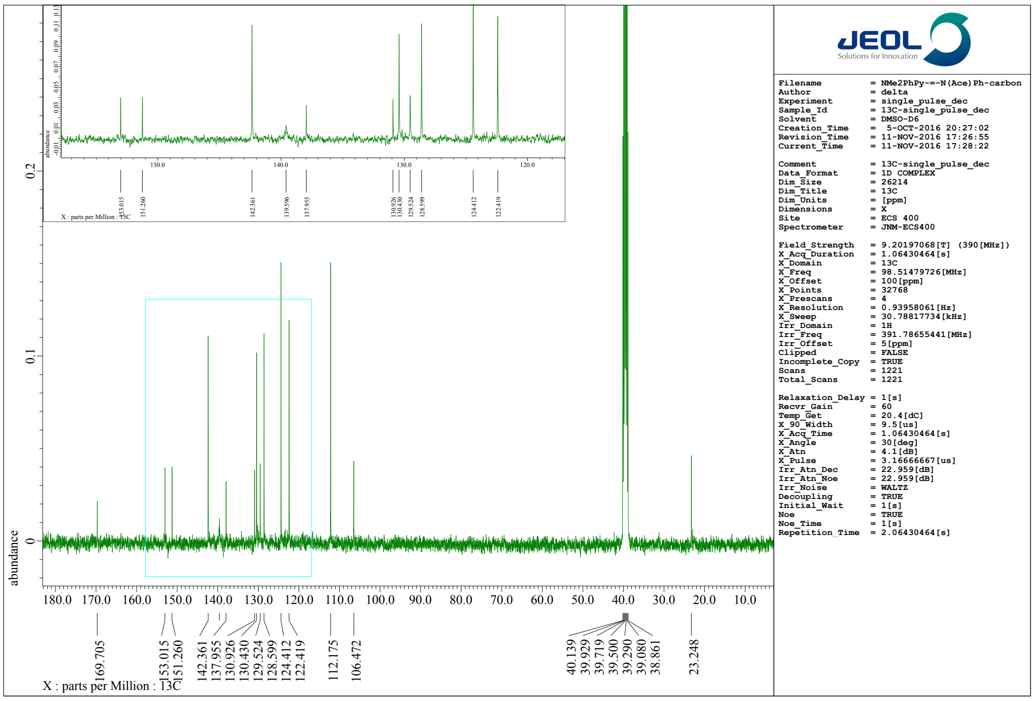

**NMR chart 40**. $^{13}$C-NMR (100 MHz) spectrum of **6c** in dimethyl sulfoxide-$d_6$ (*d*-DMSO).

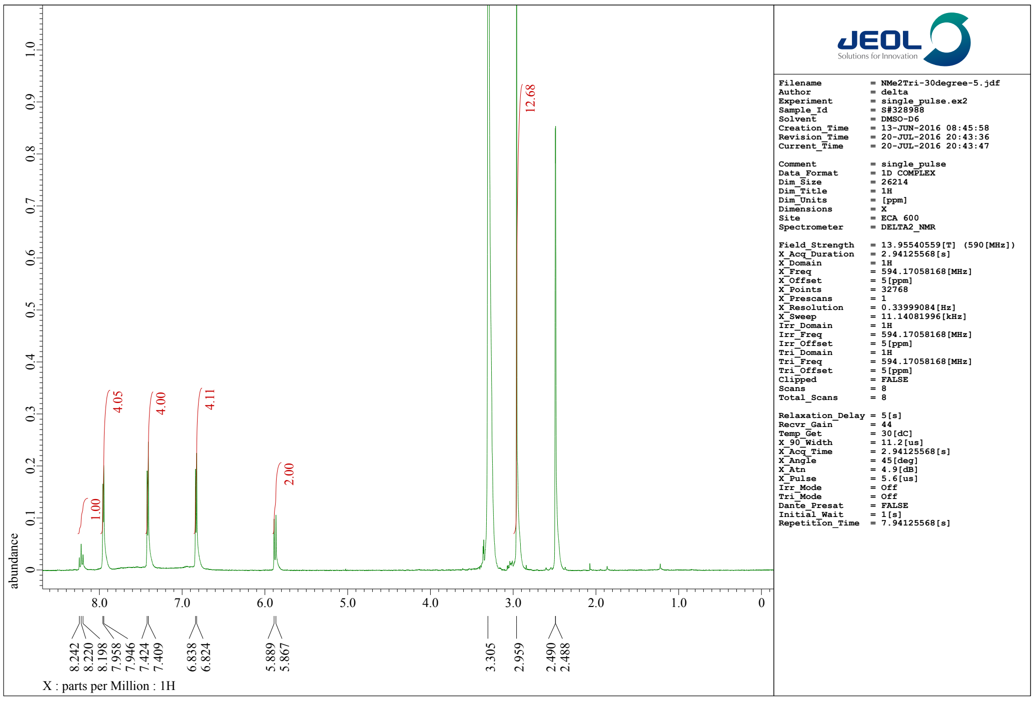


**NMR chart 41**. $^{1}$H-NMR (600 MHz) spectrum of **PC6** in dimethyl sulfoxide-$d_6$ ($d$-DMSO).

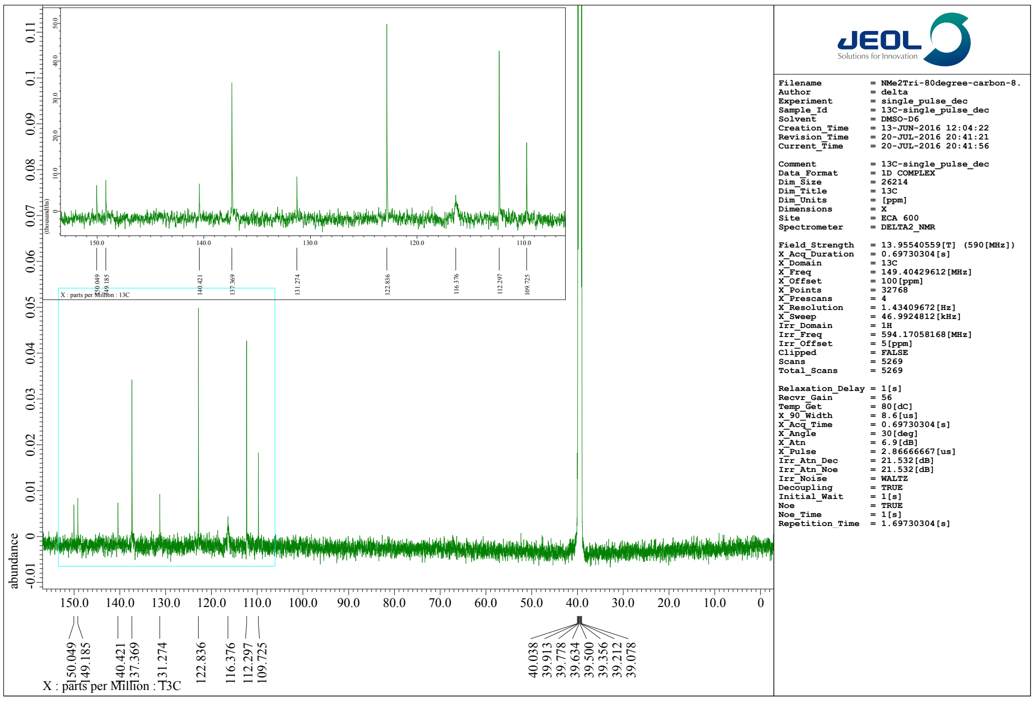


**NMR chart 42**. $^{13}$C-NMR (150 MHz) spectrum of **PC6** in dimethyl sulfoxide-$d_6$ ($d$-DMSO).

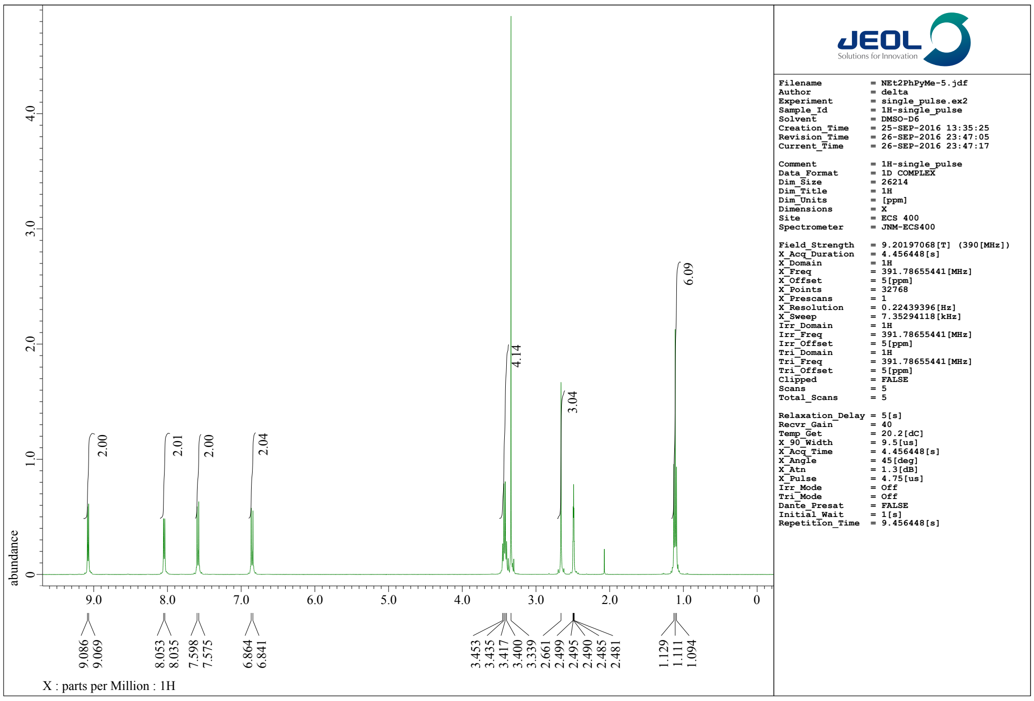


**NMR chart 43**. $^{1}$H-NMR (400 MHz) spectrum of **7a** in dimethyl sulfoxide-$d_6$ ($d$-DMSO).

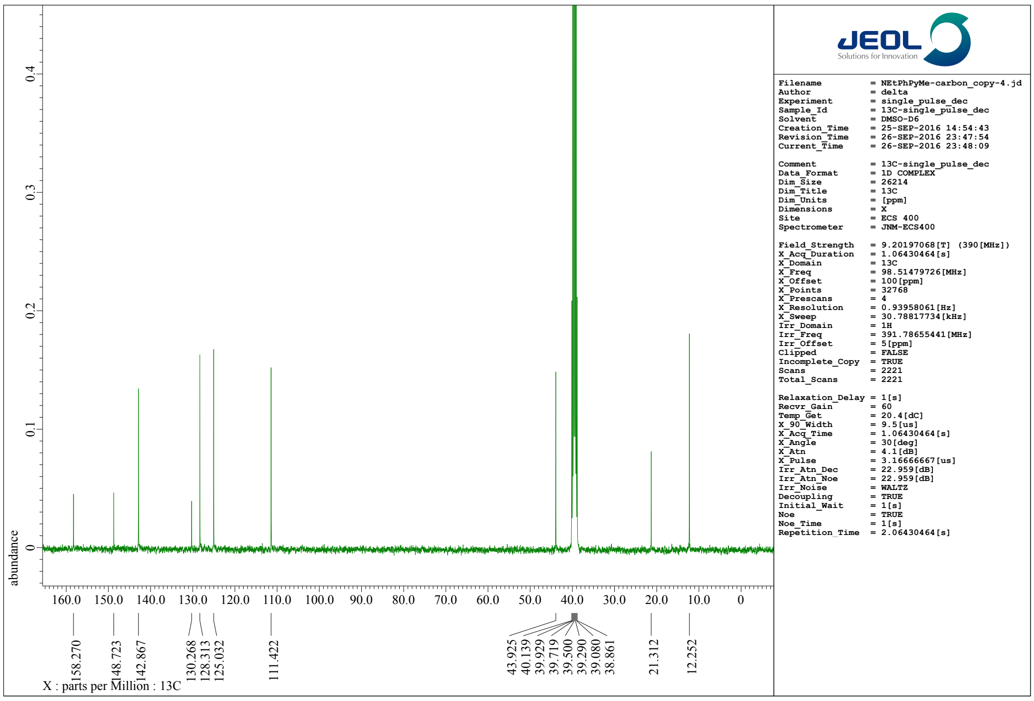


**NMR chart 44**. $^{13}$C-NMR (100 MHz) spectrum of **7a** in dimethyl sulfoxide-$d_6$ ($d$-DMSO).

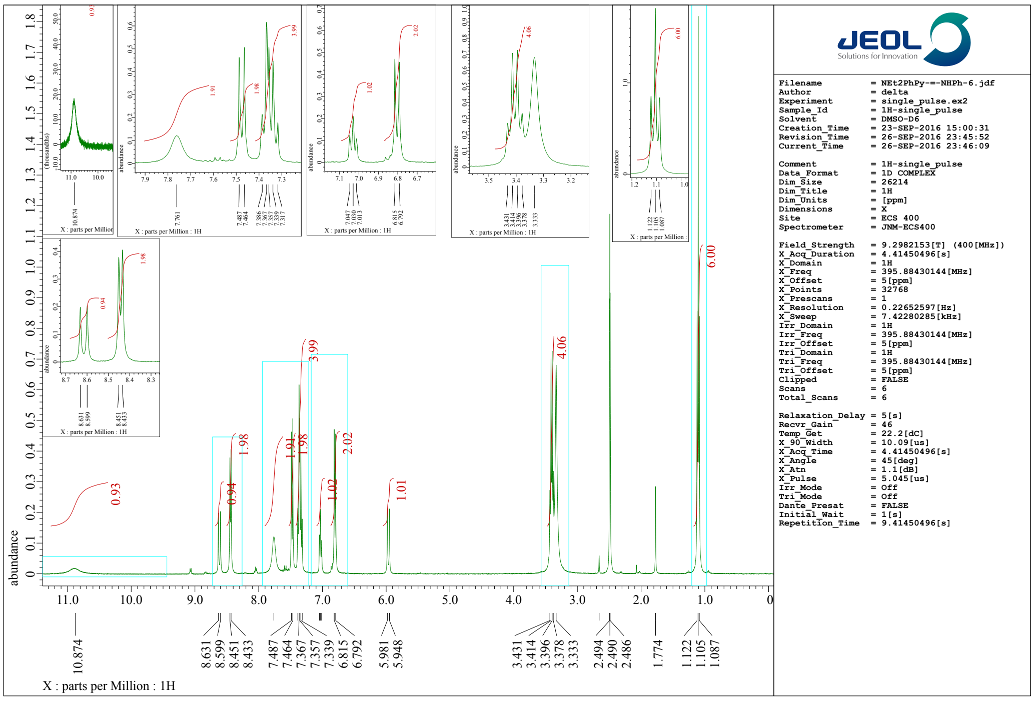

**NMR chart 45**. $^{1}$H-NMR (400 MHz) spectrum of **7b** in dimethyl sulfoxide-d6 (d-DMSO)

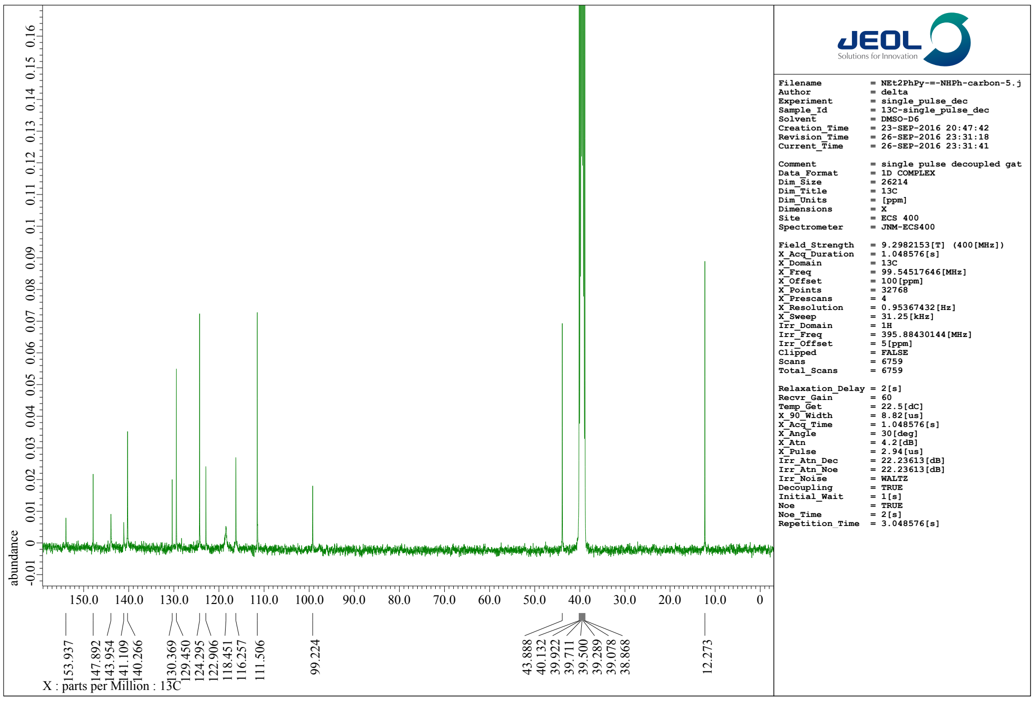

**NMR chart 46**. $^{13}$C-NMR (100 MHz) spectrum of **7b** in dimethyl sulfoxide-$d_6$ (*d*-DMSO).

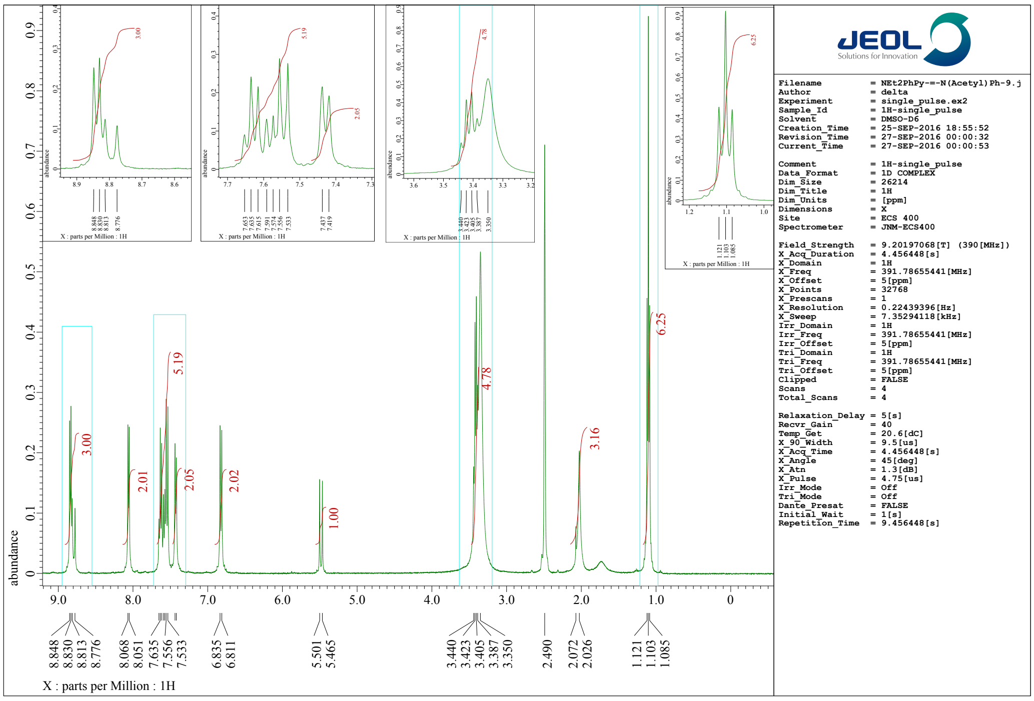

**NMR chart 47**. $^{1}$H-NMR (400 MHz) spectrum of **7c** in dimethyl sulfoxide-$d_6$ ($d$-DMSO).

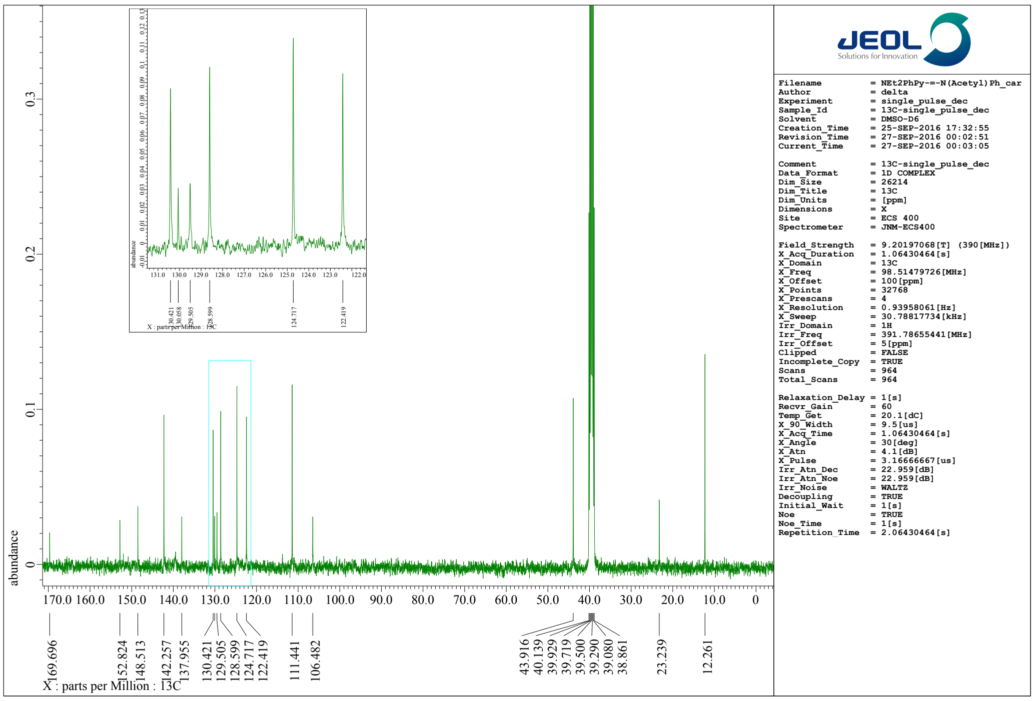

**NMR chart 48**. $^{13}$C-NMR (100 MHz) spectrum of **7c** in dimethyl sulfoxide-$d_6$ ($d$-DMSO).

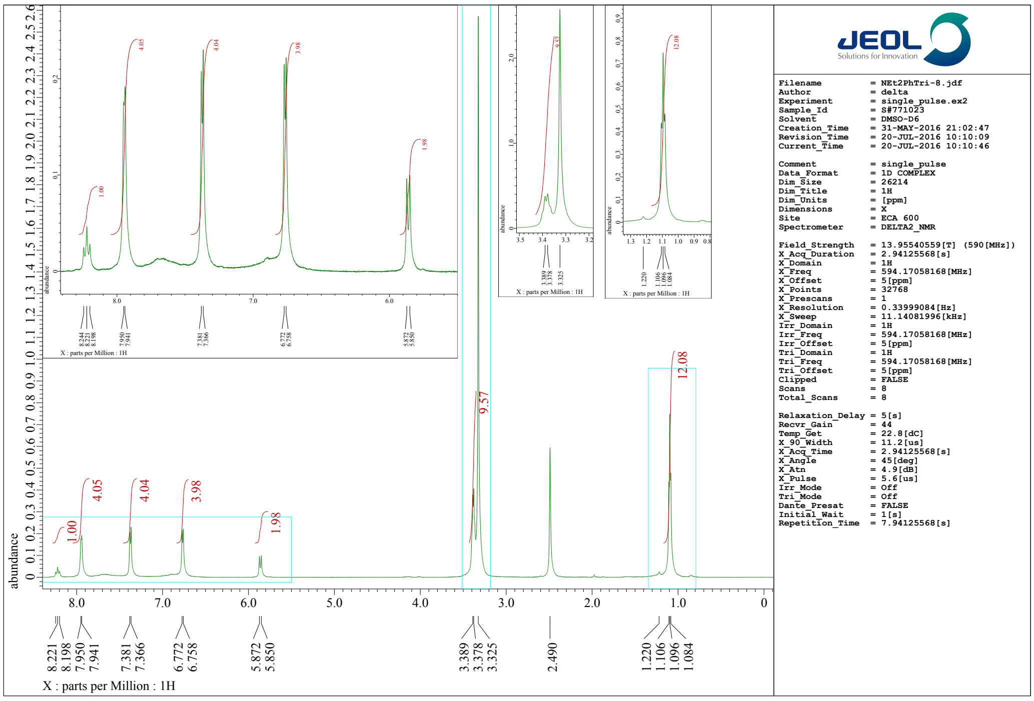


**NMR chart 49**. $^{1}$H-NMR (600 MHz) spectrum of **PC7** in dimethyl sulfoxide-$d_6$ (*d*-DMSO).

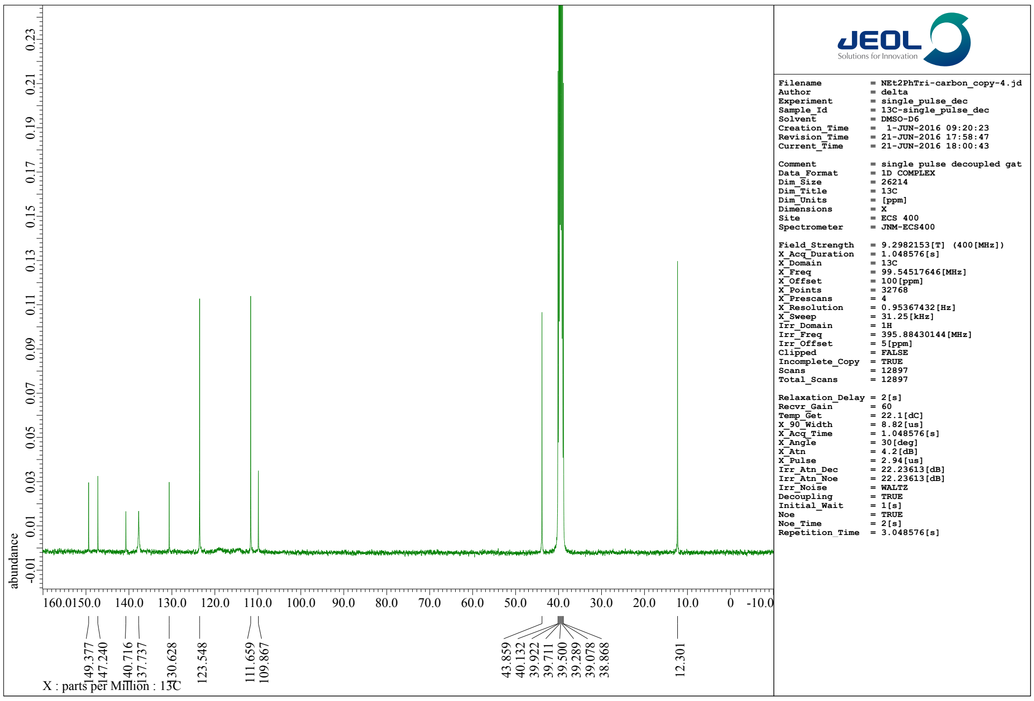


**NMR chart 50**. $^{13}$C-NMR (100 MHz) spectrum of **PC7** in dimethyl sulfoxide-$d_6$ (*d*-DMSO).

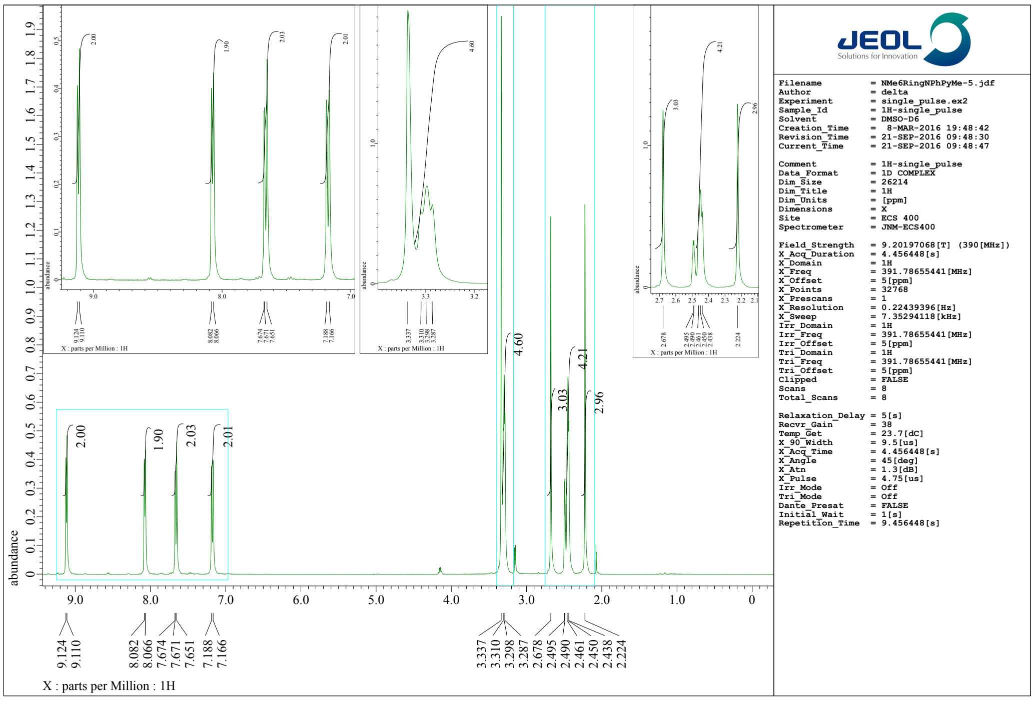


**NMR chart 51**. $^{1}$H-NMR (400 MHz) spectrum of **8a** in dimethyl sulfoxide-*d₆* (*d*-DMSO).

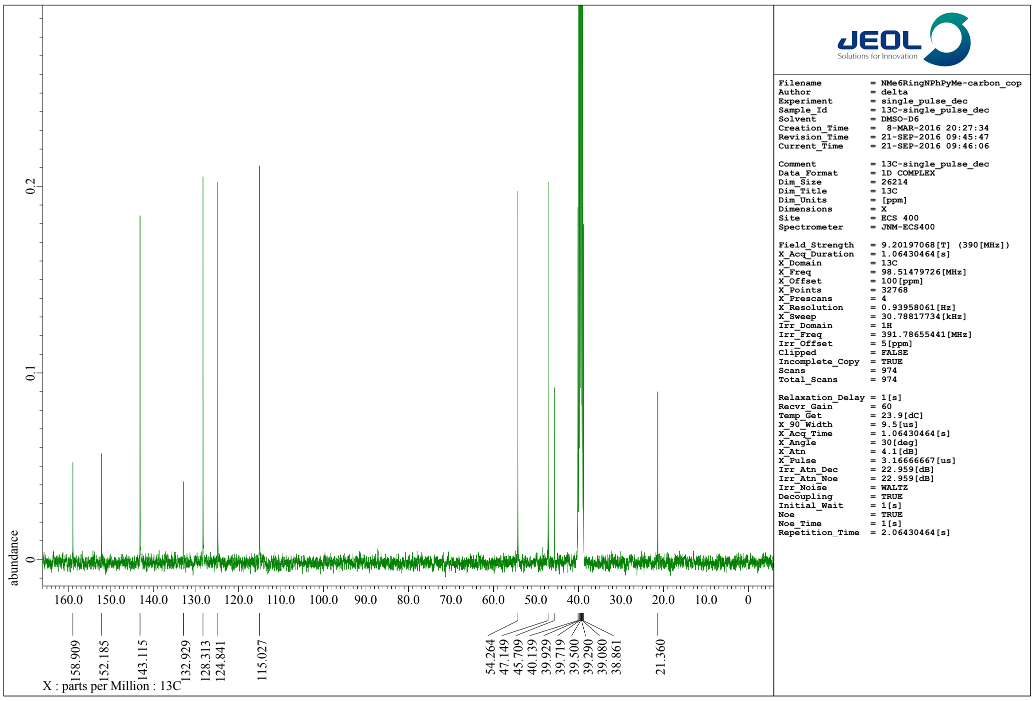


**NMR chart 52**. $^{13}$C-NMR (100 MHz) spectrum of **8a** in dimethyl sulfoxide-*d₆* (*d*-DMSO).

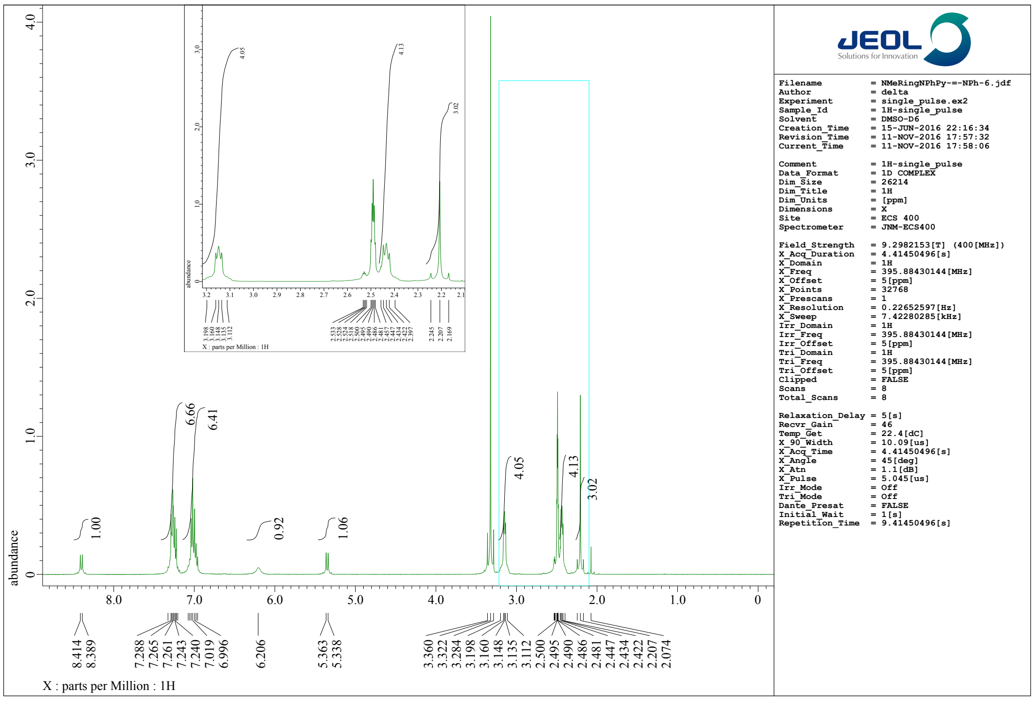


**NMR chart 53**. $^{1}$H-NMR (400 MHz) spectrum of **8b** in dimethyl sulfoxide-$d_6$ (*d*-DMSO).

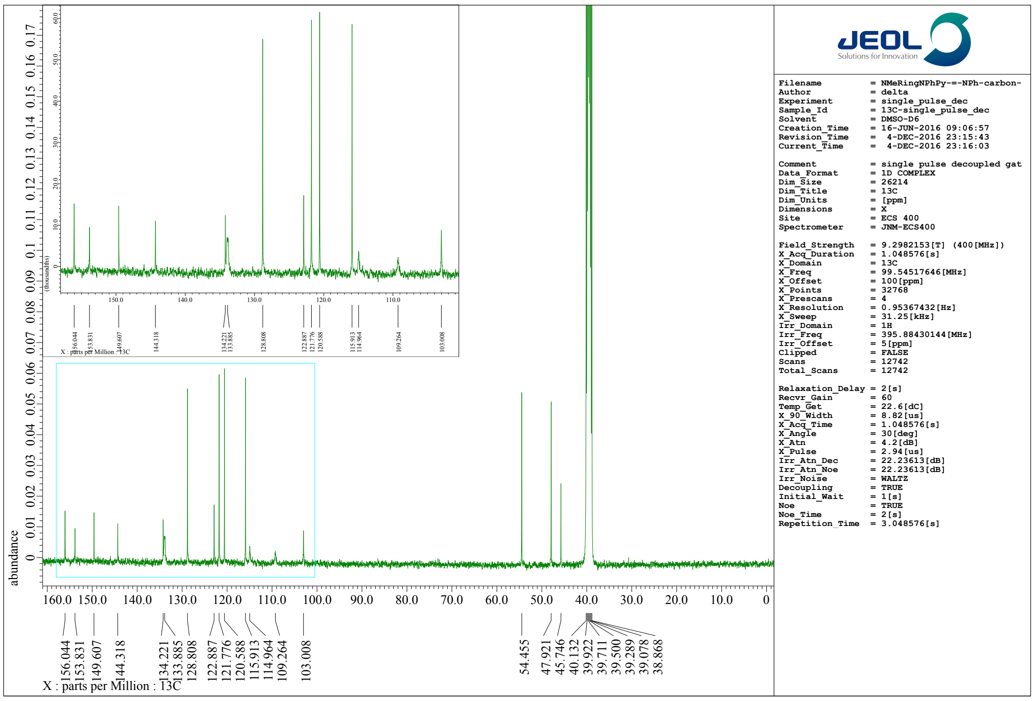


**NMR chart 54**. $^{13}$C-NMR (100 MHz) spectrum of **8b** in dimethyl sulfoxide-$d_6$ (*d*-DMSO).

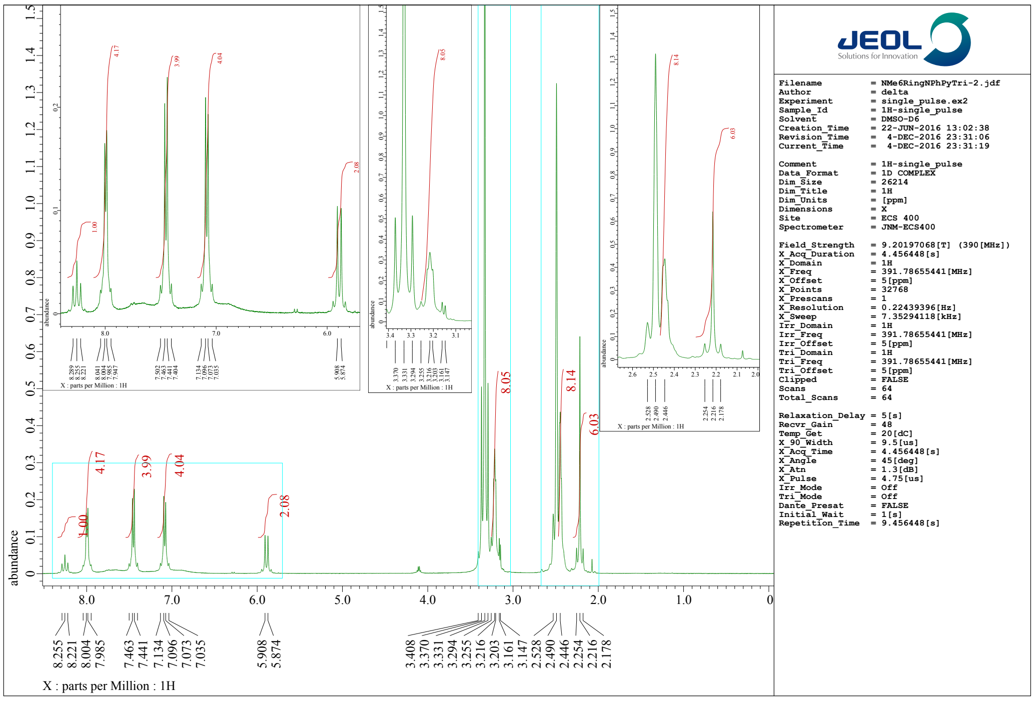


**NMR chart 55**. $^{1}$H-NMR (400 MHz) spectrum of **PC8** in dimethyl sulfoxide-$d_6$ (*d*-DMSO).

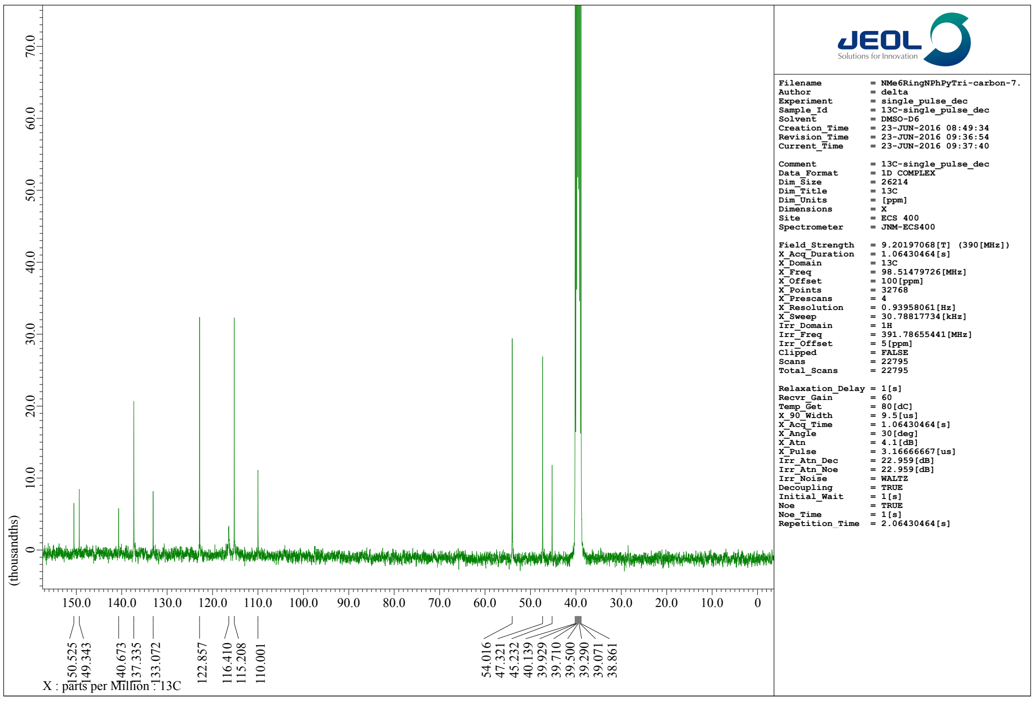


**NMR chart 56**. $^{13}$C-NMR (100 MHz) spectrum of **PC8** in dimethyl sulfoxide-$d_6$ (*d*-DMSO).

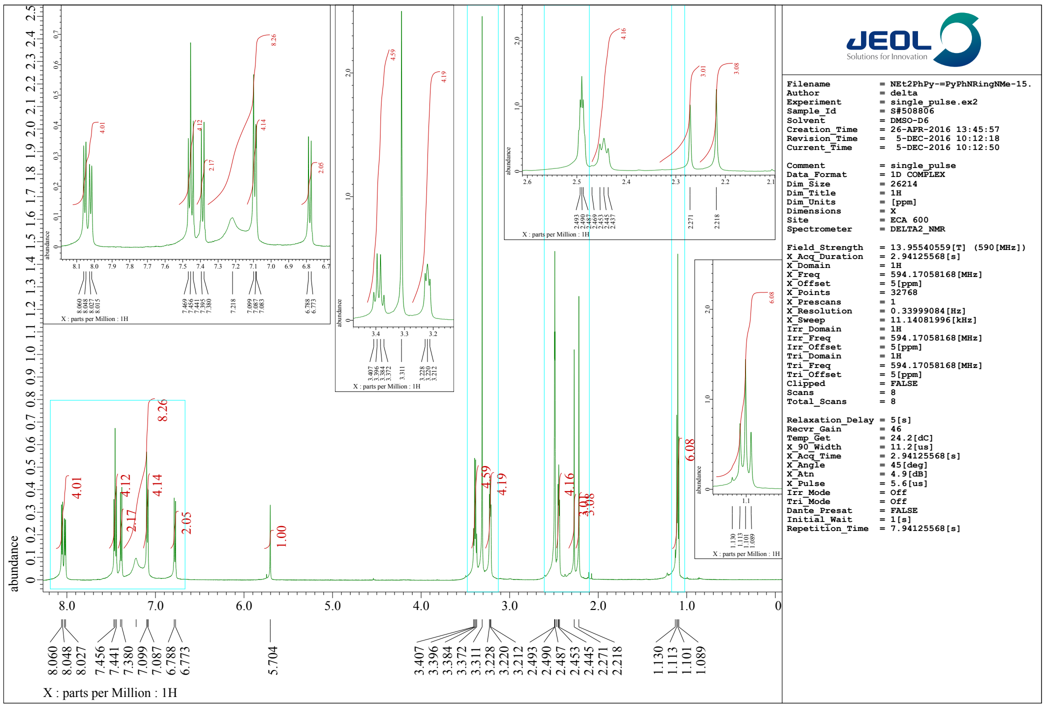


**NMR chart 57**. $^{1}$H-NMR (600 MHz) spectrum of **PC9** in dimethyl sulfoxide-$d_6$ ($d$-DMSO).

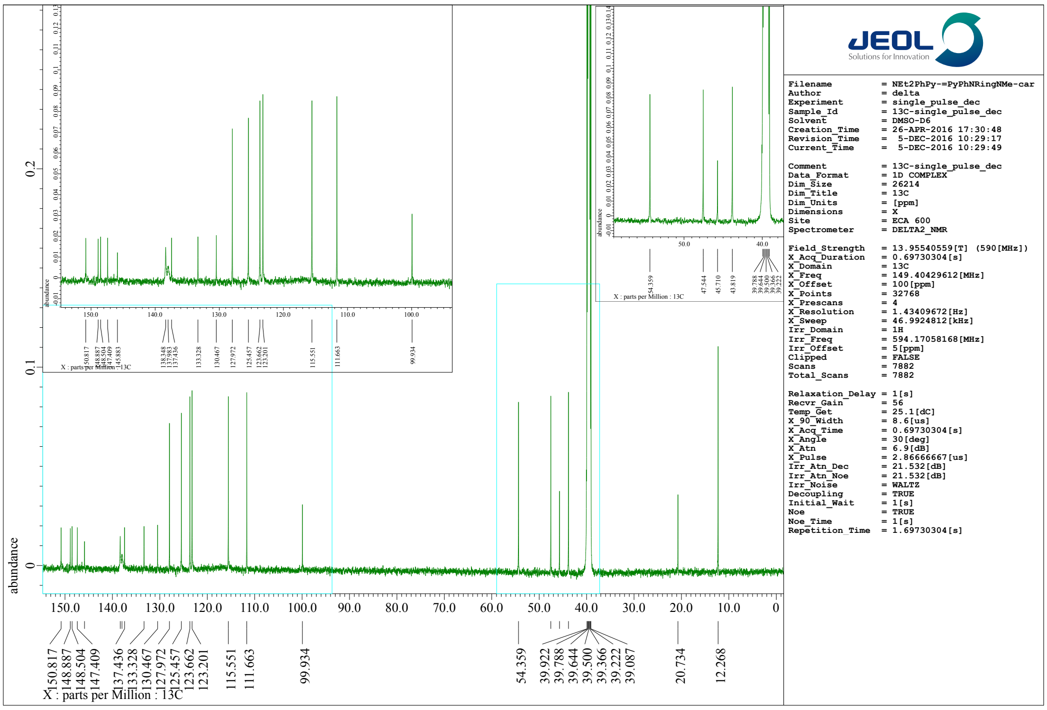


**NMR chart 58**. $^{13}$C-NMR (150 MHz) spectrum of **PC9** in dimethyl sulfoxide-$d_6$ ($d$-DMSO).
