# Supplementary material for: N-aryl pyrido cyanine derivatives are nuclear and organelle DNA markers for two-photon and super-resolution imaging
Source: Nat Commun. 2021 May 11;12:2650. doi: 10.1038/s41467-021-23019-w (PMC8113587; doi:10.1038/s41467-021-23019-w)
Supplement: Supplementary file 3 — Reporting Summary [file 41467_2021_23019_MOESM3_ESM.pdf]

nature research

Corresponding author(s): Yoshikatsu Sato

Last updated by author(s): Apr 6, 2021

Reporting Summary

Nature Research wishes to improve the reproducibility of the work that we publish. This form provides structure for consistency and transparency in reporting. For further information on Nature Research policies, see our Editorial Policies and the Editorial Policy Checklist.

Statistics

For all statistical analyses, confirm that the following items are present in the figure legend, table legend, main text, or Methods section.

n/a	Confirmed	
☐	☒	The exact sample size (n) for each experimental group/condition, given as a discrete number and unit of measurement
☐	☒	A statement on whether measurements were taken from distinct samples or whether the same sample was measured repeatedly
☐	☒	The statistical test(s) used AND whether they are one- or two-sided<br>*Only common tests should be described solely by name; describe more complex techniques in the Methods section.*
☒	☐	A description of all covariates tested
☐	☒	A description of any assumptions or corrections, such as tests of normality and adjustment for multiple comparisons
☐	☒	A full description of the statistical parameters including central tendency (e.g. means) or other basic estimates (e.g. regression coefficient) AND variation (e.g. standard deviation) or associated estimates of uncertainty (e.g. confidence intervals)
☐	☒	For null hypothesis testing, the test statistic (e.g. F, t, r) with confidence intervals, effect sizes, degrees of freedom and P value noted<br>*Give P values as exact values whenever suitable.*
☒	☐	For Bayesian analysis, information on the choice of priors and Markov chain Monte Carlo settings
☒	☐	For hierarchical and complex designs, identification of the appropriate level for tests and full reporting of outcomes
☒	☐	Estimates of effect sizes (e.g. Cohen's d, Pearson's r), indicating how they were calculated

Our web collection on statistics for biologists contains articles on many of the points above.

Software and code

Policy information about availability of computer code

Data collection	Microscope: LSM 7 Duo ver. 8.1.11.484 ZEN 2012 SP1(Zeiss), SP8 gSTED Falcon system LAS X ver. 3.5.5.19976(Leica), Metamorph ver. 7.10.1.161 (Molecular Devices)<br>UV/Vis absorption spectra: UV-3510 spectrometer (Shimadzu)<br>Emission spectra: FP-6600 spectrometer (Hitachi)<br>Fluorescence quantum yield: C9920-02 (Hamamatsu)<br>Fluorescence lifetime: SPC-130EM-N1 (Becker & Hickl)<br>Circular Dichroism: FT/IR6100 (JASCO)
Data analysis	Microscopy: Huygens Professional ver.18.10.0p8 64b, ImageJ 1.48k (http://rsb.info.nih.gov/ij/), Photoshop CS6 ver. 13.0 x64.<br>Statistics analysis: Mirosoft Excel version 16.16.25

For manuscripts utilizing custom algorithms or software that are central to the research but not yet described in published literature, software must be made available to editors and reviewers. We strongly encourage code deposition in a community repository (e.g. GitHub). See the Nature Research guidelines for submitting code & software for further information.

Data

Policy information about availability of data

All manuscripts must include a data availability statement. This statement should provide the following information, where applicable:

- Accession codes, unique identifiers, or web links for publicly available datasets
- A list of figures that have associated raw data
- A description of any restrictions on data availability

The authors declare that all other data of this study are available within the manuscript and its supplementary files are available from the corresponding author

upon request.

Field-specific reporting

Please select the one below that is the best fit for your research. If you are not sure, read the appropriate sections before making your selection.

☒ Life sciences ☐ Behavioural & social sciences ☐ Ecological, evolutionary & environmental sciences

For a reference copy of the document with all sections, see nature.com/documents/nr-reporting-summary-flat.pdf

Life sciences study design

All studies must disclose on these points even when the disclosure is negative.

Sample size	For cell proliferation assay (Fig. 2b), the sample size was chosen to be the number of cells in a field of view using a 20x objective lens, because it is a necessary and sufficient magnification for cell counting. We performed time-lapse experiments at three times with 2 multiple data points. We believe that the sample size was sufficient to compare the cell toxicity with commercially available dyes. For FWHM analysis (Fig. 4d), we chose five mt-DNA were measured per cell from three independent cells, for a total of 15 mt-DNA. We are confident that the sample size was enough to show the time-gate effects on enhancement of the resolution. For fluorescent lifetime analysis (Fig. S9b), we chose five independent cells and measured the fluorescent lifetime in mt-DNA and nuclear DNA. We believe that the sample size was enough to show the changes of fluorescent lifetime dependent on the dye concentration.
Data exclusions	No data was excluded from all experiments.
Replication	We performed all experiments at least three replication using different biological samples for statistical analysis. As for DNA staining, at least two images of defferent cells or tissues were taken. Similar images were obtained in all attempts.
Randomization	The sample was not fixed at the specified position when we observed samples in multi-well bottomed dish, and the allocation is considered to be random.
Blinding	The investigators were blinded to chemical structure during data collection and analysis.

Reporting for specific materials, systems and methods

We require information from authors about some types of materials, experimental systems and methods used in many studies. Here, indicate whether each material, system or method listed is relevant to your study. If you are not sure if a list item applies to your research, read the appropriate section before selecting a response.

Materials & experimental systems

n/a	Involved in the study
☒	☐ Antibodies
☐	☒ Eukaryotic cell lines
☒	☐ Palaeontology and archaeology
☒	☐ Animals and other organisms
☒	☐ Human research participants
☒	☐ Clinical data
☒	☐ Dual use research of concern

Methods

n/a	Involved in the study
☒	☐ ChIP-seq
☒	☐ Flow cytometry
☒	☐ MRI-based neuroimaging

Eukaryotic cell lines

Policy information about cell lines

Cell line source(s)	HeLa (RIKEN), C6 (RIKEN), NIH3T3(RIKEN), U-2OS (ATCC)
Authentication	None of the cell lines were authenticated.
Mycoplasma contamination	We confirmed that all cell lines tested were negative for mycoplasma contamination by DNA staining.
Commonly misidentified lines (See ICLAC register)	We declare that no commonly misidentified cell lines were used in the study.
