# Supplementary material for: N-aryl pyrido cyanine derivatives are nuclear and organelle DNA markers for two-photon and super-resolution imaging
Source: Nat Commun. 2021 May 11;12:2650. doi: 10.1038/s41467-021-23019-w (PMC8113587; doi:10.1038/s41467-021-23019-w)
Supplement: Supplementary file 10 — Description of Additional Supplementary Files [file 41467_2021_23019_MOESM10_ESM.pdf]

Title: Supplementary Movie 1.
Description: Staining nuclear DNA in Arabidopsis leaf cells with PC1. Arabidopsis leaf cells were stained with 1 µM PC1. Note that PC1 stains nuclear DNA in mesophyll cells as well as epidermal cells including stomata.

Title: Supplementary Movie 2.
Description: Staining nuclear DNA in Arabidopsis leaf cells with PC3. Arabidopsis leaf cells were stained with 1 µM PC3. Note that PC3 stains nuclear DNA in mesophyll cells as well as epidermal cells including stomata.

Title: Supplementary Movie 3.
Description: Time-lapse analysis of Arabidopsis root and root hairs with 1 µM PC1. Arabidopsis root stained with 1 µM PC1 was observed every 5 min excited with 488 nm and the emission spectrum was collected through band-pass filter BP525/50. The fluorescence images are maximum zprojections of 20 planes (4.3-µm intervals). On the right side, combined images of the PC1 and bright-field images. Note that the growing root hairs appear after the root tip moved through the image area.

Title: Supplementary Movie 4.
Description: Comparison of light penetration for CLSM and 2PEM in Arabidopsis root tip stained with 1 µM PC1. Selected frames every 10 µm are shown in Fig. 2e

Title: Supplementary Movie 5.
Description: Time-lapse observation by two-photon microscopy excited with 1000 nm in Arabidopsis root stained with PC1. Root tip stained with 5 µM PC1 was observed every 2 min with z-sectioning (50 frames at 2 µm steps). The images are maximum z-projections of middle 25 planes. Selected frames every 10 min between 40 – 80 min are shown in Fig. 2e

Title: Supplementary Movie 6.
Description: Discrimination between nuclear DNA, mt-DNA, and chl-DNA with fluorescence lifetime of PC-1. Arabidopsis leaf cells were stained with 300 nM PC1. The fluorescent life times of mt-DNA (0.882 ns), chl-DNA (0.562 ns) and nuclear DNA (1.199 ns) are indicated in cyan, yellow and magenta, respectively. The Maximum projected image was shown in Fig. 3k.
